# Supplementary figures and images for: The amyloid precursor protein is a conserved Wnt receptor
Source: eLife. 2021 Sep 9;10:e69199. doi: 10.7554/eLife.69199 (PMC8437438; doi:10.7554/eLife.69199)

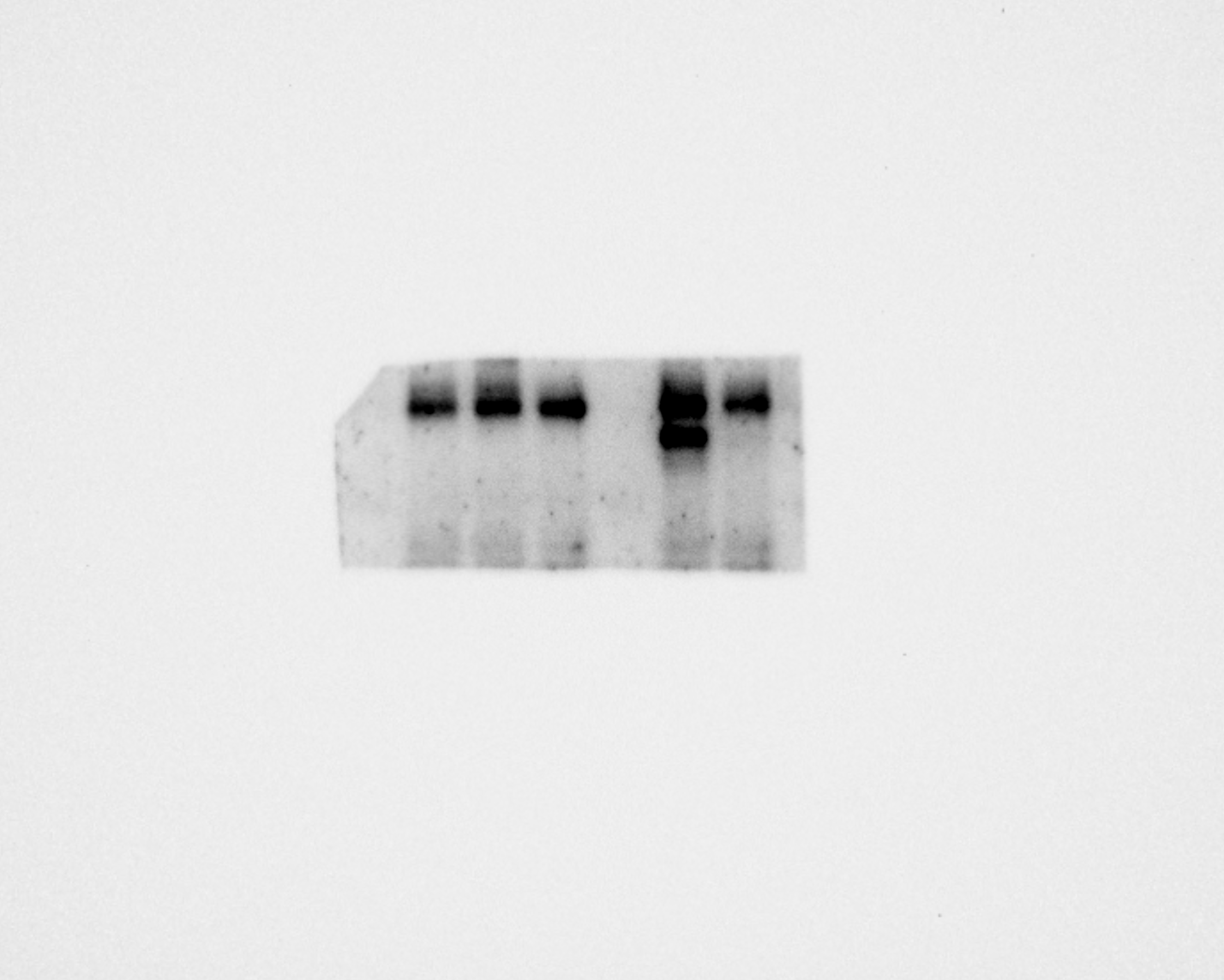

Supplement: Figure 3—source data 1. [file elife-69199-fig3-data1.zip › Figure 3A_Source Data/Figure_3A-Source_data_1_raw_ipFlag_anti_Myc_immunoblot.tif]

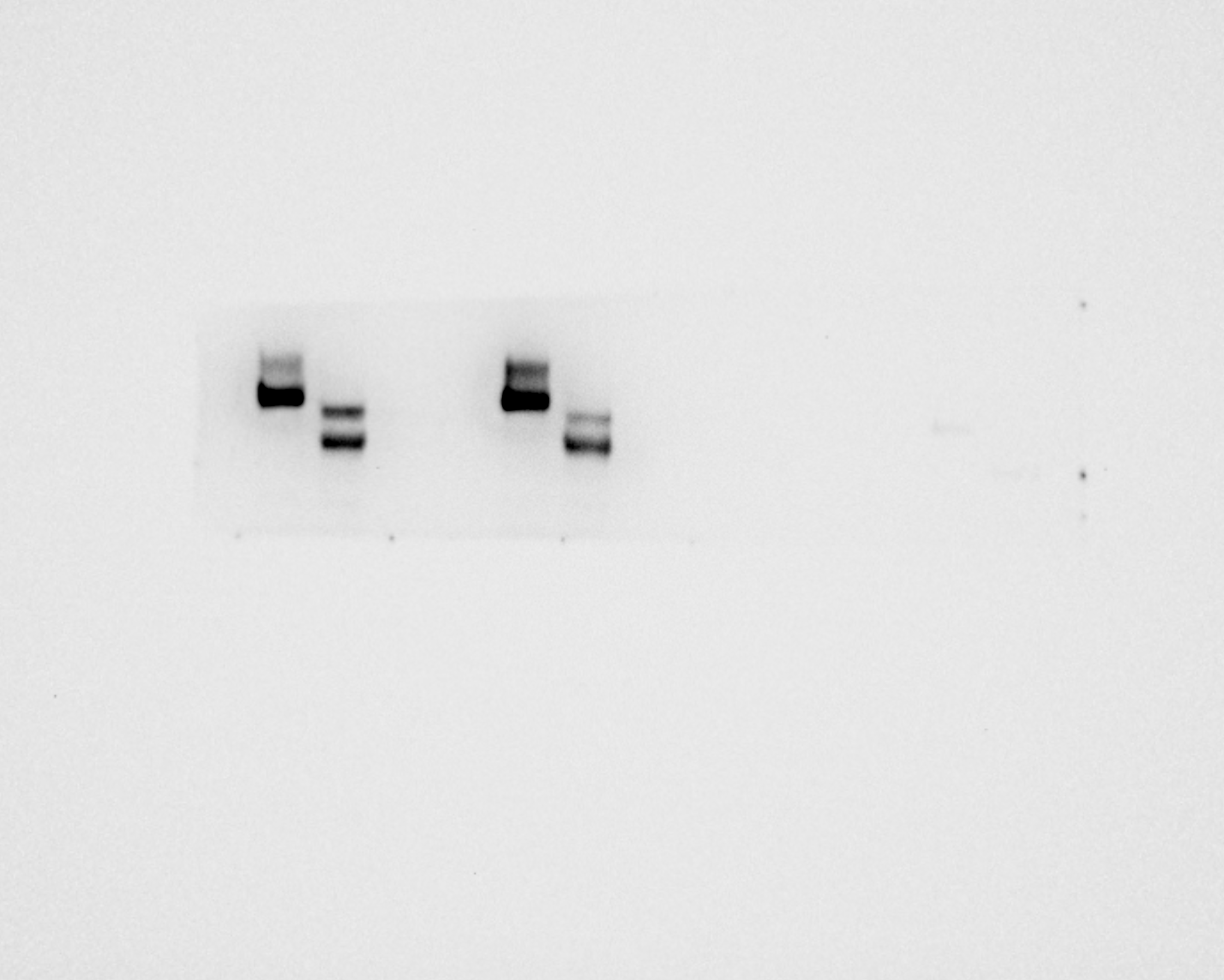

Supplement: Figure 3—source data 1. [file elife-69199-fig3-data1.zip › Figure 3A_Source Data/Figure_3A-Source_data_1_raw_ipFlag_anti_Flag_immunoblot.tif]

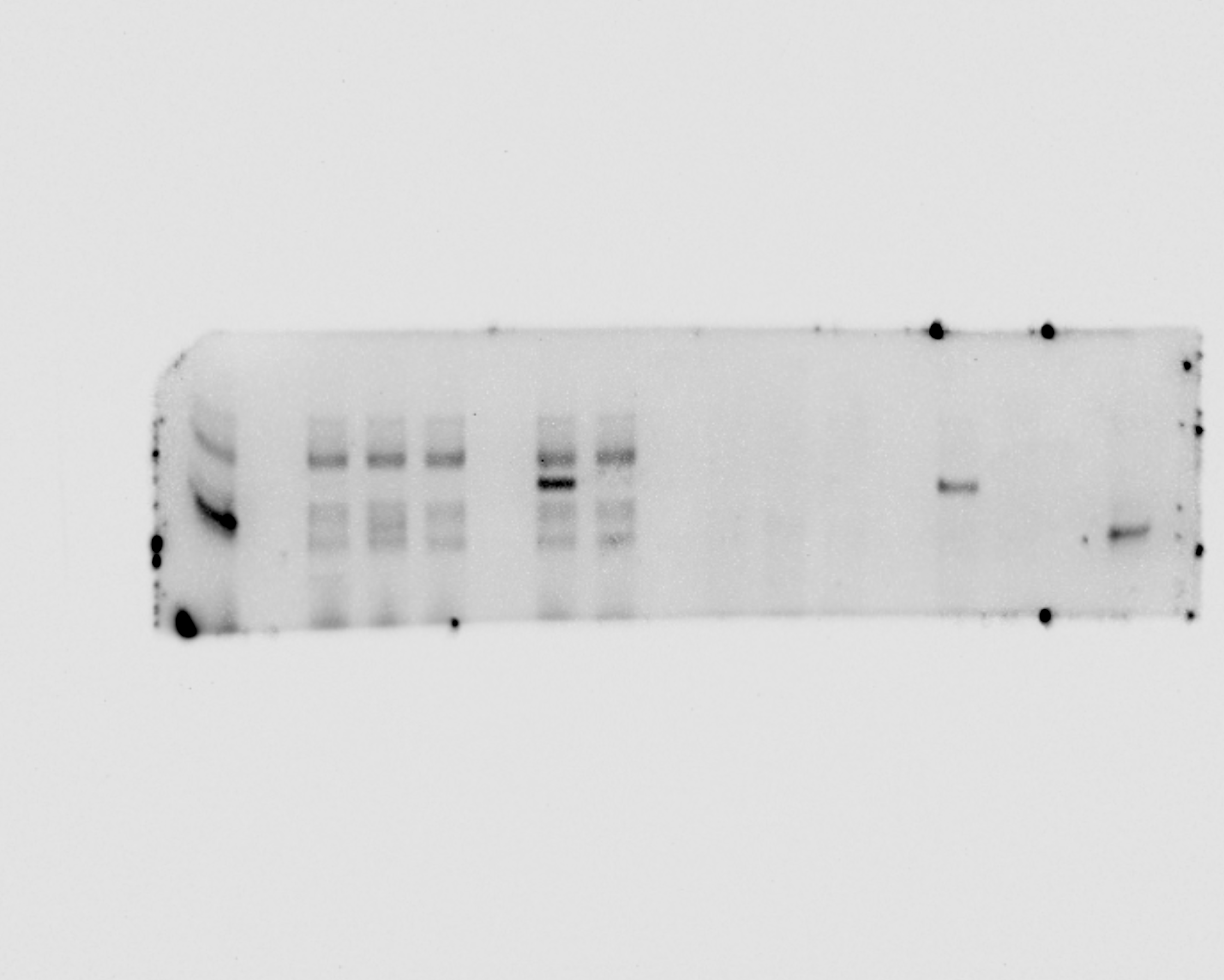

Supplement: Figure 3—source data 1. [file elife-69199-fig3-data1.zip › Figure 3A_Source Data/Figure_3A-Source_data_1_raw_ipMyc_anti_flag_immunoblot.tif]

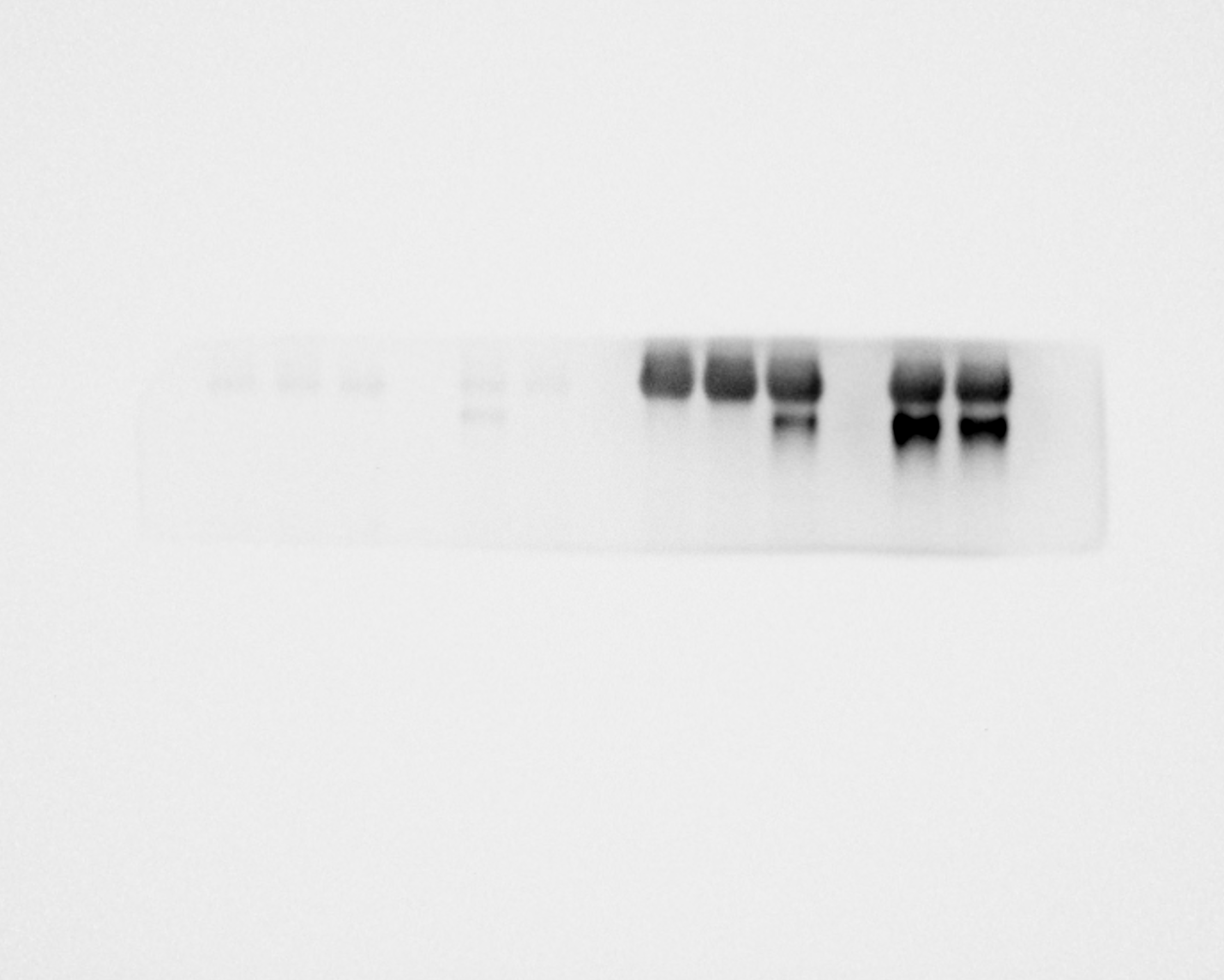

Supplement: Figure 3—source data 1. [file elife-69199-fig3-data1.zip › Figure 3A_Source Data/Figure_3A-Source_data_1_raw_ipMyc_anti_Myc_immunoblot.tif]

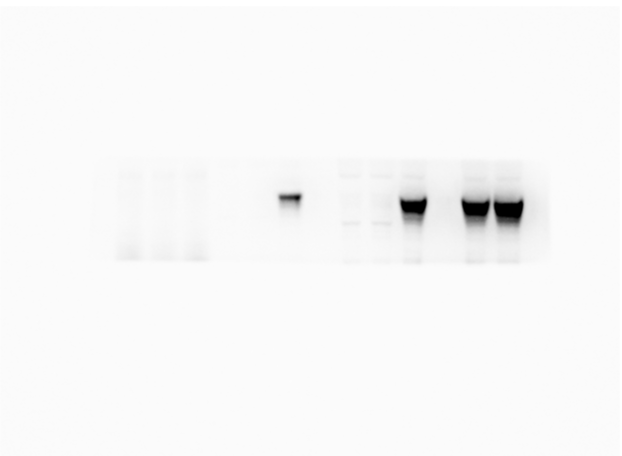

Supplement: Figure 3—source data 1. [file elife-69199-fig3-data1.zip › Figure 3A_Source Data/Figure_3A-Source_data_1_raw_input_anti_Myc_immunoblot.tif]

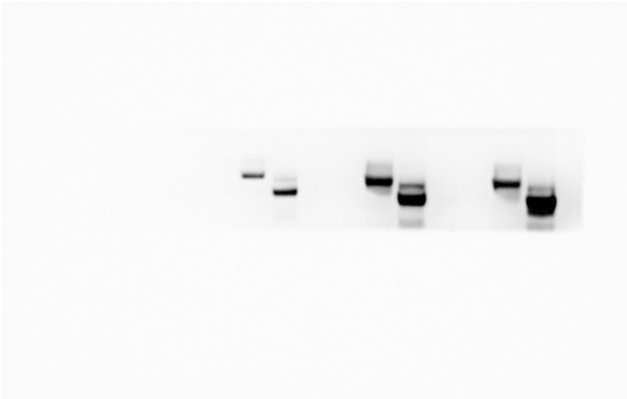

Supplement: Figure 3—source data 1. [file elife-69199-fig3-data1.zip › Figure 3A_Source Data/Figure_3A-Source_data_1_raw_input_anti_Flag_immunoblot.tif]

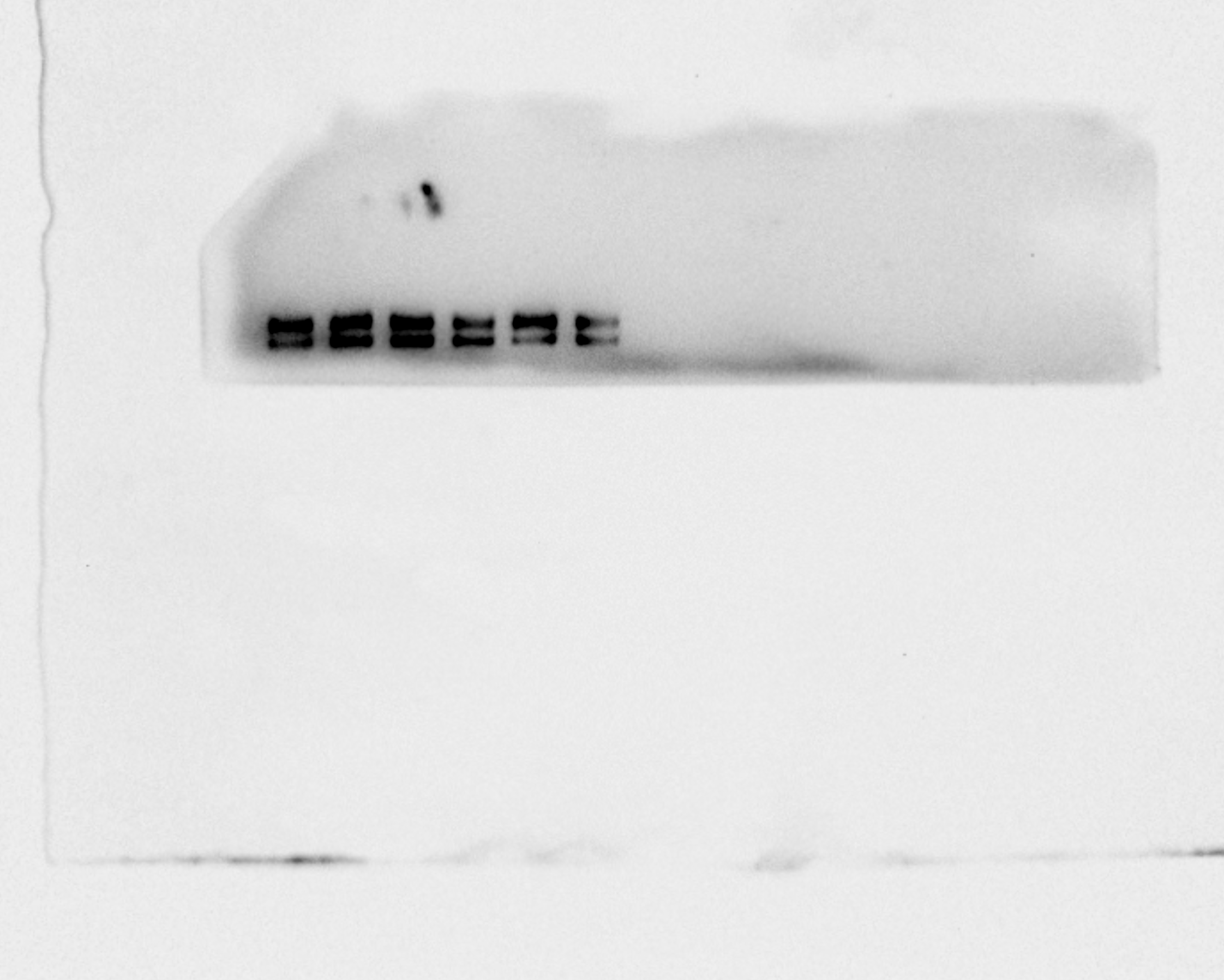

Supplement: Figure 3—source data 2. [file elife-69199-fig3-data2.zip › Figure 3F_Source Data/Figure_3F-Source_data_1_raw_WB_anti_APP_immunoblot.tif]

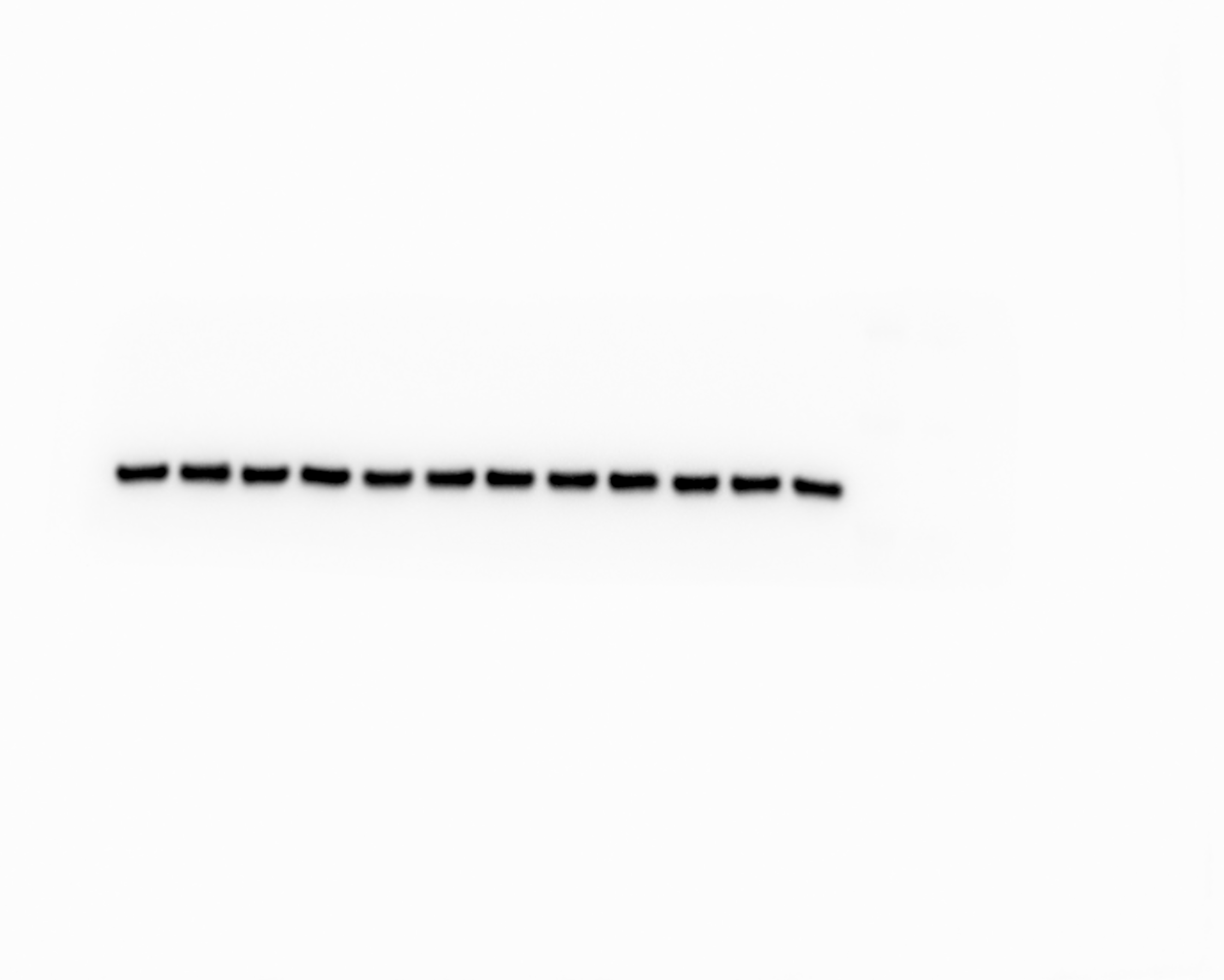

Supplement: Figure 3—source data 2. [file elife-69199-fig3-data2.zip › Figure 3F_Source Data/Figure_3F-Source_data_1_raw_WB_anti_Actin_immunoblot.tif]

Figure 3F-Source data

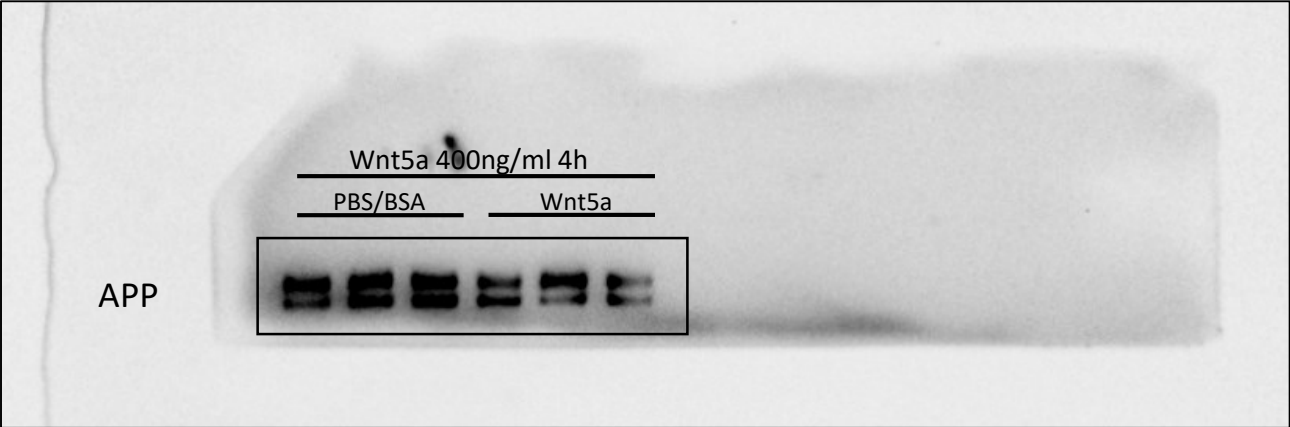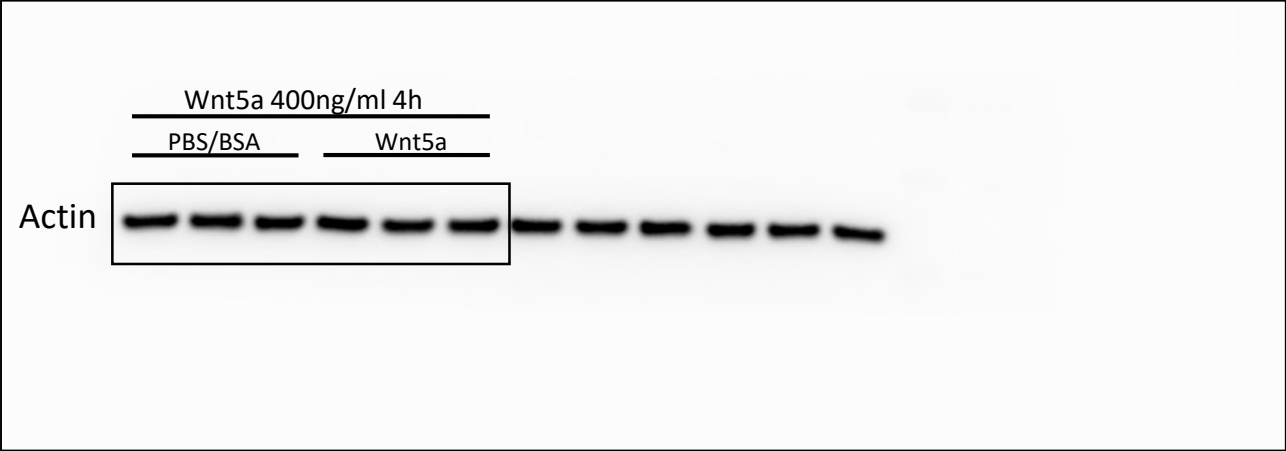

Supplement: Figure 3—source data 2. [file elife-69199-fig3-data2.zip › Figure 3F_Source Data/Figure 3F-Source data 1 labeled bands.pdf]

Figure 3I-Source data

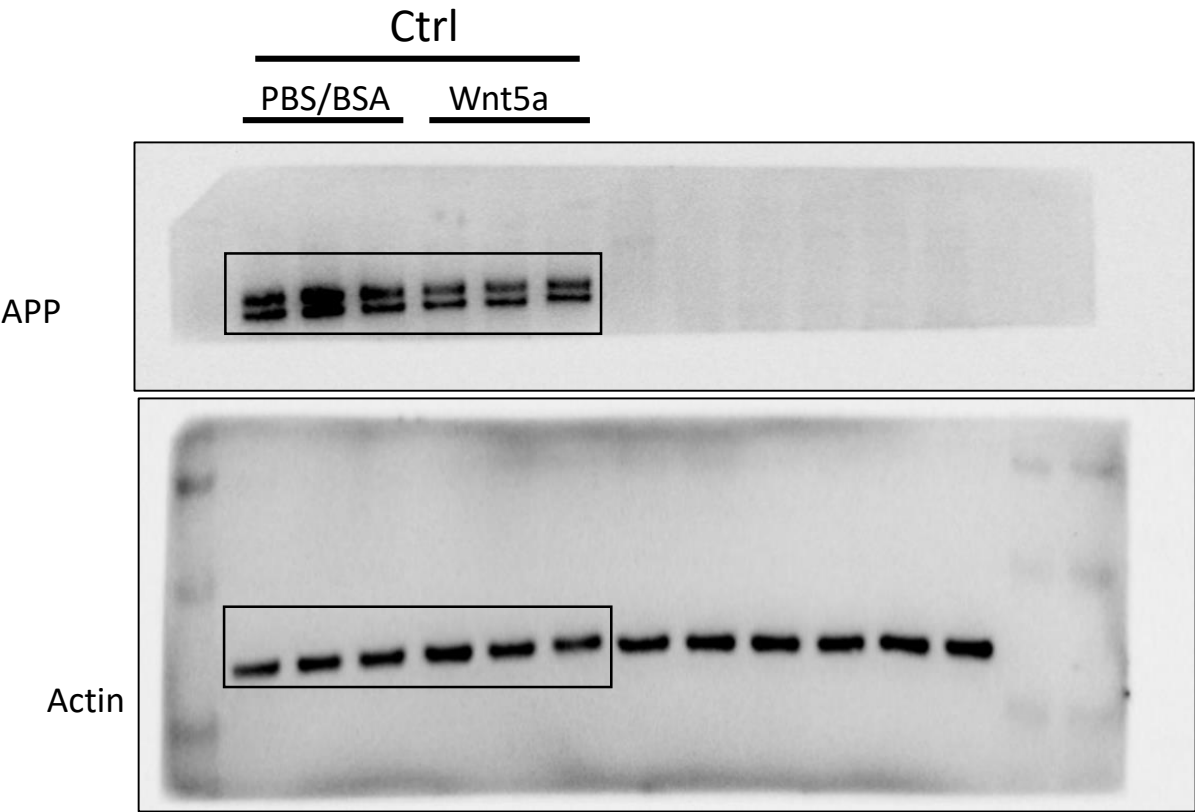

Supplement: Figure 3—source data 3. [file elife-69199-fig3-data3.zip › Figure 3I_Source Data/Figure 3I-Source data 1 labeled bands.pdf]

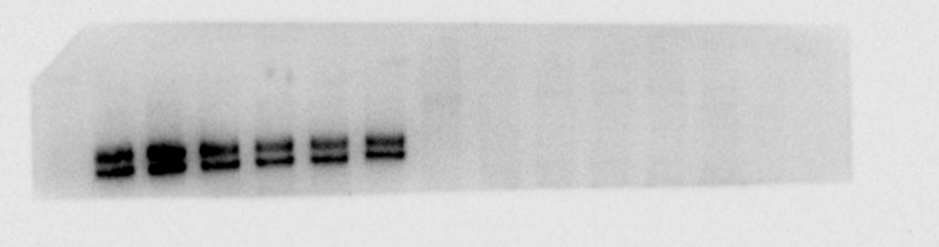

Supplement: Figure 3—source data 3. [file elife-69199-fig3-data3.zip › Figure 3I_Source Data/Figure_3I-Source_data_1_raw_WB_anti_APP_immunoblot.tif]

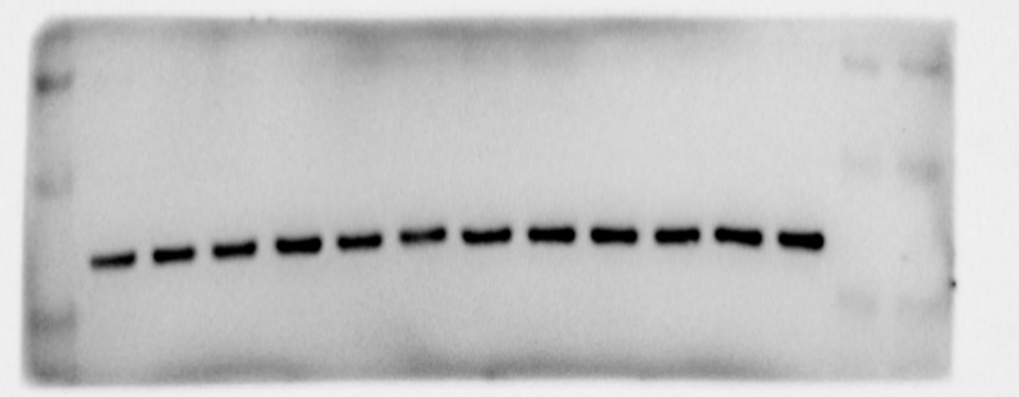

Supplement: Figure 3—source data 3. [file elife-69199-fig3-data3.zip › Figure 3I_Source Data/Figure_3I-Source_data_1_raw_WB_anti_Actin_immunoblot.tif]

Figure 3J-Source data

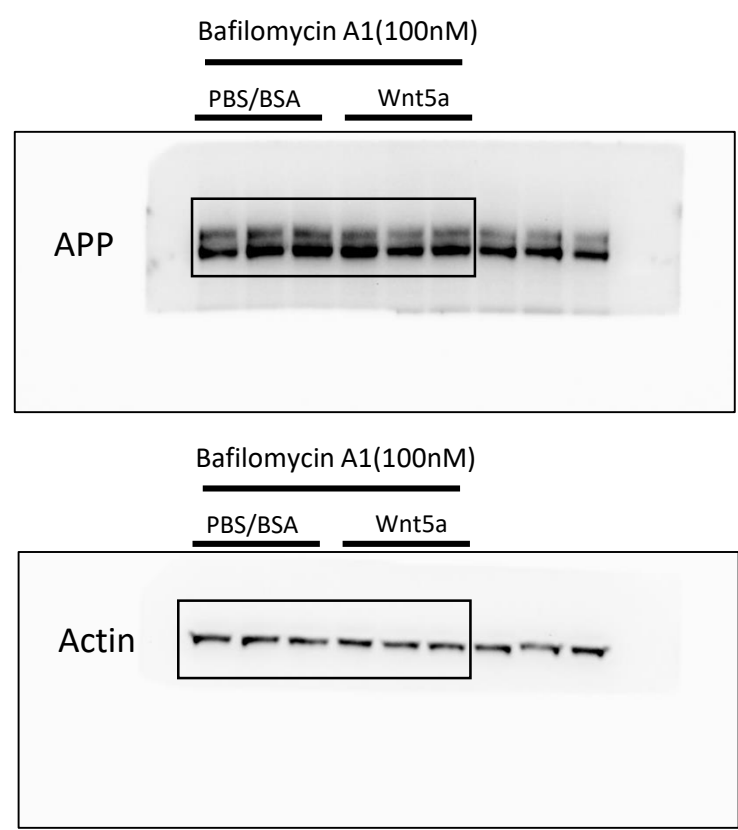

Supplement: Figure 3—source data 4. [file elife-69199-fig3-data4.zip › Figure 3J_Source Data/Figure 3J-Source data 1 labeled bands.pdf]

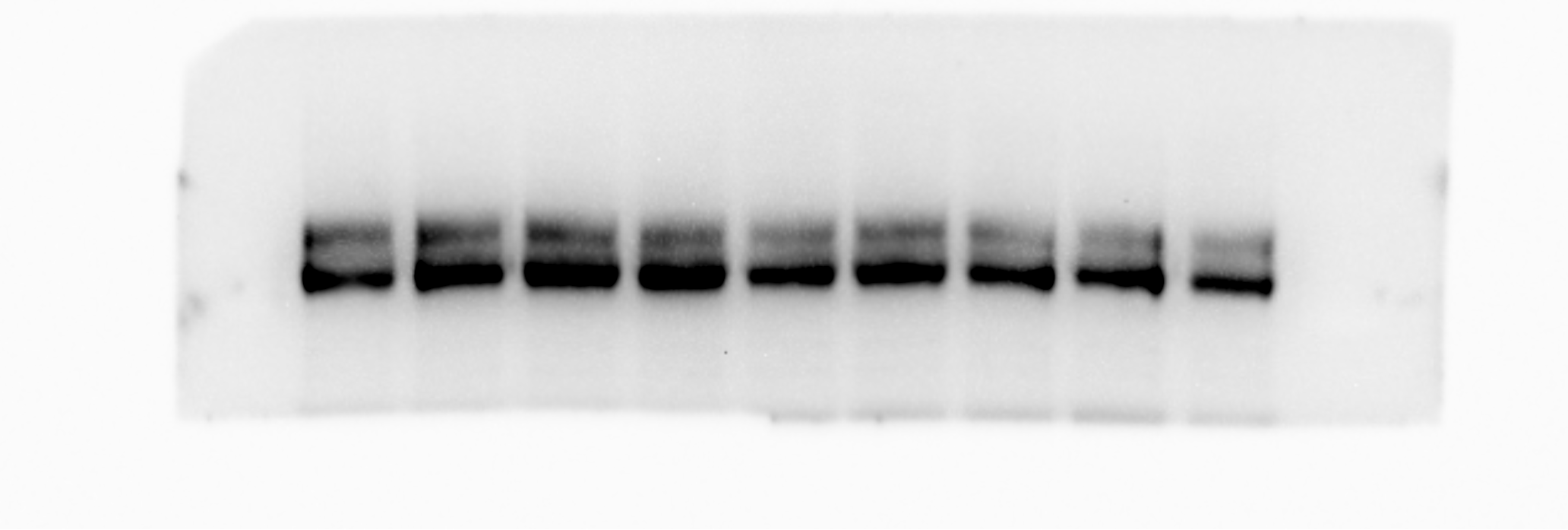

Supplement: Figure 3—source data 4. [file elife-69199-fig3-data4.zip › Figure 3J_Source Data/Figure_3J-Source_data_1_raw_WB_anti_APP_immunoblot.tif]

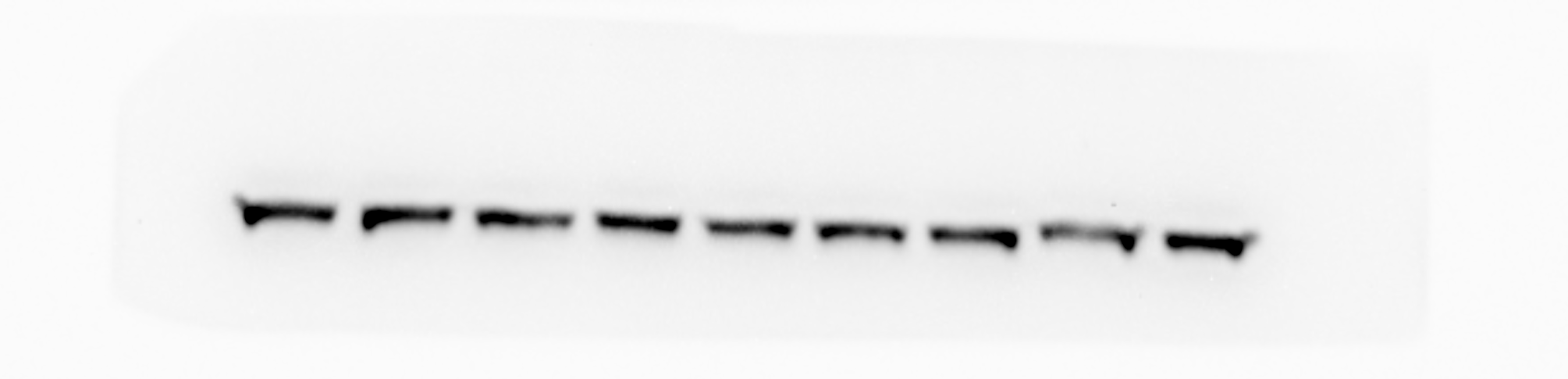

Supplement: Figure 3—source data 4. [file elife-69199-fig3-data4.zip › Figure 3J_Source Data/Figure_3J-Source_data_1_raw_WB_anti_Actin_immunoblot.tif]

Figure S3A-Source data

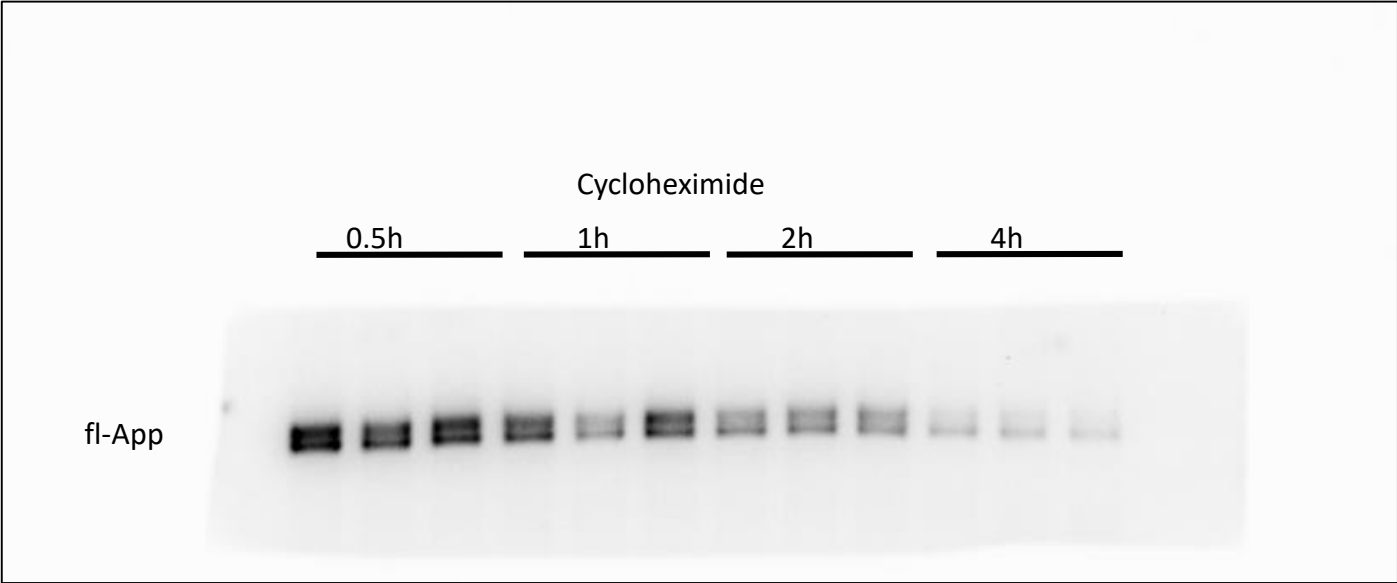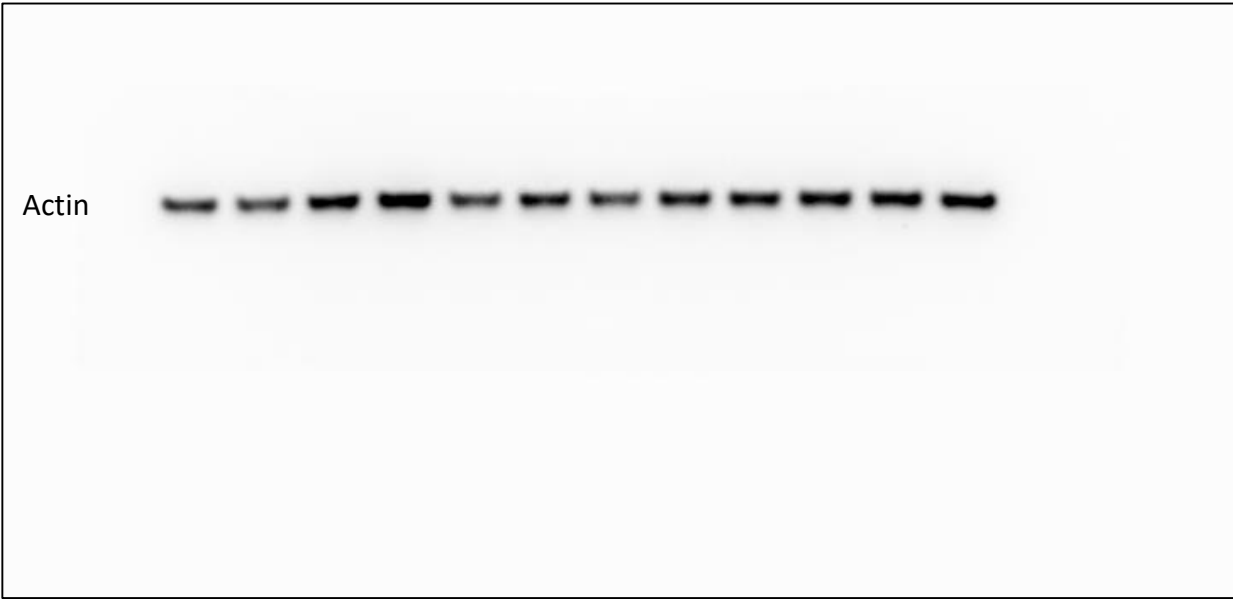

Supplement: Figure 3—figure supplement 1—source data 1. [file elife-69199-fig3-figsupp1-data1.zip › Figure 3- supplement 1A_Source Data/Figure 3- supplement 1A-Source data 1 labeled bands.pdf]

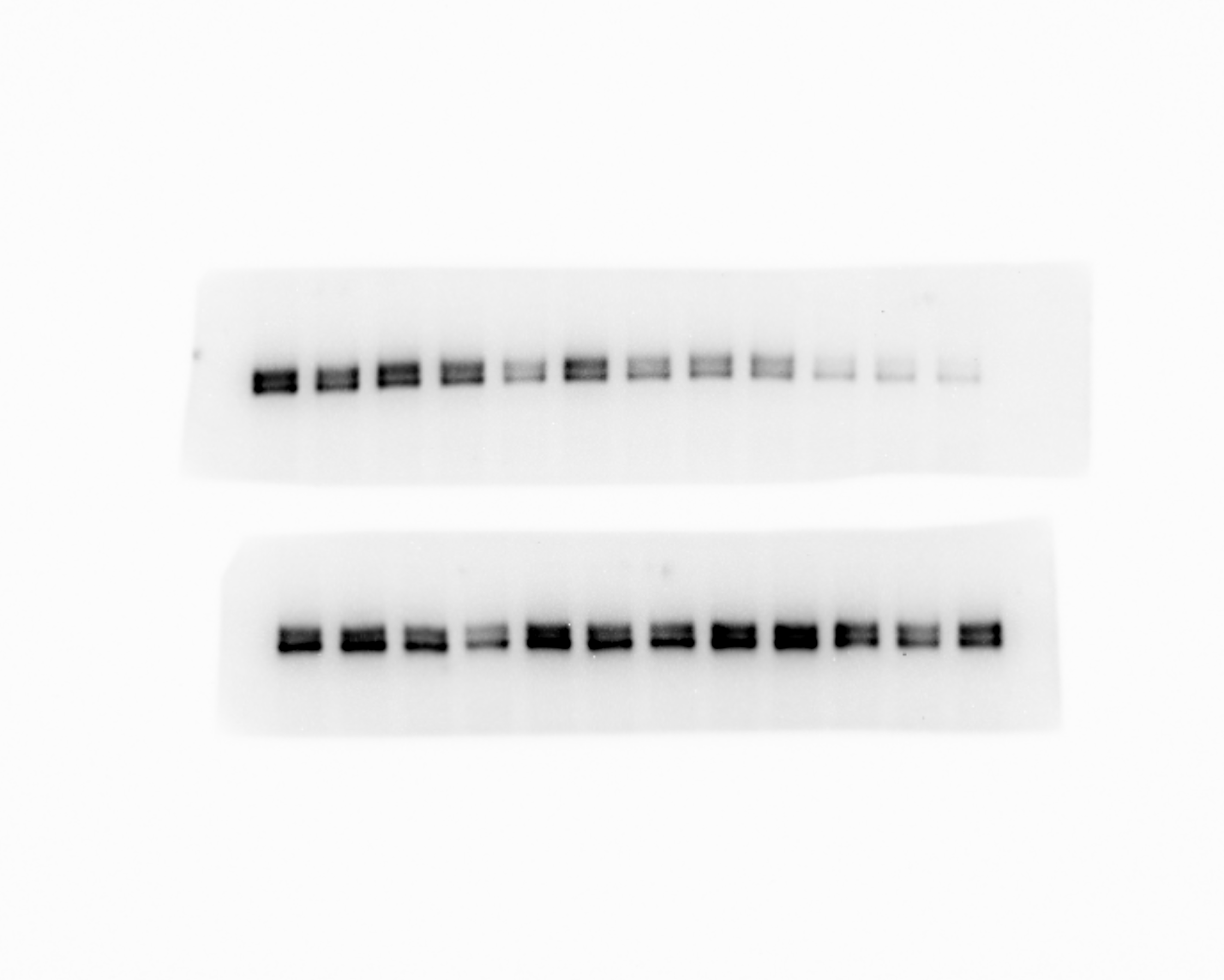

Supplement: Figure 3—figure supplement 1—source data 1. [file elife-69199-fig3-figsupp1-data1.zip › Figure 3- supplement 1A_Source Data/Figure 3- supplement 1A-Source_data_1_raw_WB_anti_APP_immunoblot.tif]

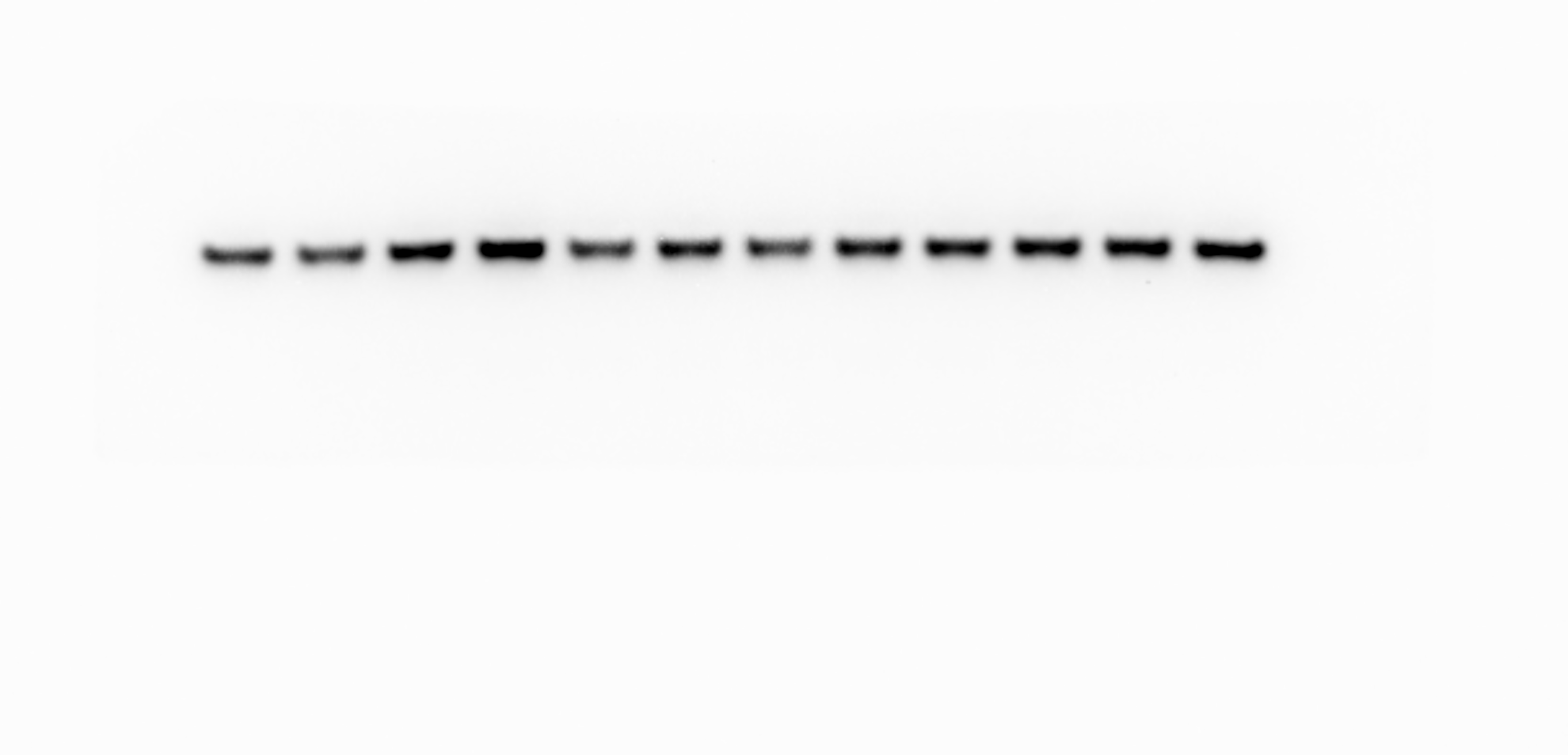

Supplement: Figure 3—figure supplement 1—source data 1. [file elife-69199-fig3-figsupp1-data1.zip › Figure 3- supplement 1A_Source Data/Figure 3- supplement 1A-Source_data_1_raw_WB_anti_Actin_immunoblot.tif]

Figure S3B-Source data

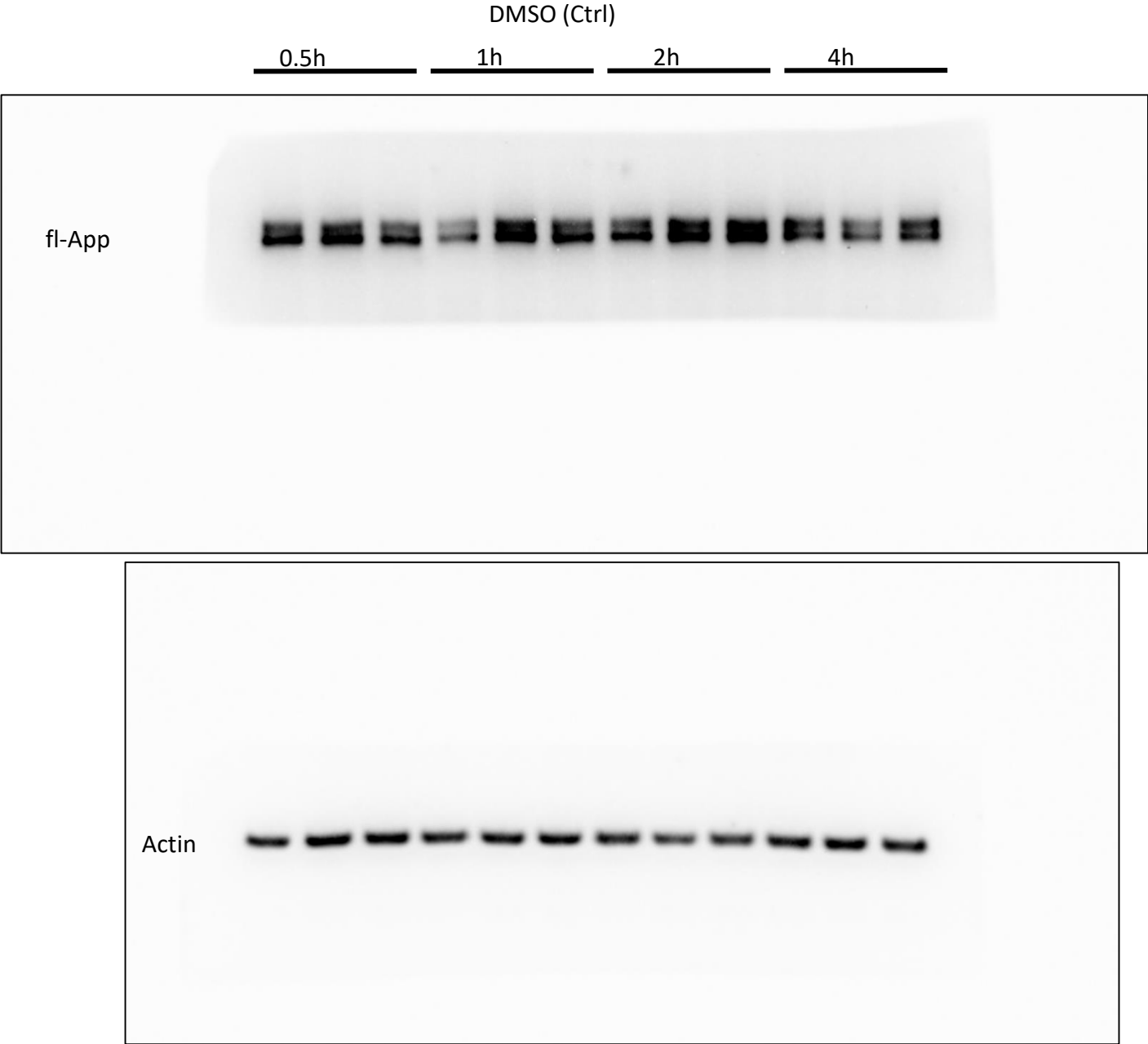

Supplement: Figure 3—figure supplement 1—source data 2. [file elife-69199-fig3-figsupp1-data2.zip › Figure 3- supplement 1B_Source Data/Figure 3- supplement 1B-Source data 1 labeled bands.pdf]

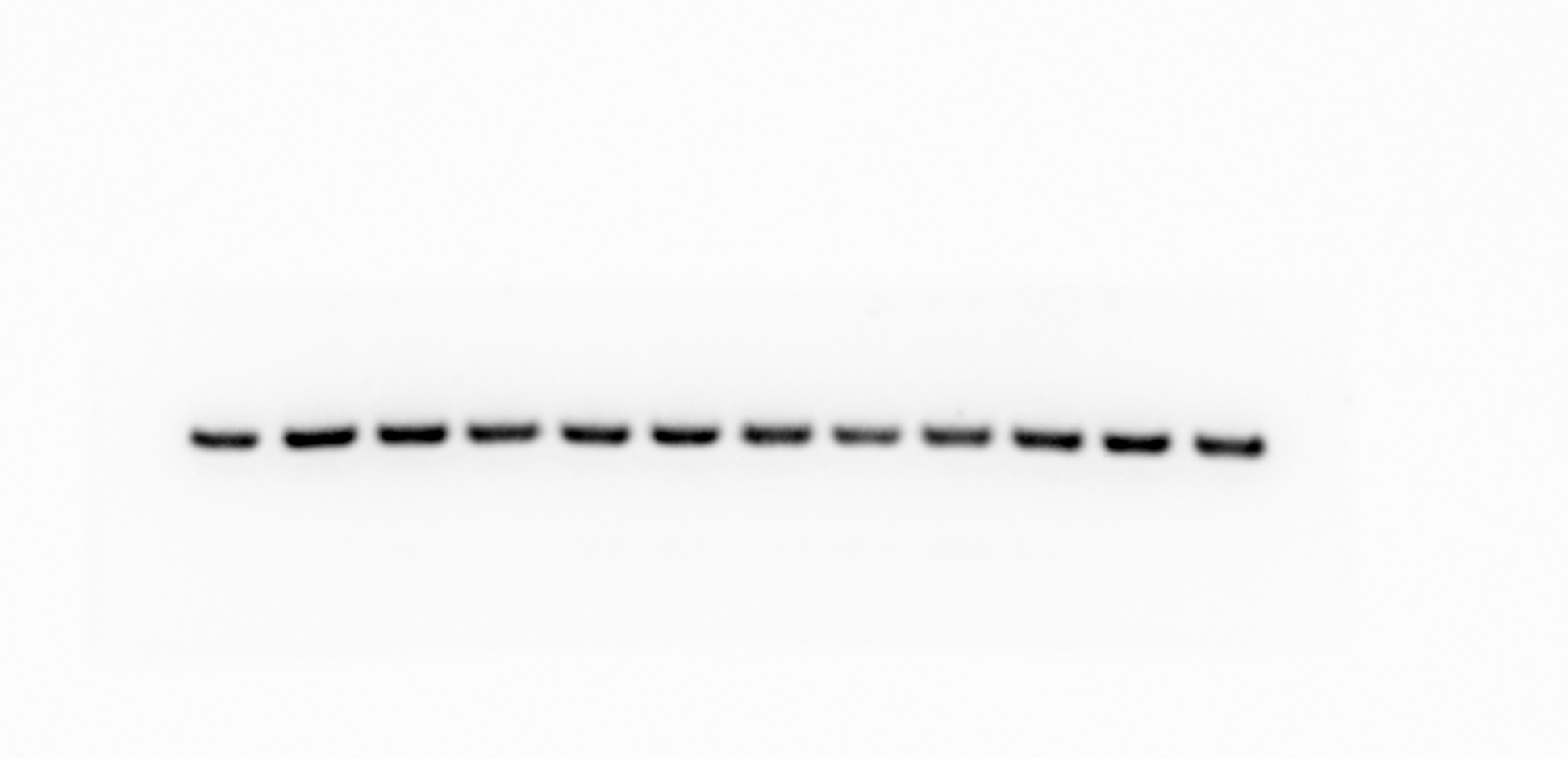

Supplement: Figure 3—figure supplement 1—source data 2. [file elife-69199-fig3-figsupp1-data2.zip › Figure 3- supplement 1B_Source Data/Figure 3- supplement 1B-Source_data_1_raw_WB_anti_Actin_immunoblot.tif]

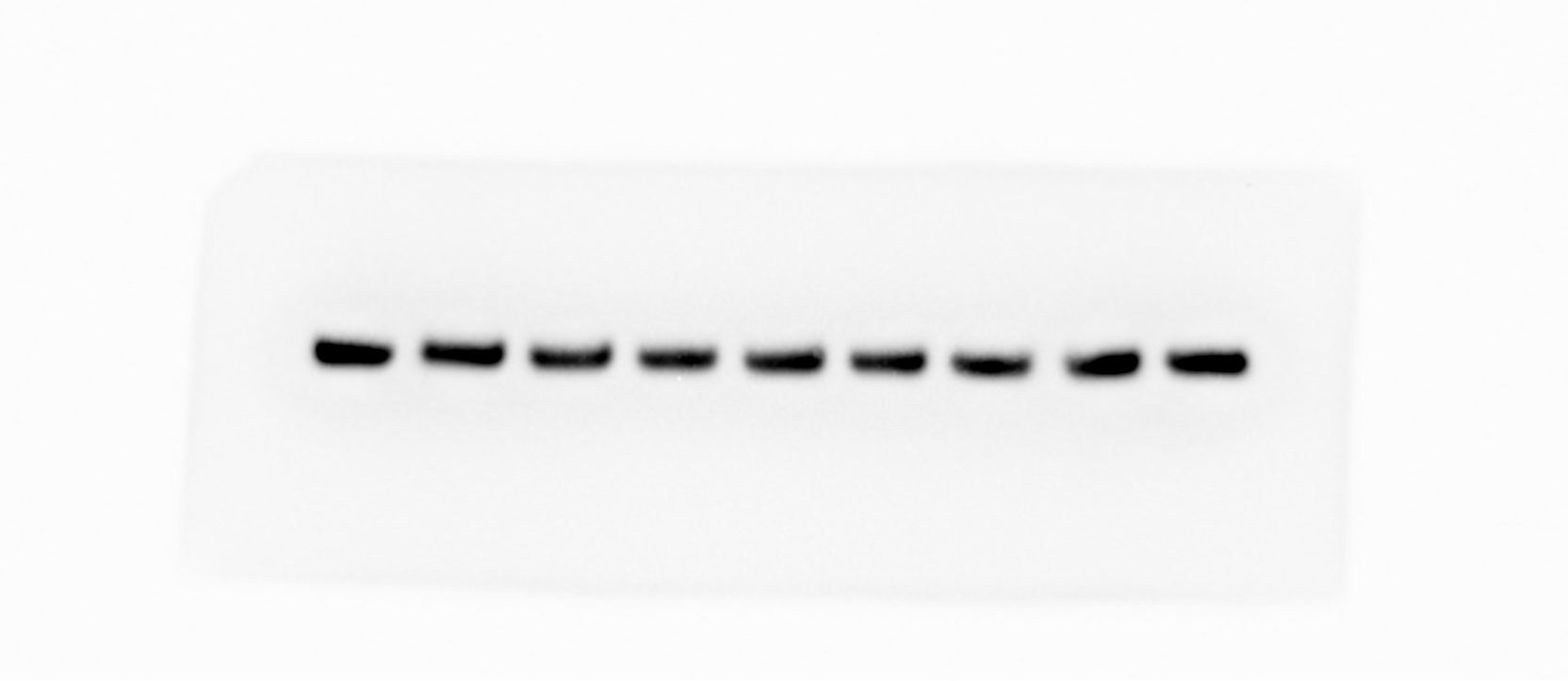

Supplement: Figure 3—figure supplement 4—source data 1. [file elife-69199-fig3-figsupp4-data1.zip › Figure 3-figure supplement 4/Figure 3-figure supplement 4-Source_data_1_raw_WB_anti_Actin_immunoblot.tif]

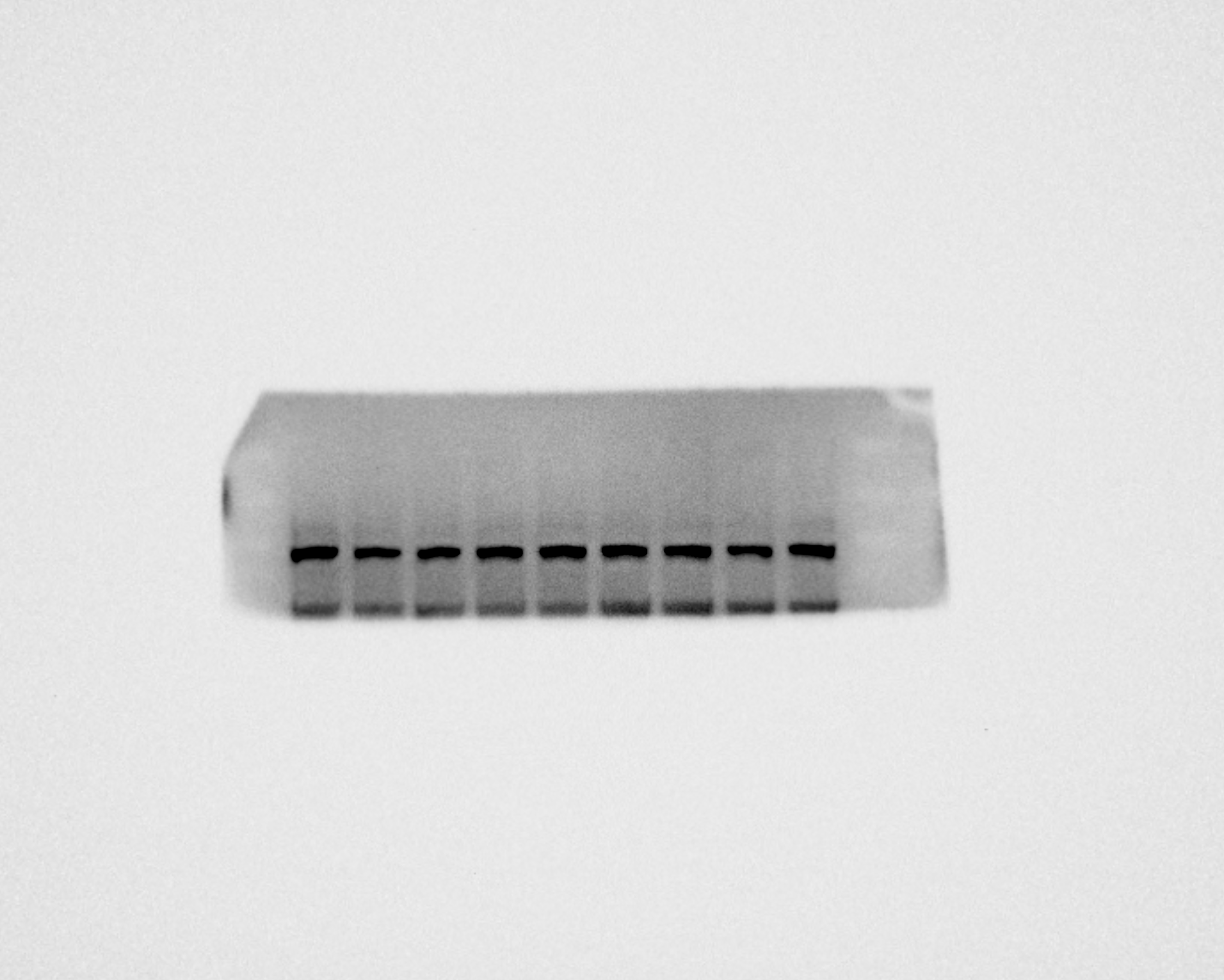

Supplement: Figure 3—figure supplement 4—source data 1. [file elife-69199-fig3-figsupp4-data1.zip › Figure 3-figure supplement 4/Figure 3-figure supplement 4-Source_data_1_raw_WB_anti_Golgin97_immunoblot.tif]

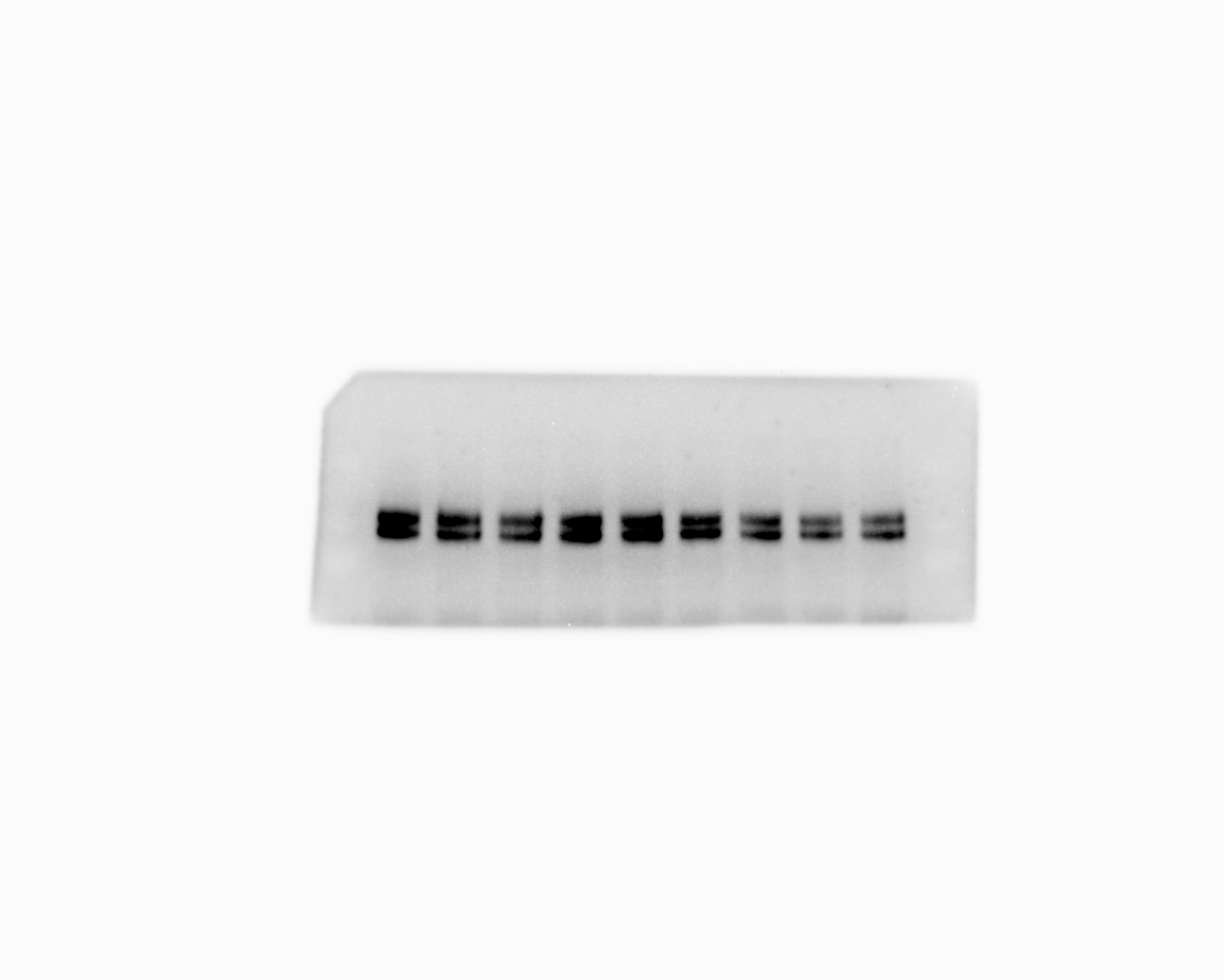

Supplement: Figure 3—figure supplement 4—source data 1. [file elife-69199-fig3-figsupp4-data1.zip › Figure 3-figure supplement 4/Figure 3-figure supplement 4-Source_data_1_raw_WB_anti_APP_immunoblot.tif]

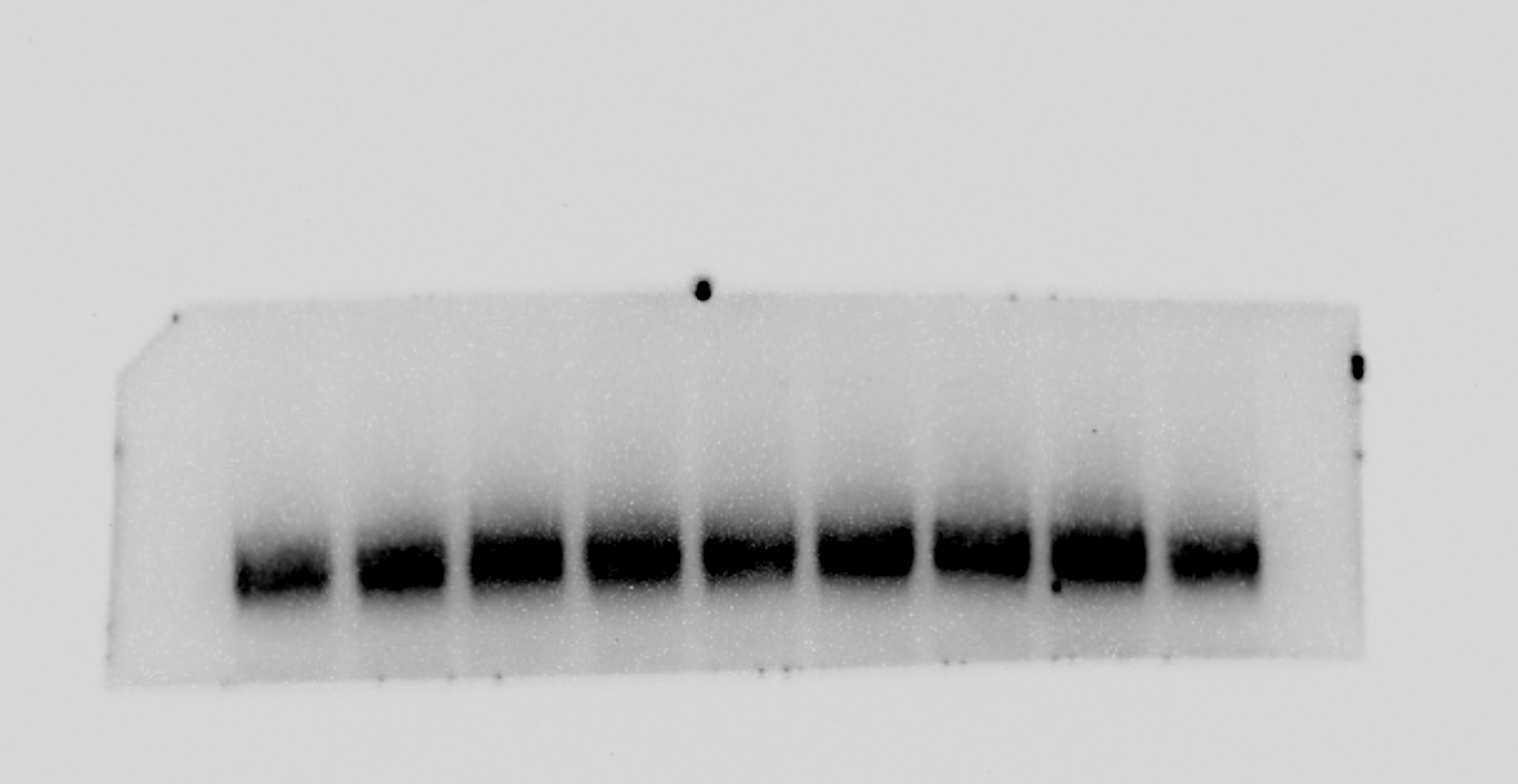

Supplement: Figure 3—figure supplement 4—source data 1. [file elife-69199-fig3-figsupp4-data1.zip › Figure 3-figure supplement 4/Figure 3-figure supplement 4-Source_data_1_raw_WB_anti_Lamp1_immunoblot.tif]

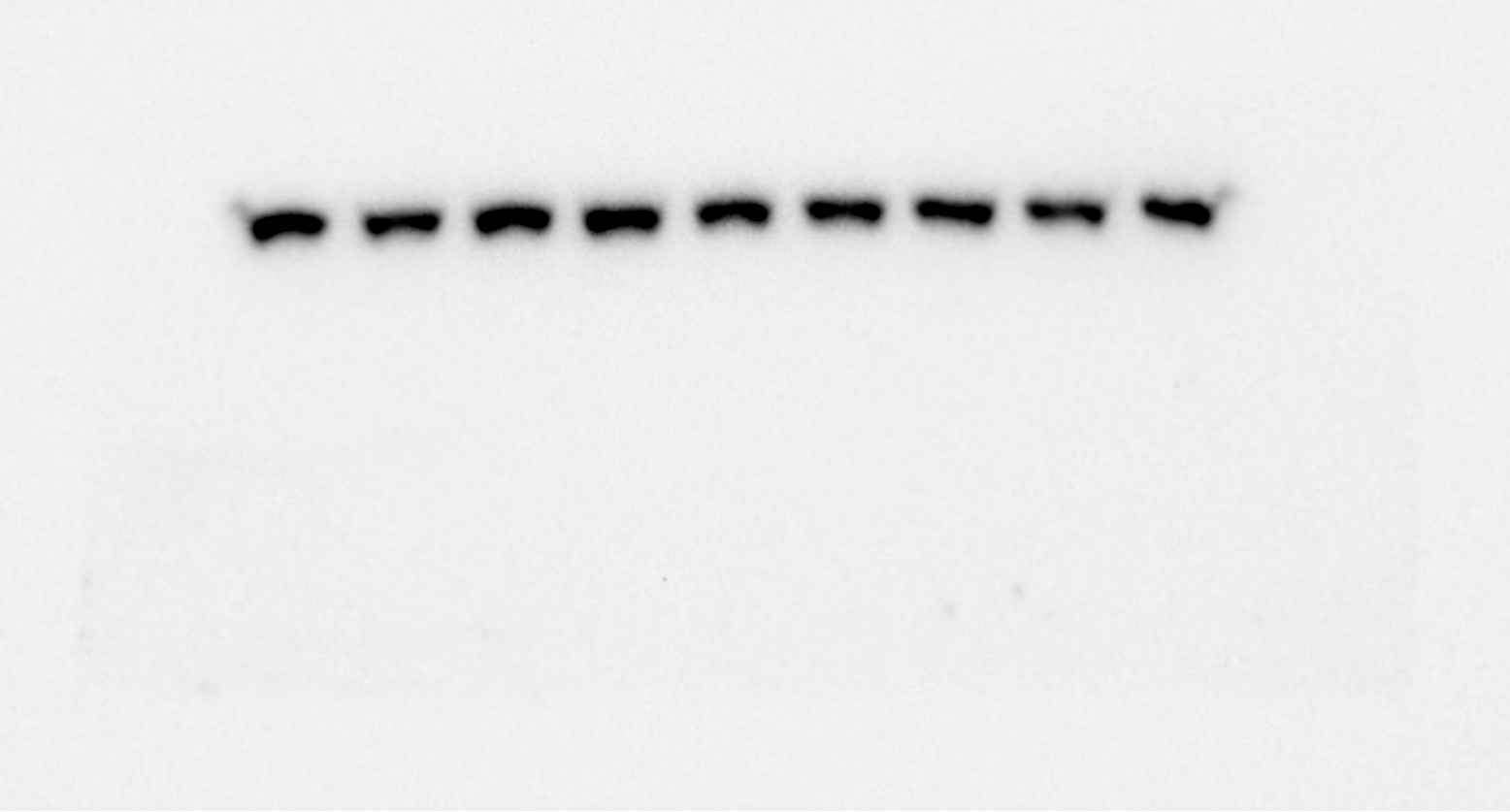

Supplement: Figure 3—figure supplement 4—source data 1. [file elife-69199-fig3-figsupp4-data1.zip › Figure 3-figure supplement 4/Figure 3-figure supplement 4-Source_data_1_raw_WB_anti_Rab5_immunoblot.tif]

Figure S14-Source data

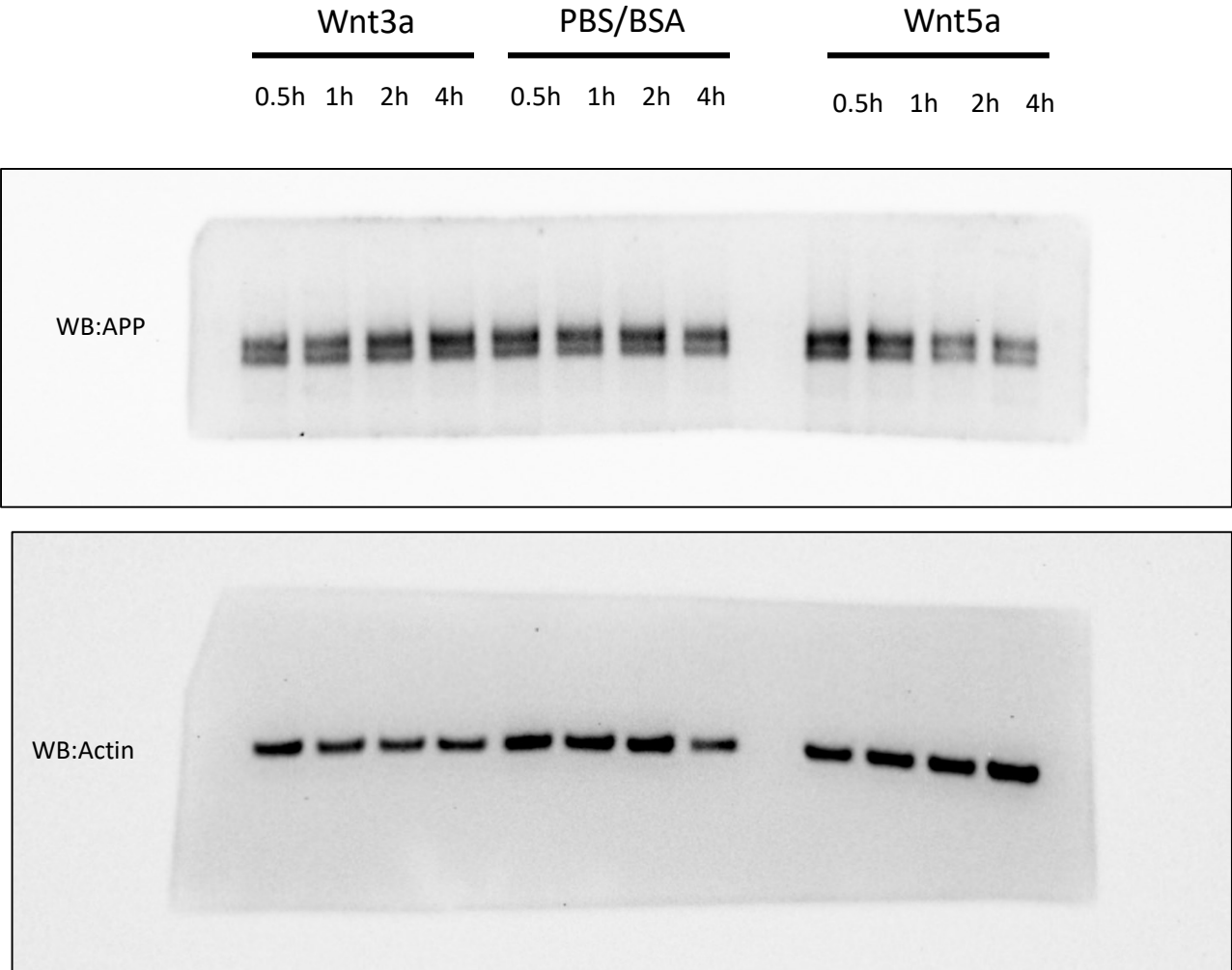

Supplement: Figure 3—figure supplement 5—source data 1. [file elife-69199-fig3-figsupp5-data1.zip › Figure 3-figure supplement 5-Source data 1 labeled bands.pdf]

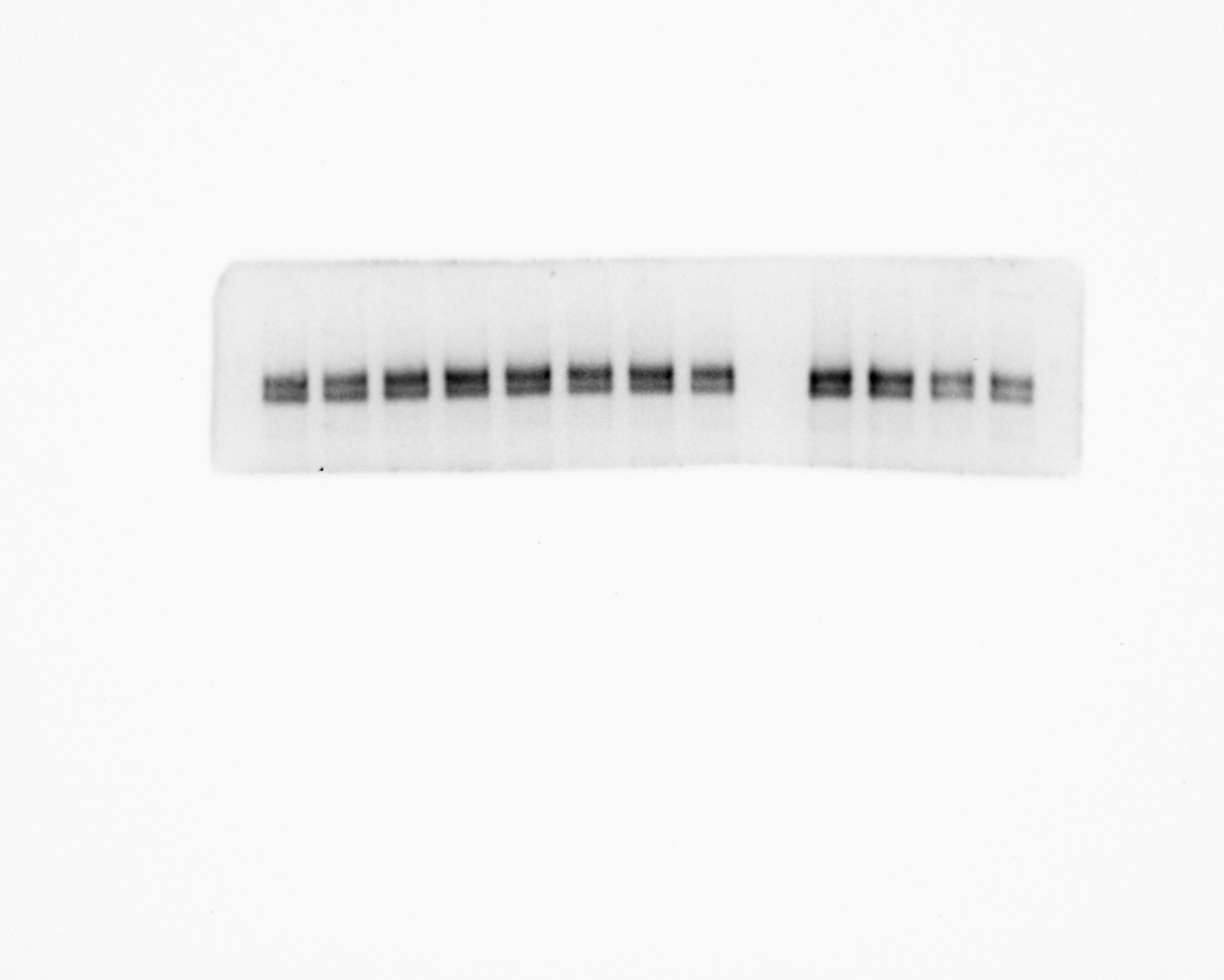

Supplement: Figure 3—figure supplement 5—source data 1. [file elife-69199-fig3-figsupp5-data1.zip › Figure 3-figure supplement 5-Source_data_1_raw_WB_anti_APP.tif]

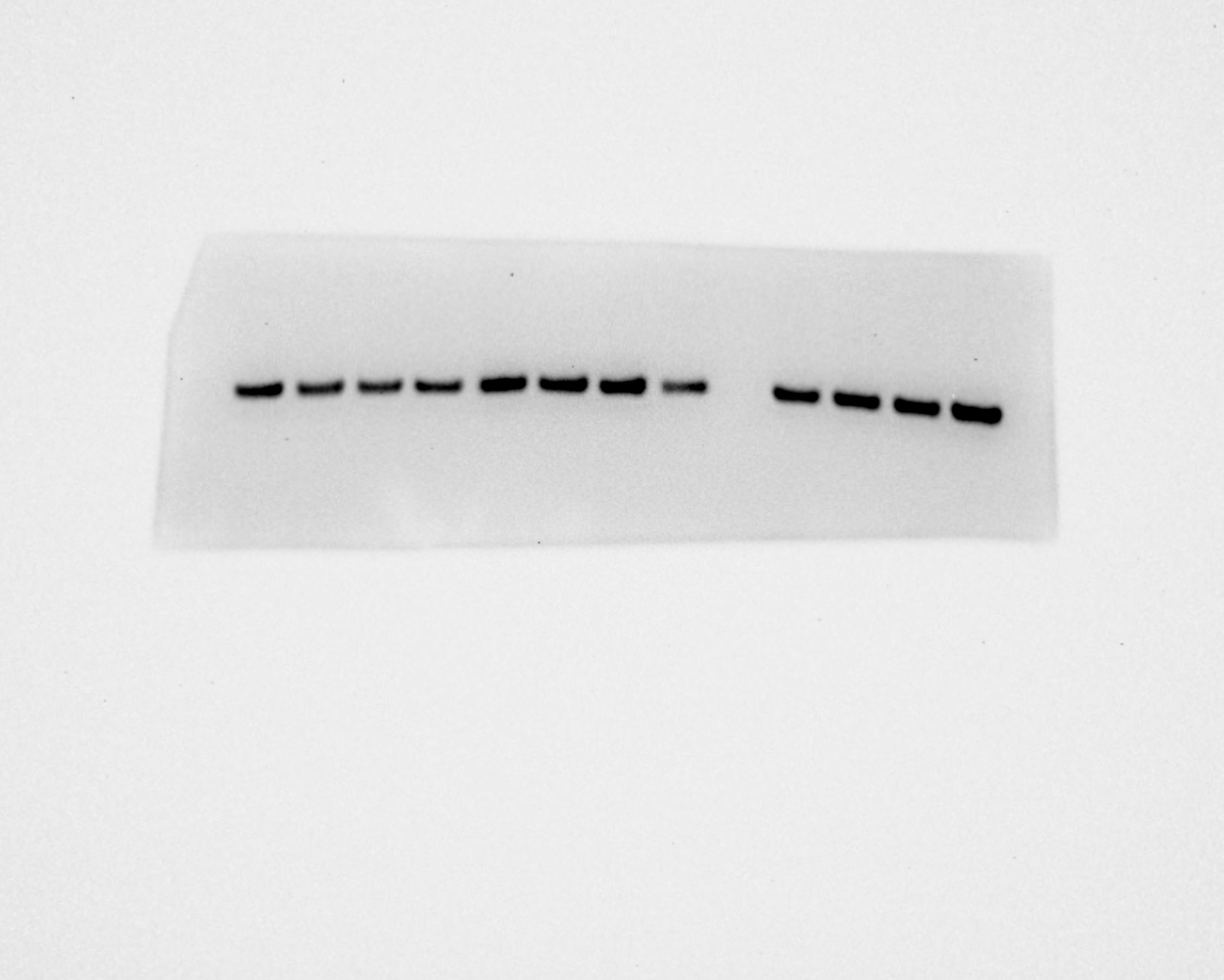

Supplement: Figure 3—figure supplement 5—source data 1. [file elife-69199-fig3-figsupp5-data1.zip › Figure 3-figure supplement 5-Source_data_1_raw_WB_anti_Actin.tif]

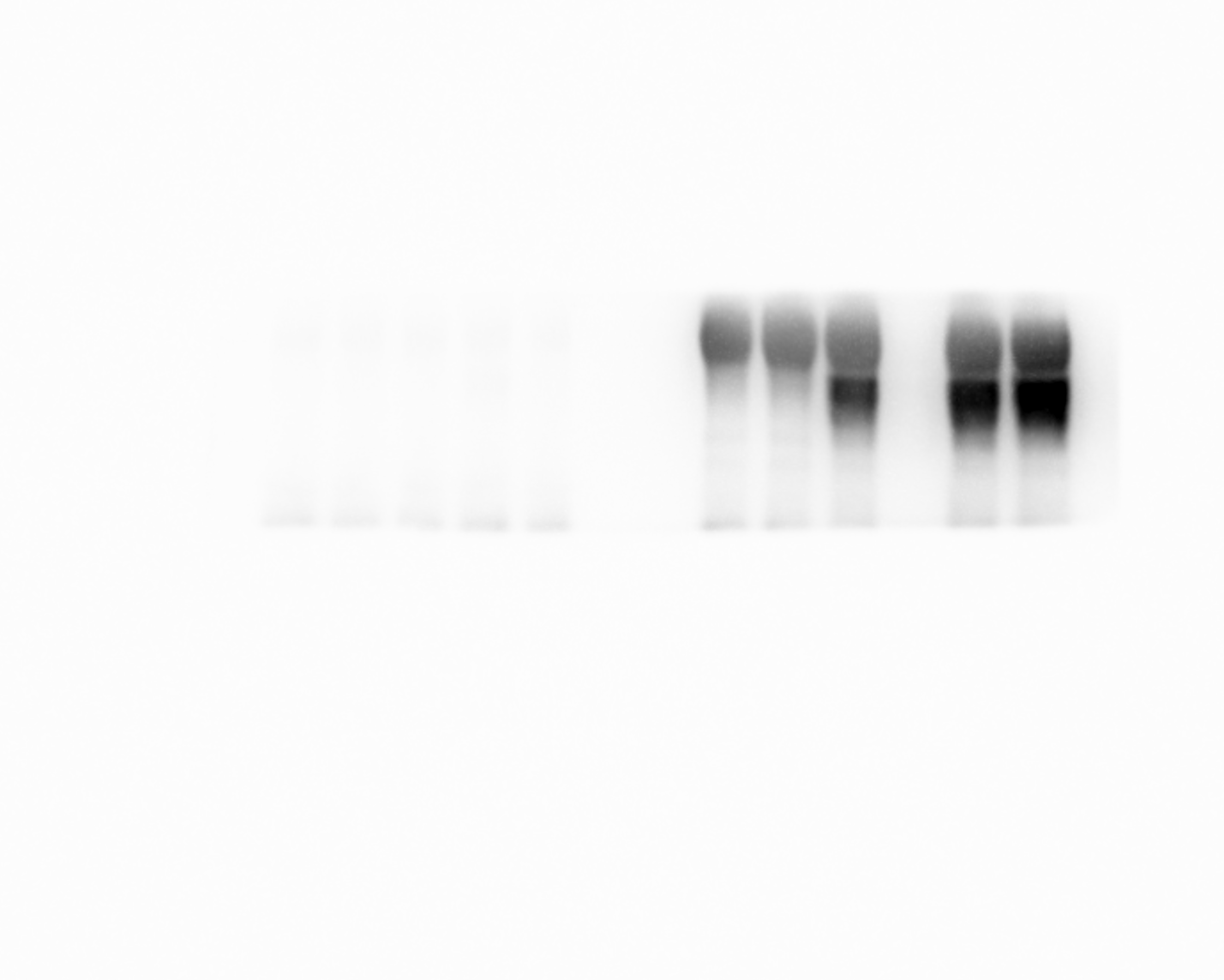

Supplement: Figure 4—source data 1. [file elife-69199-fig4-data1.zip › Figure 4A_Source Data/Figure_4A-Source_data_1_raw_ipV5_anti_V5_immunoblot.tif]

Figure 4A-Source data

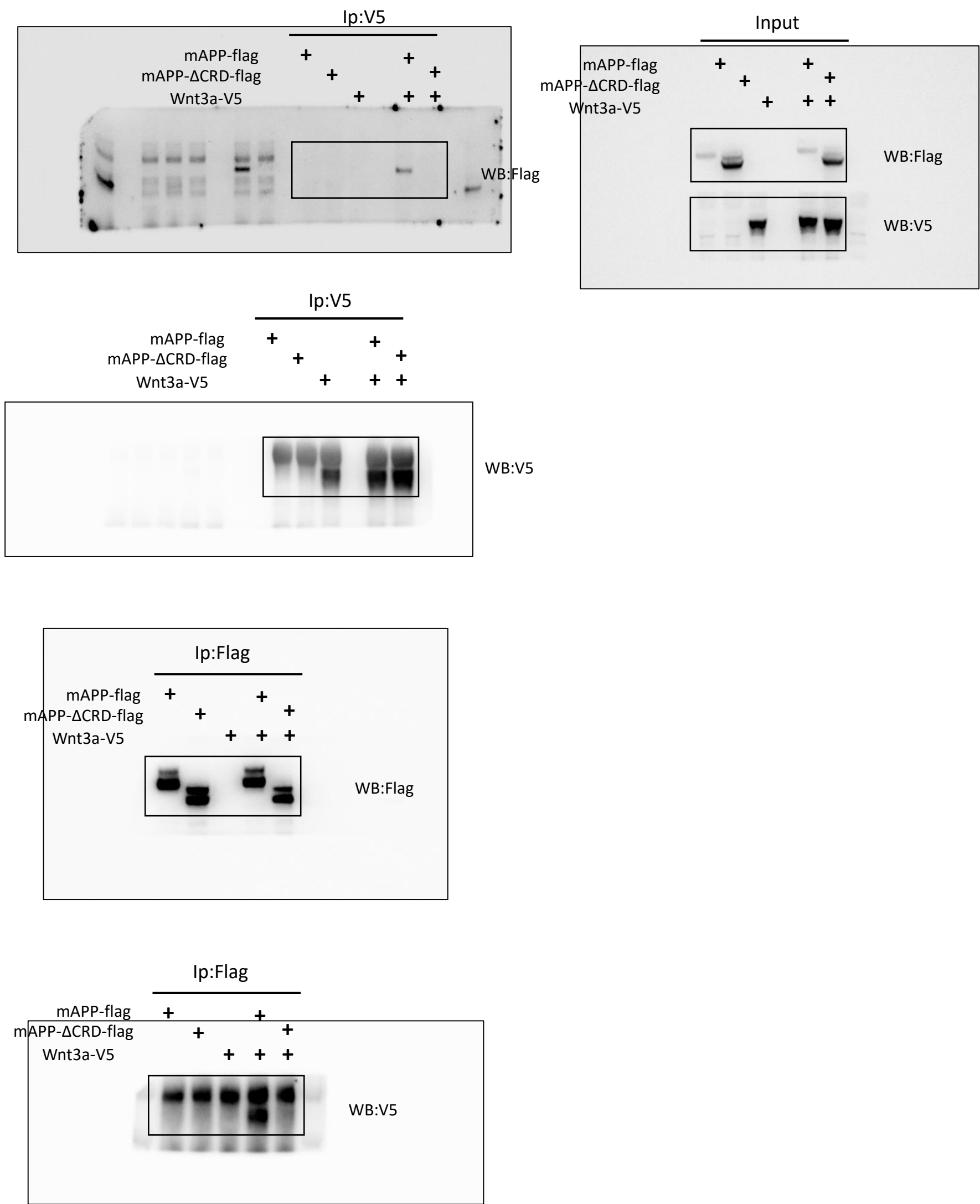

Supplement: Figure 4—source data 1. [file elife-69199-fig4-data1.zip › Figure 4A_Source Data/Figure 4A-Source data 1 labeled bands.pdf]

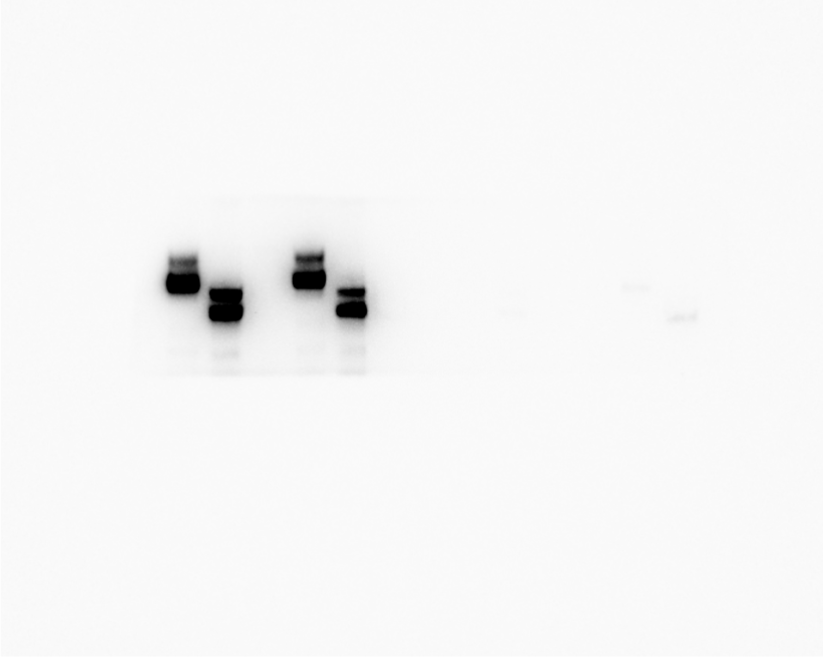

Supplement: Figure 4—source data 1. [file elife-69199-fig4-data1.zip › Figure 4A_Source Data/Figure_4A-Source_data_1_raw_ipFlag_anti_flag_immunoblot.tif]

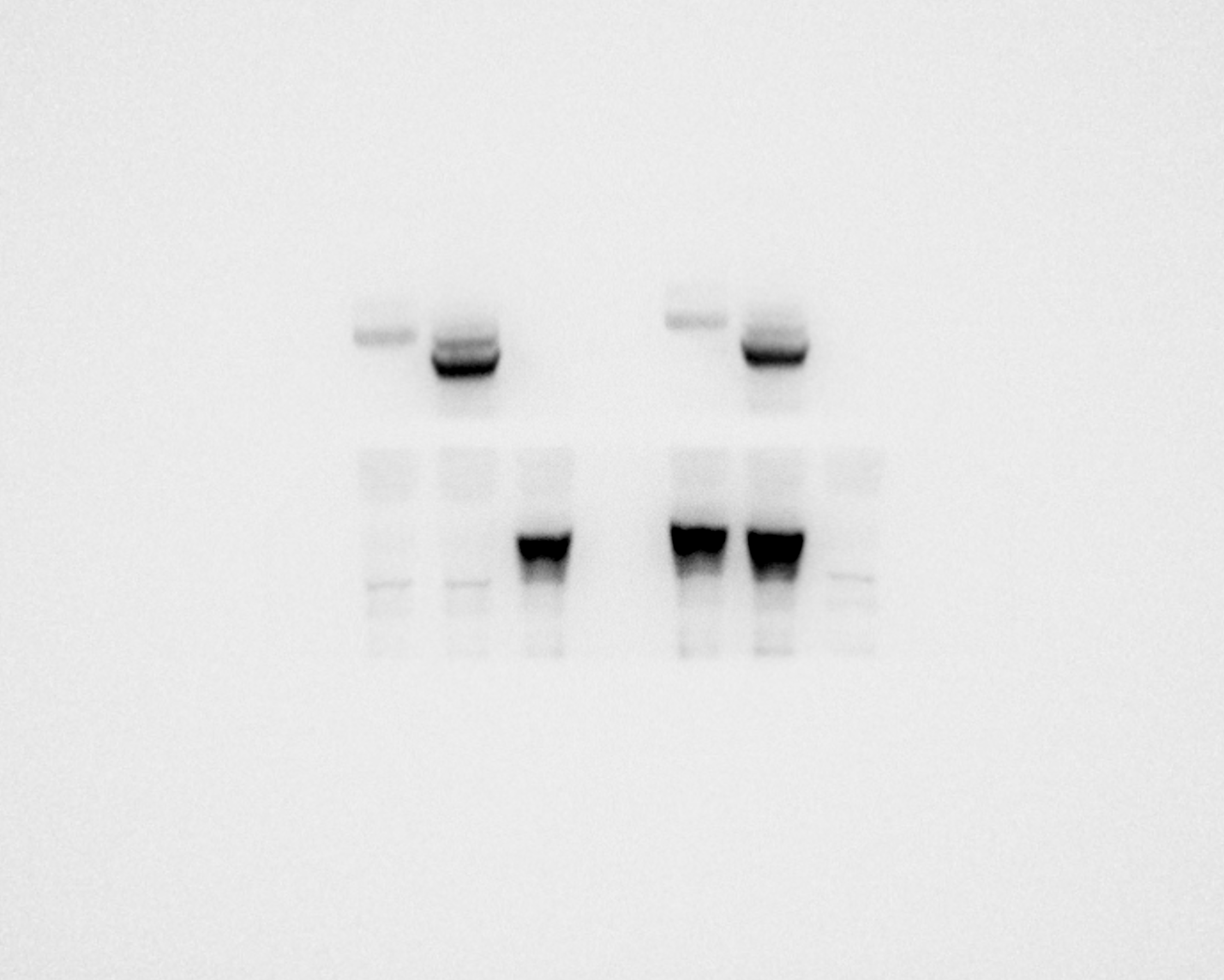

Supplement: Figure 4—source data 1. [file elife-69199-fig4-data1.zip › Figure 4A_Source Data/Figure_3A-Source_data_1_raw_input_anti_Flagup_and_anti_V5_down_immunoblot.tif]

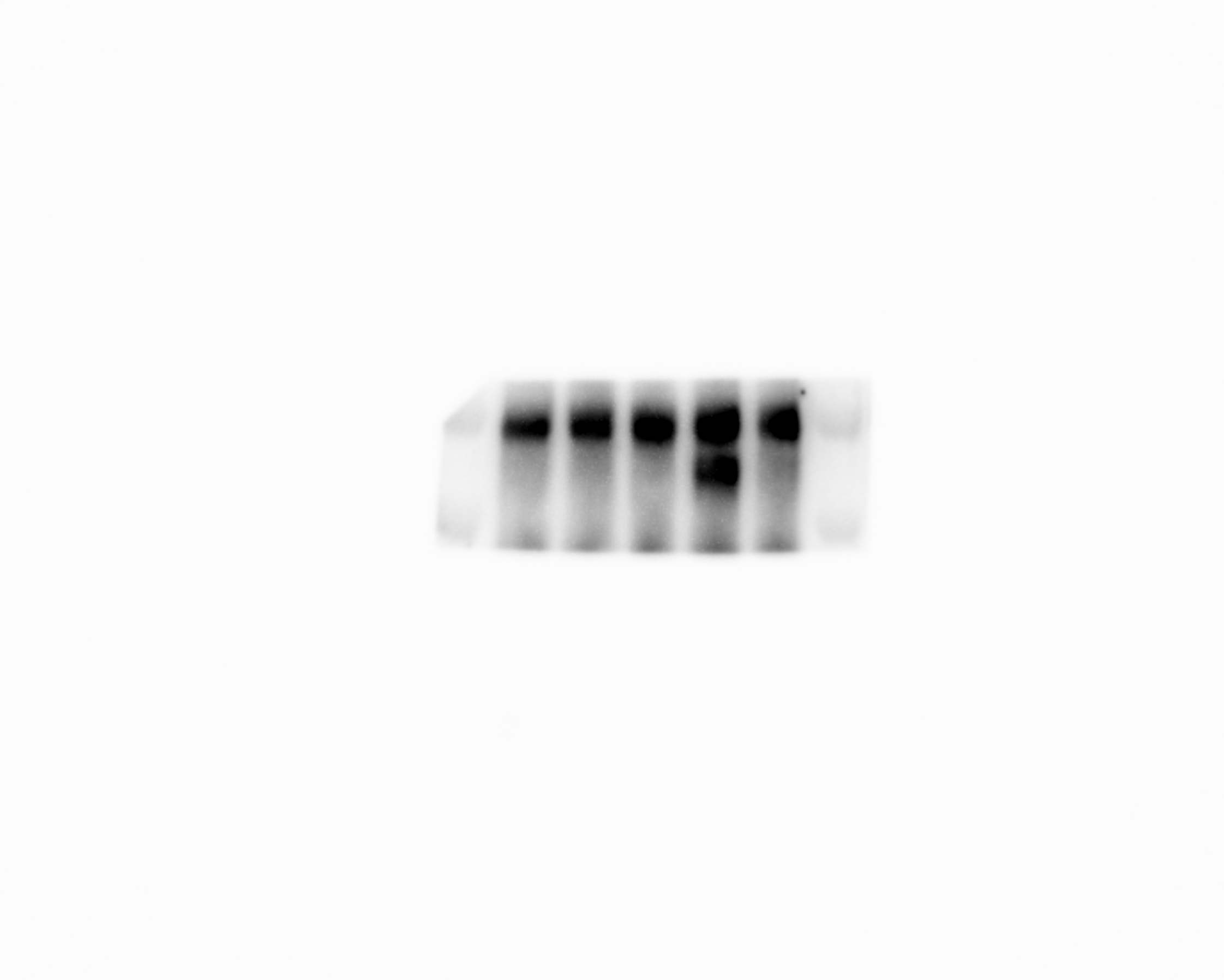

Supplement: Figure 4—source data 1. [file elife-69199-fig4-data1.zip › Figure 4A_Source Data/Figure_4A-Source_data_1_raw_ipFlag_anti_V5_immunoblot.tif]

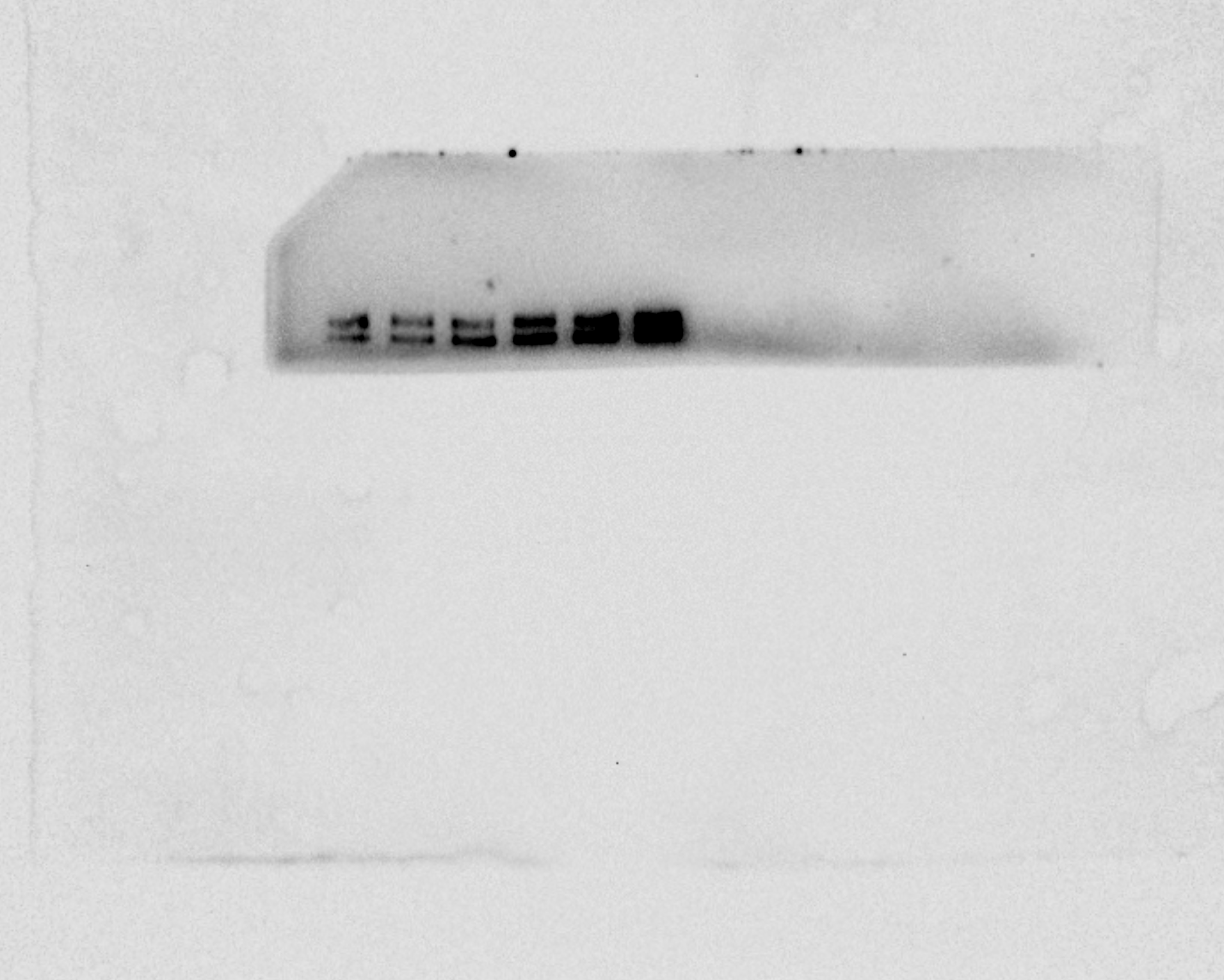

Supplement: Figure 4—source data 2. [file elife-69199-fig4-data2.zip › Figure 4F_Source Data/Figure_4F-Source_data_1_raw_WB_anti_APP_immunoblot.tif]

Figure 4F-Source data

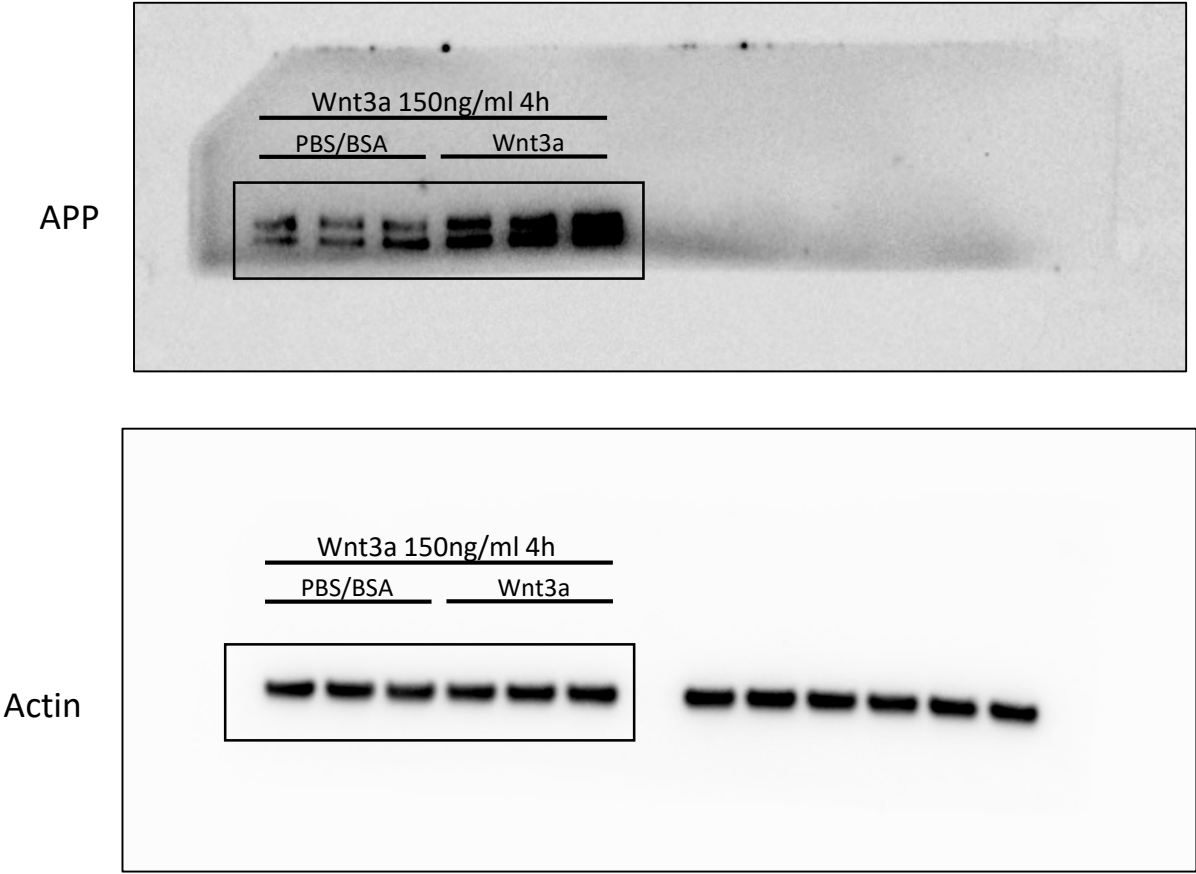

Supplement: Figure 4—source data 2. [file elife-69199-fig4-data2.zip › Figure 4F_Source Data/Figure 4F-Source data 1 labeled bands.pdf]

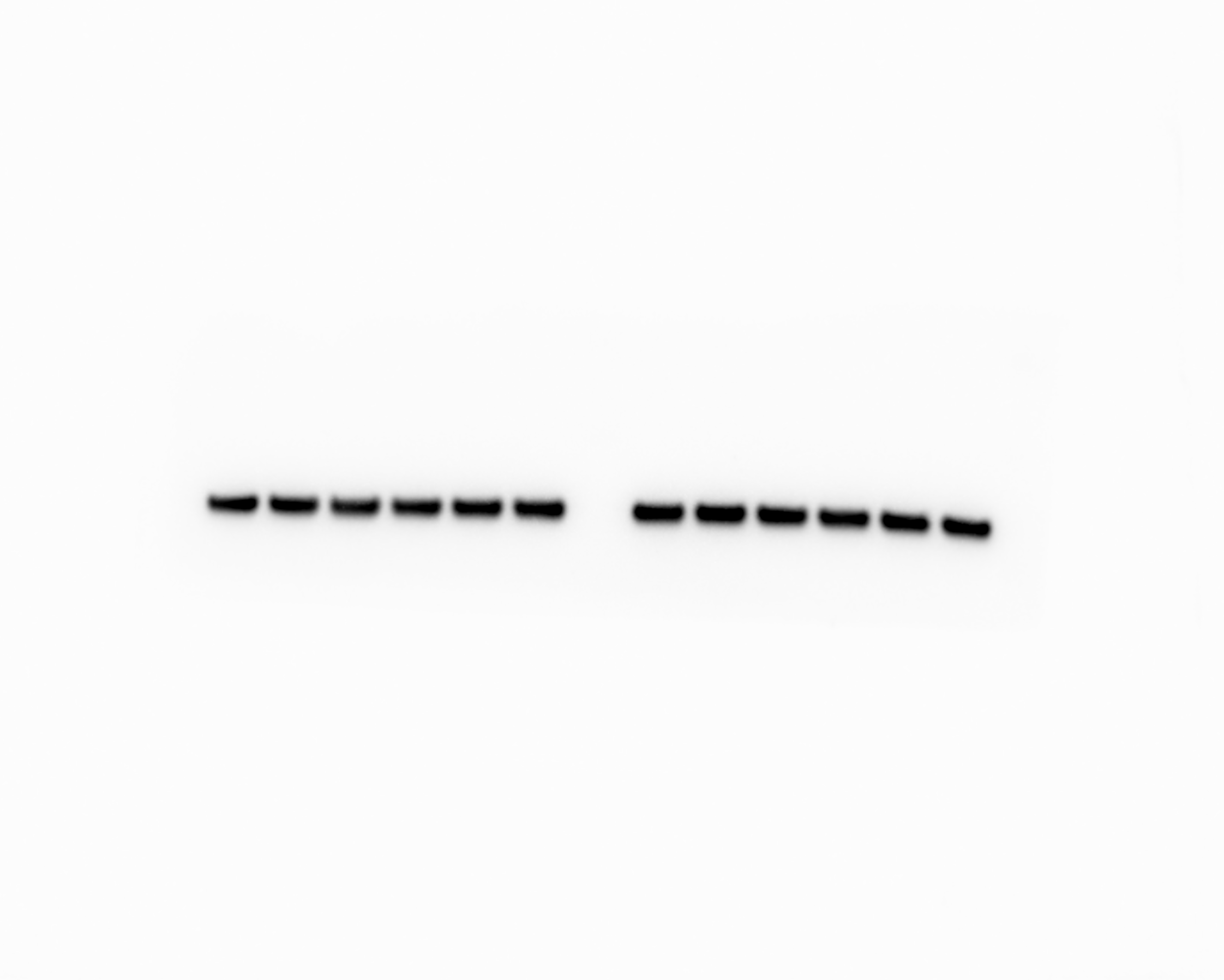

Supplement: Figure 4—source data 2. [file elife-69199-fig4-data2.zip › Figure 4F_Source Data/Figure_4F-Source_data_1_raw_WB_anti_Actin_immunoblot.tif]

Figure 4I-Source data

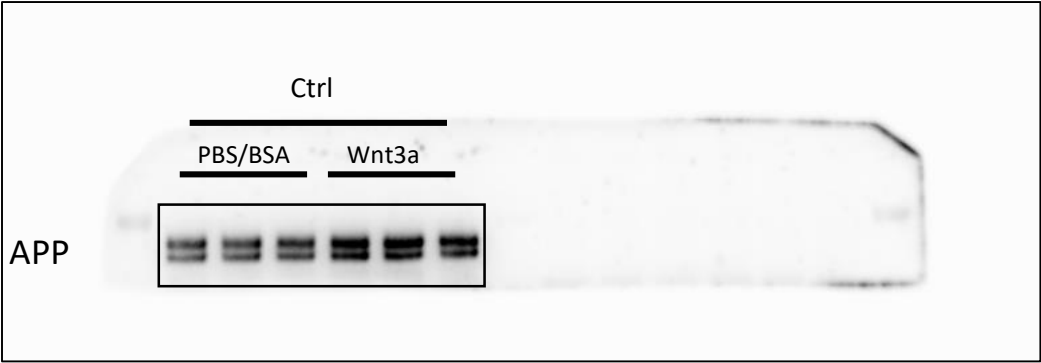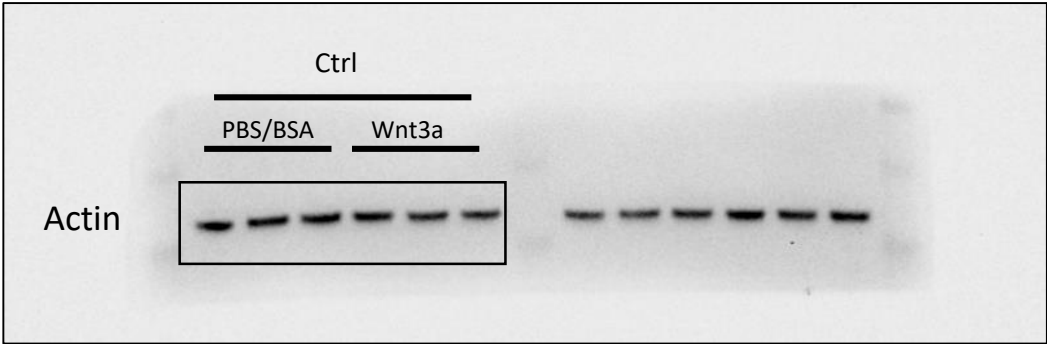

Supplement: Figure 4—source data 3. [file elife-69199-fig4-data3.zip › Figure 4I_Source Data/Figure 4I-Source data 1 labeled bands.pdf]

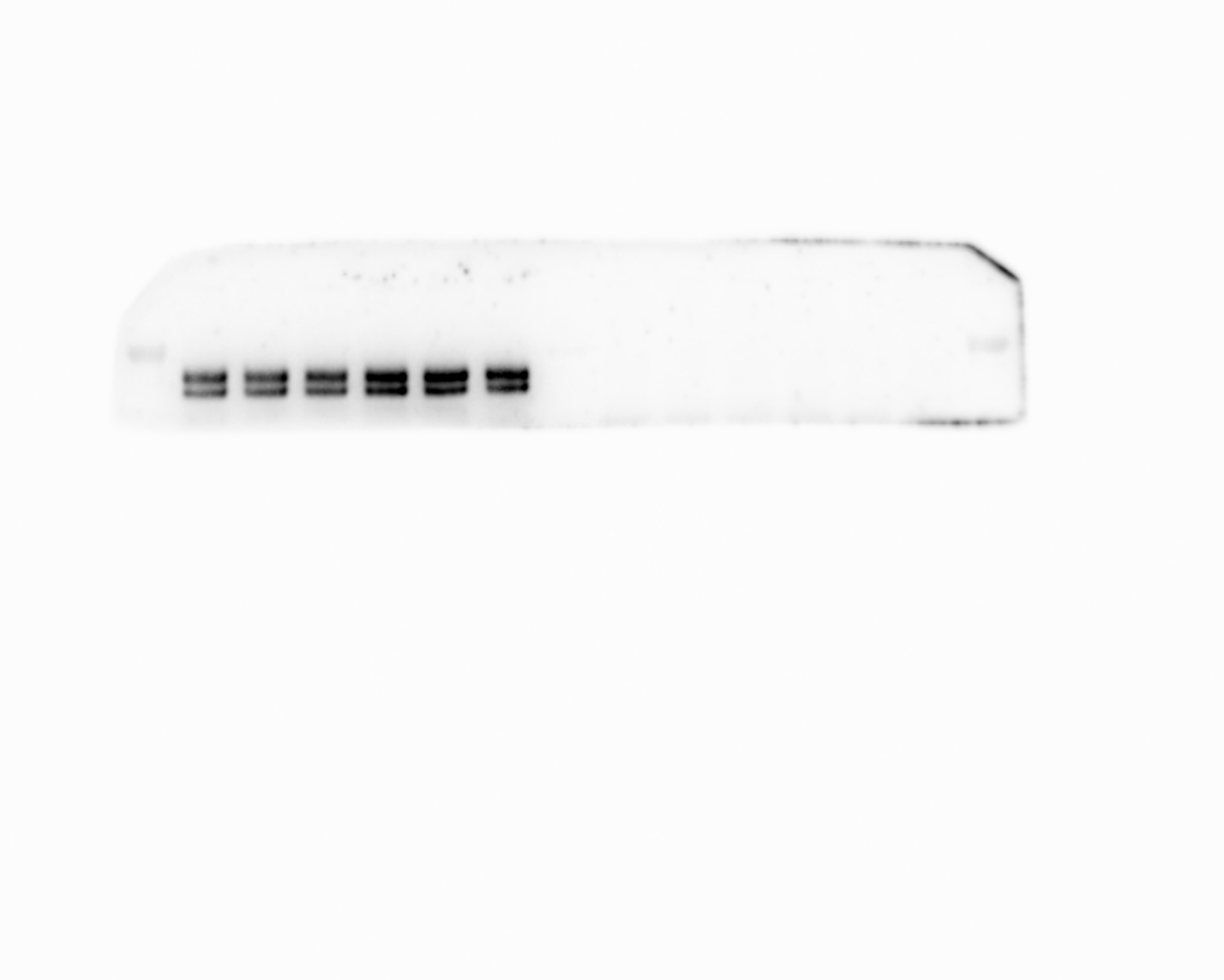

Supplement: Figure 4—source data 3. [file elife-69199-fig4-data3.zip › Figure 4I_Source Data/Figure_4I-Source_data_1_raw_WB_anti_APP_immunoblot.tif]

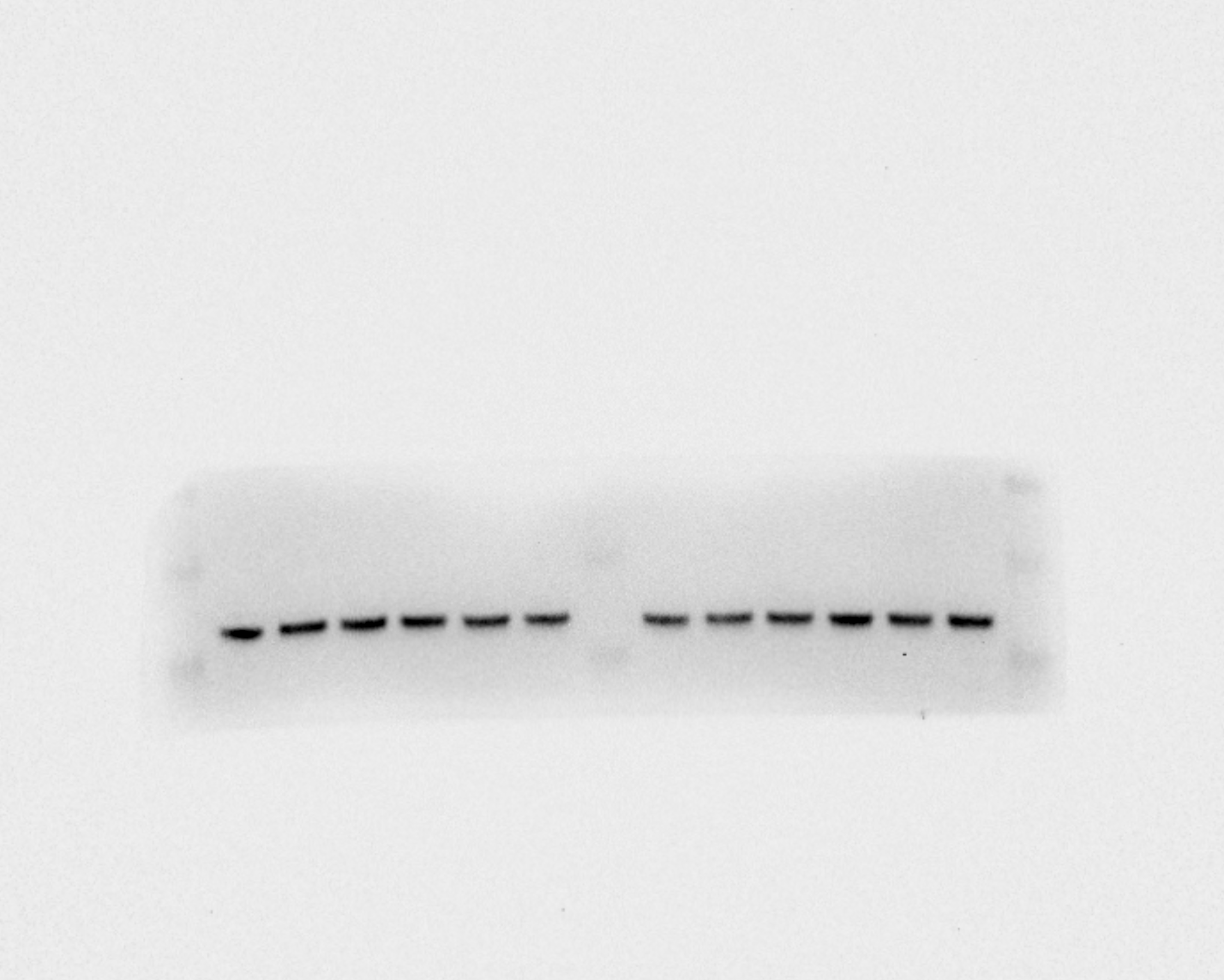

Supplement: Figure 4—source data 3. [file elife-69199-fig4-data3.zip › Figure 4I_Source Data/Figure_4I-Source_data_1_raw_WB_anti_Actin_immunoblot.tif]

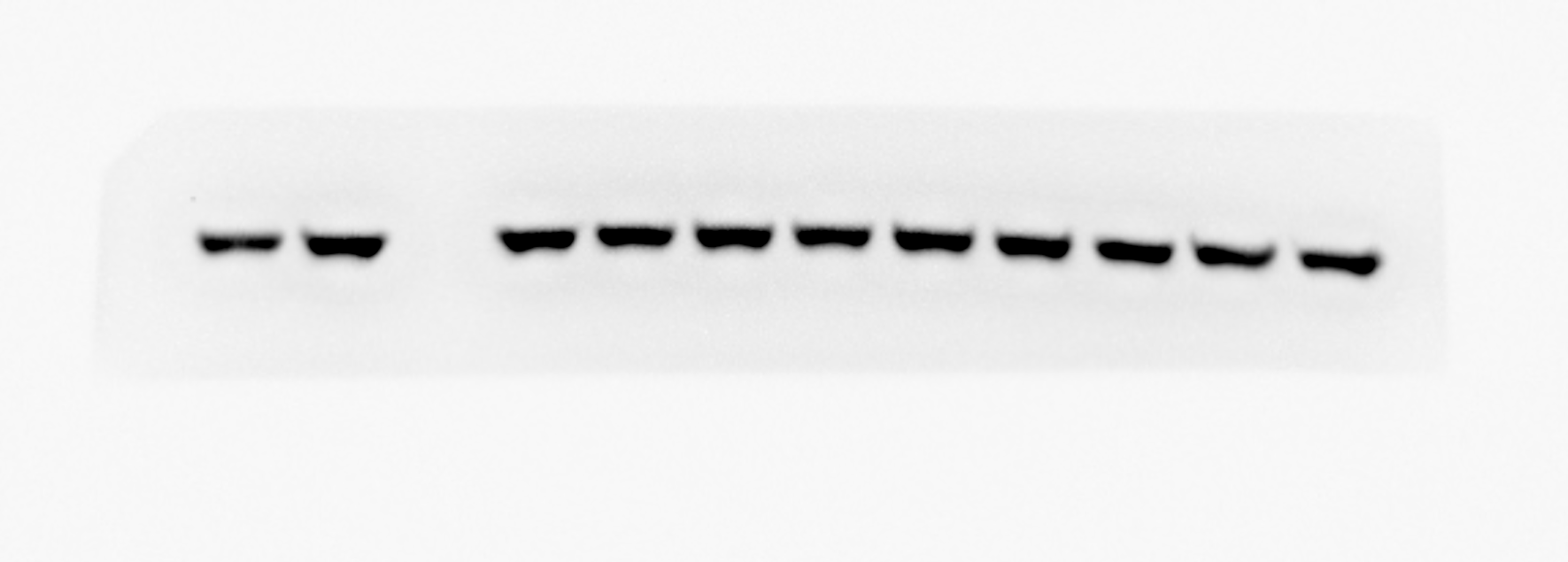

Supplement: Figure 4—source data 4. [file elife-69199-fig4-data4.zip › Figure 4J_Source Data/Figure_4J-Source_data_1_raw_WB_anti_Actin_immunoblot.tif]

Figure 4J-Source data

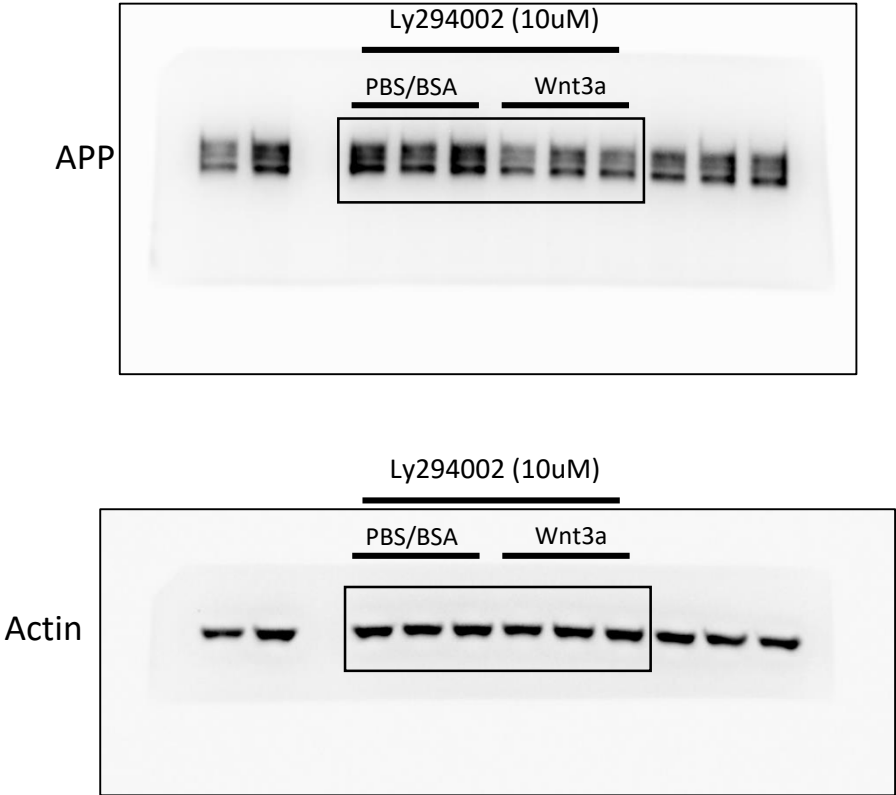

Supplement: Figure 4—source data 4. [file elife-69199-fig4-data4.zip › Figure 4J_Source Data/Figure 4J-Source data 1 labeled bands.pdf]

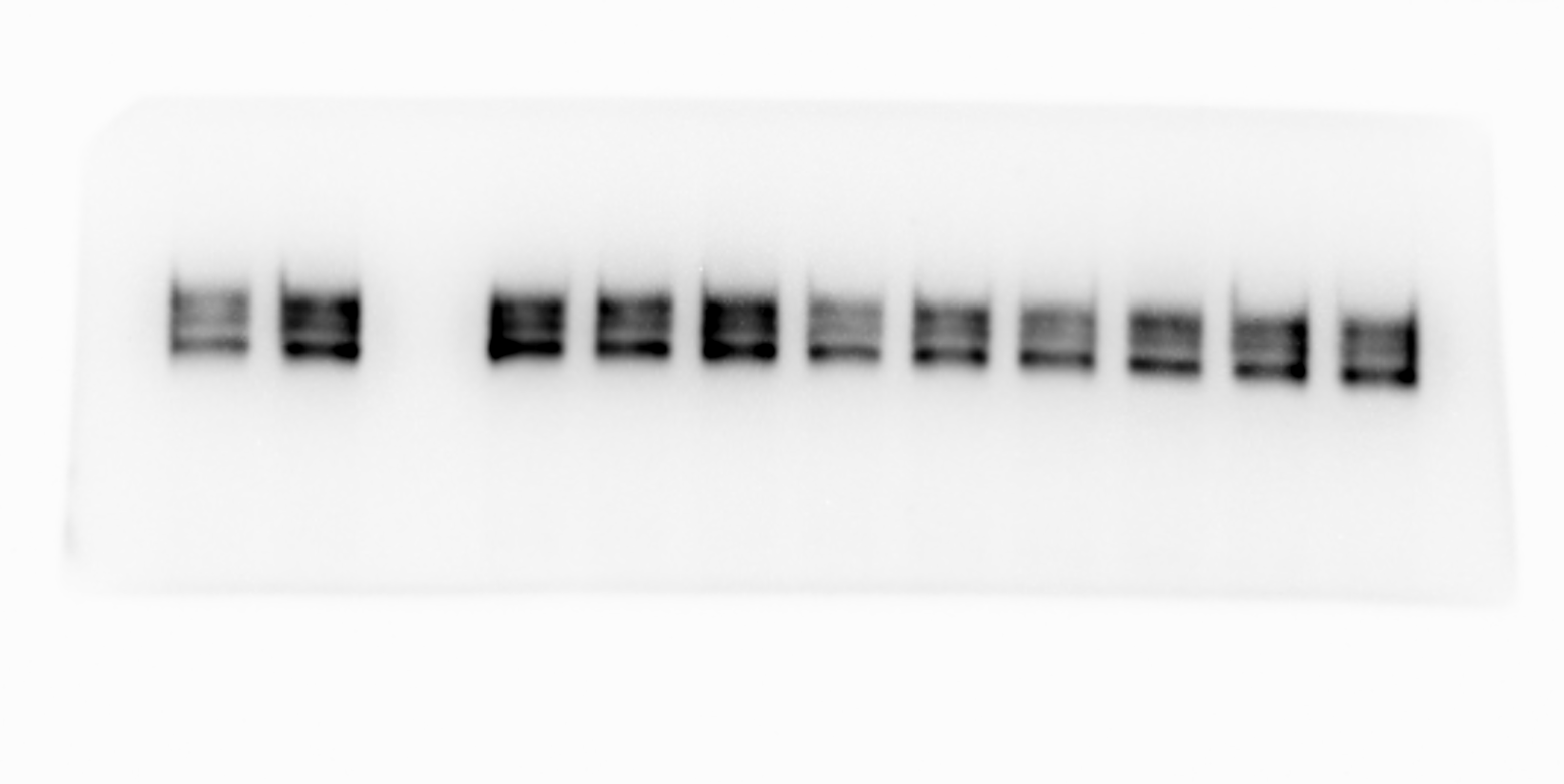

Supplement: Figure 4—source data 4. [file elife-69199-fig4-data4.zip › Figure 4J_Source Data/Figure_4J-Source_data_1_raw_WB_anti_APP_immunoblot.tif]

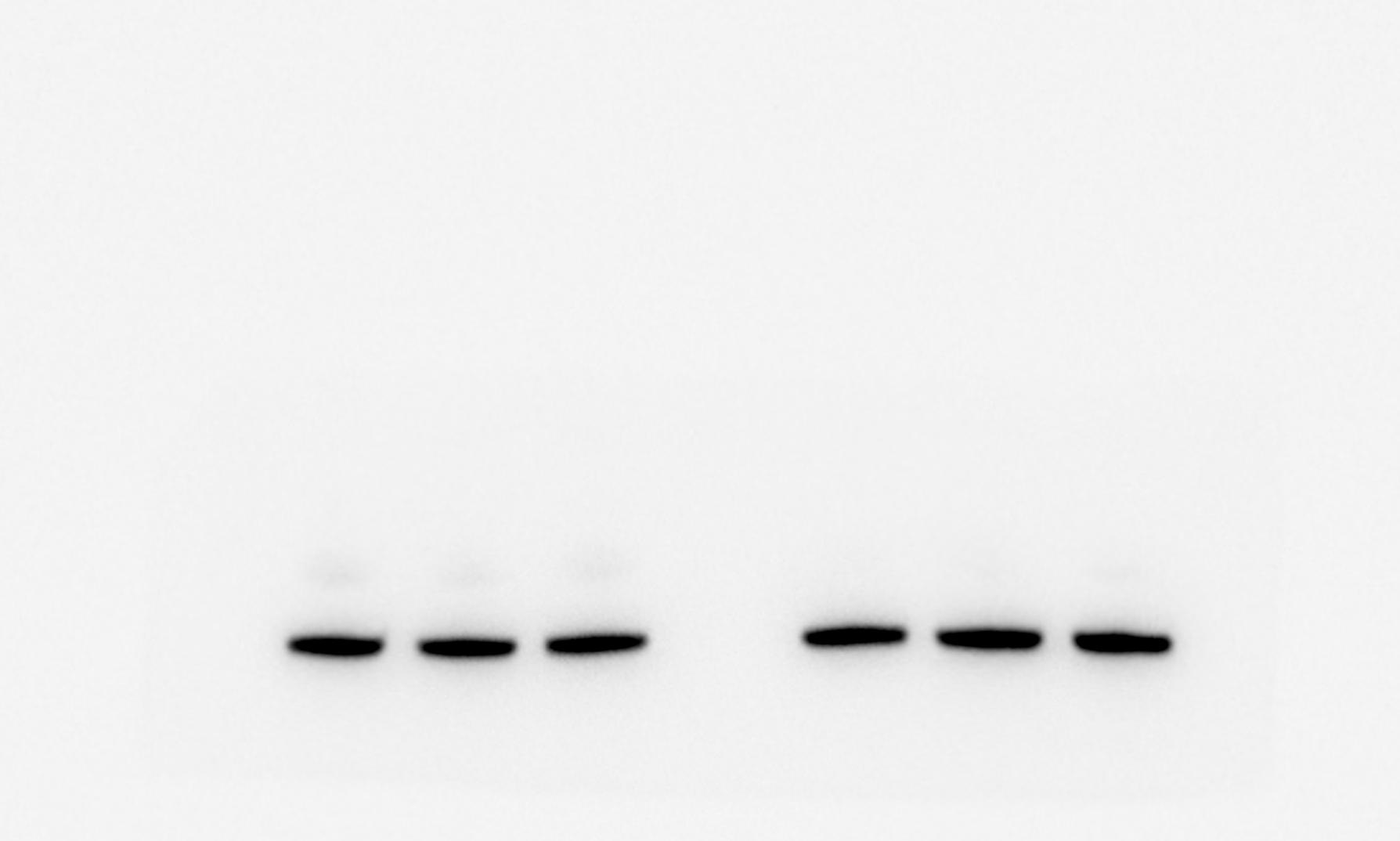

Supplement: Figure 4—source data 5. [file elife-69199-fig4-data5.zip › Figure 4M_Source Data/Figure_4M-Source_data_1_raw_WB_anti_Actin_immunoblot.tif]

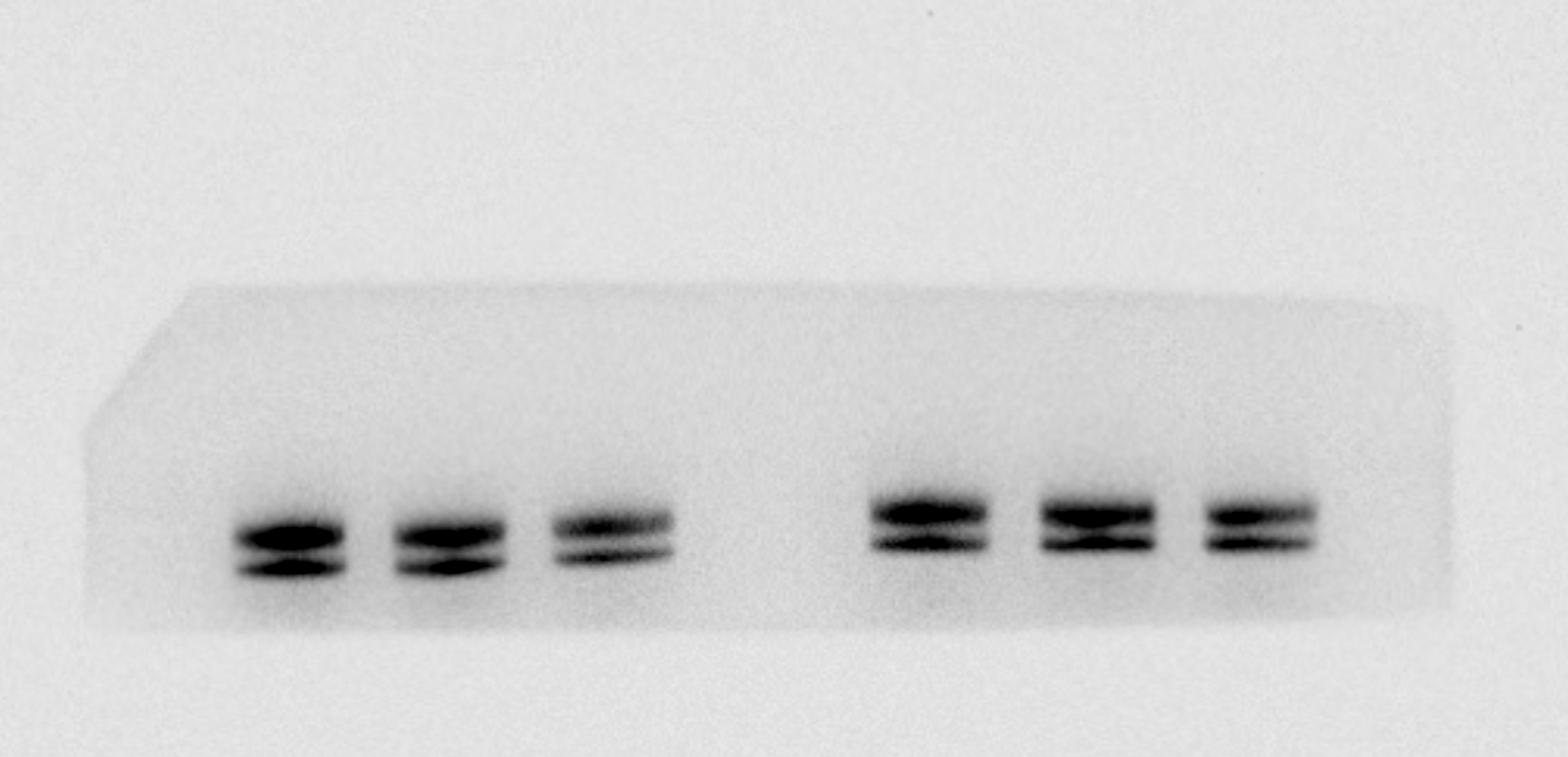

Supplement: Figure 4—source data 5. [file elife-69199-fig4-data5.zip › Figure 4M_Source Data/Figure_4M-Source_data_1_raw_WB_anti_APP_immunoblot.tif]

Figure 4M-Source data

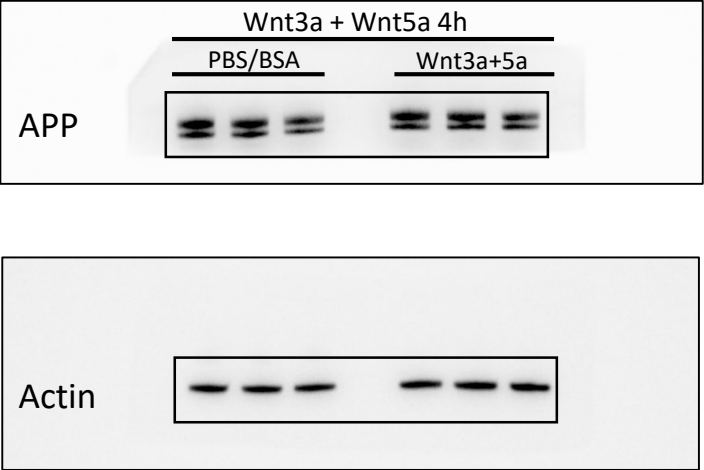

Supplement: Figure 4—source data 5. [file elife-69199-fig4-data5.zip › Figure 4M_Source Data/Figure 4M-Source data 1 labeled bands.pdf]

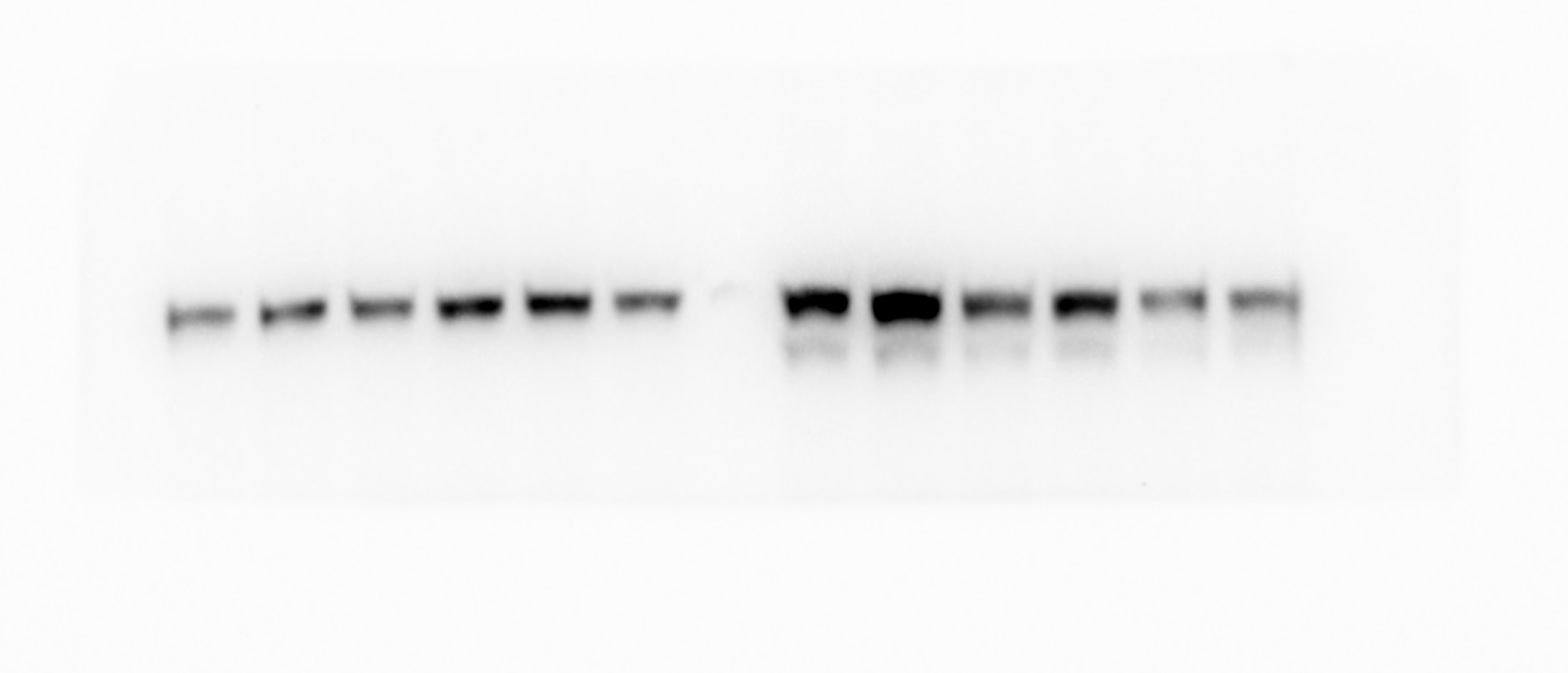

Supplement: Figure 4—figure supplement 1—source data 1. [file elife-69199-fig4-figsupp1-data1.zip › Figure 4-supplement 1A_Source Data/Figure 4-supplement 1-Source_data_1_raw_WB_anti_Catenin_immunoblot.tif]

Figure S7A-Source data

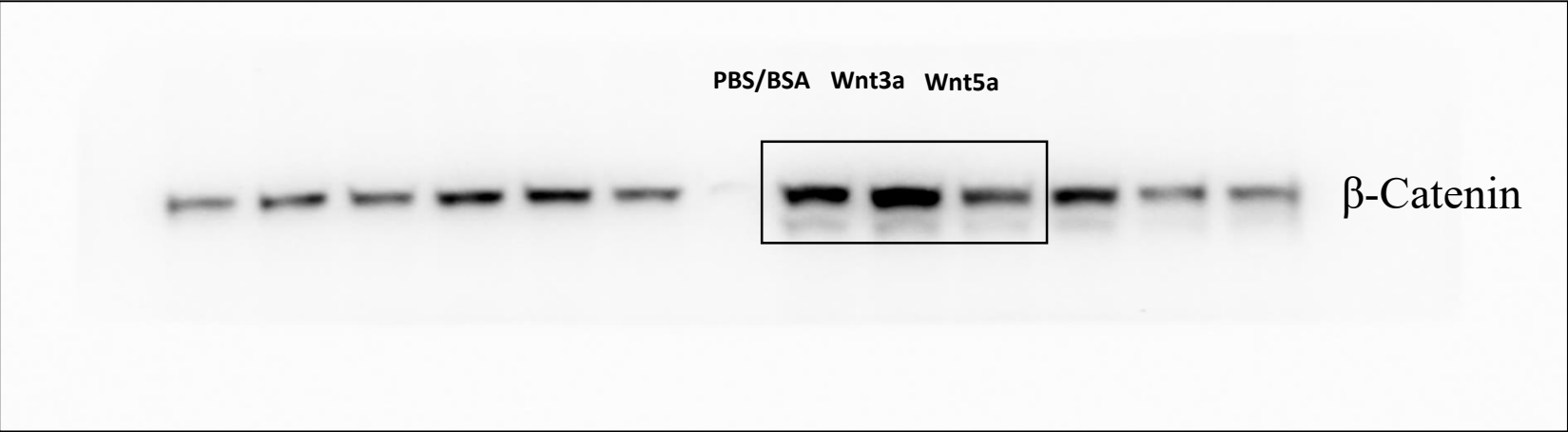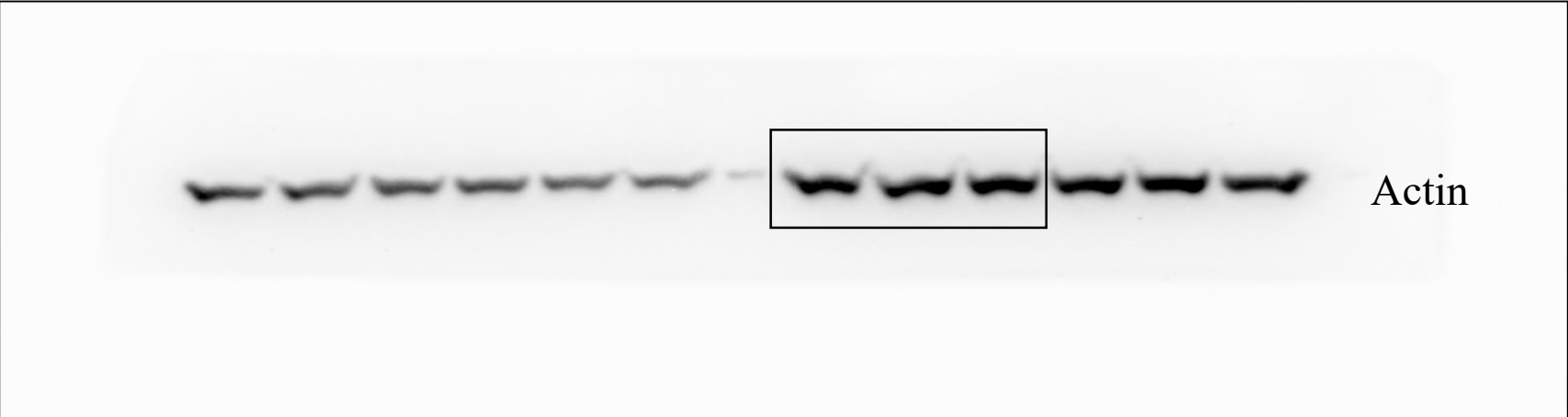

Supplement: Figure 4—figure supplement 1—source data 1. [file elife-69199-fig4-figsupp1-data1.zip › Figure 4-supplement 1A_Source Data/Figure 4-supplement 1A-Source data 1 labeled bands.pdf]

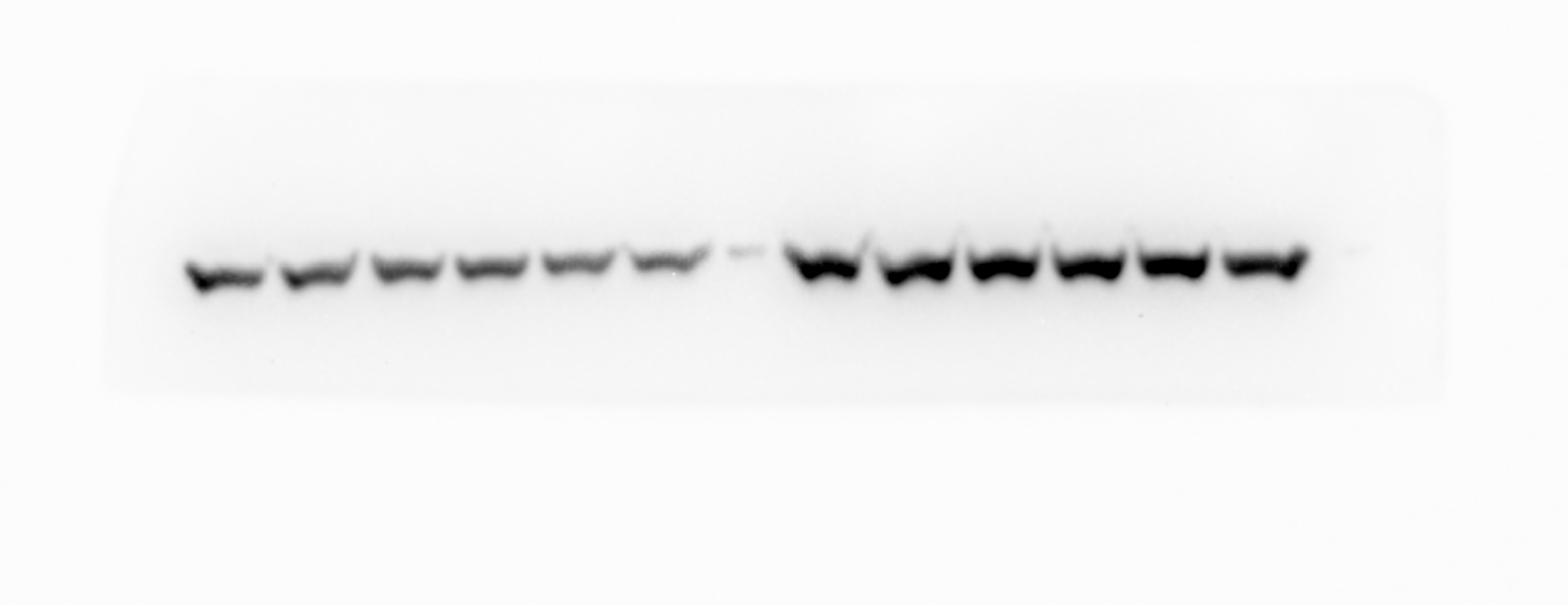

Supplement: Figure 4—figure supplement 1—source data 1. [file elife-69199-fig4-figsupp1-data1.zip › Figure 4-supplement 1A_Source Data/Figure 4-supplement 1A -Source_data_1_raw_WB_anti_Actin_immunoblot.tif]

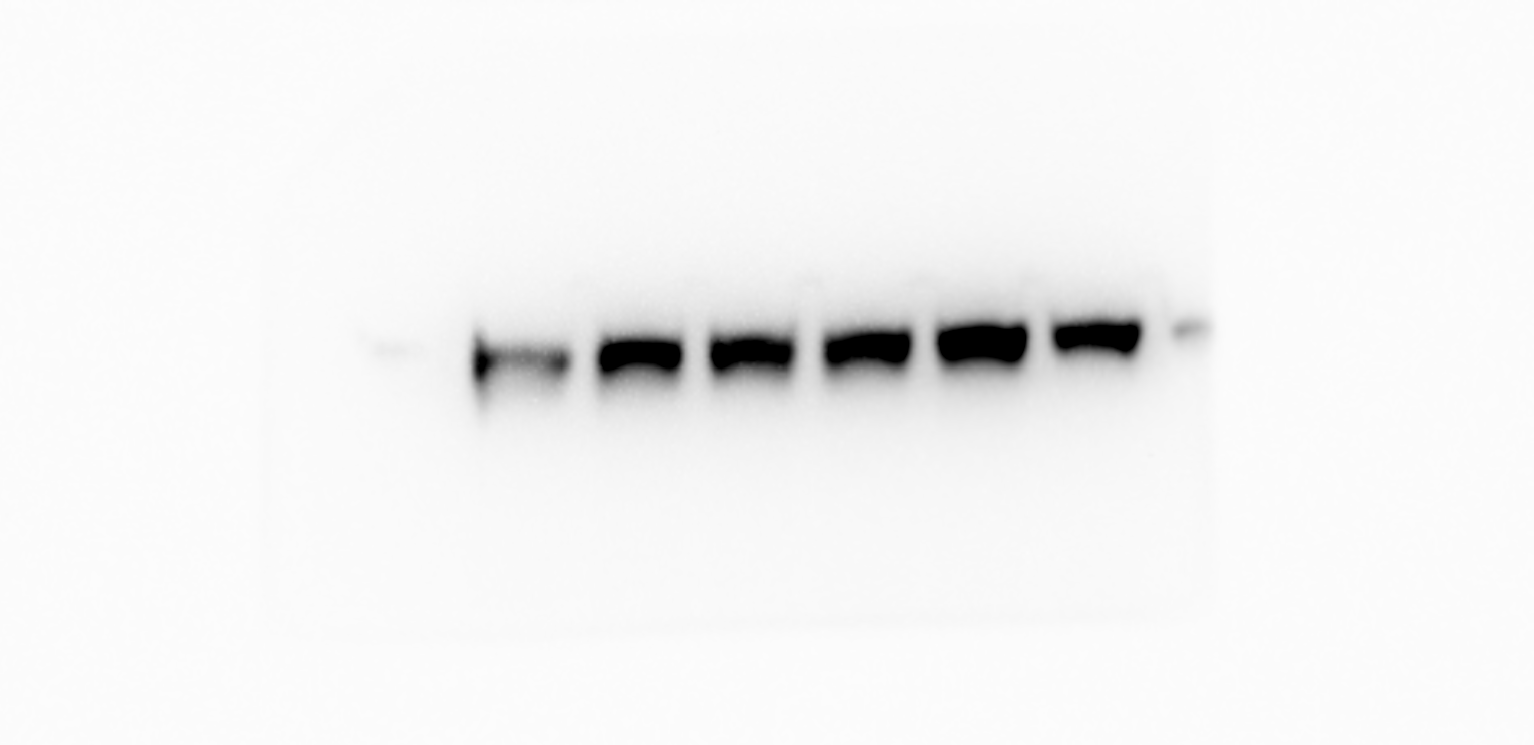

Supplement: Figure 4—figure supplement 1—source data 2. [file elife-69199-fig4-figsupp1-data2.zip › Figure 4-supplement 1B_Source Data/Figure 4-supplement 1B-Source_data_1_raw_WB_anti_Catenin.tif]

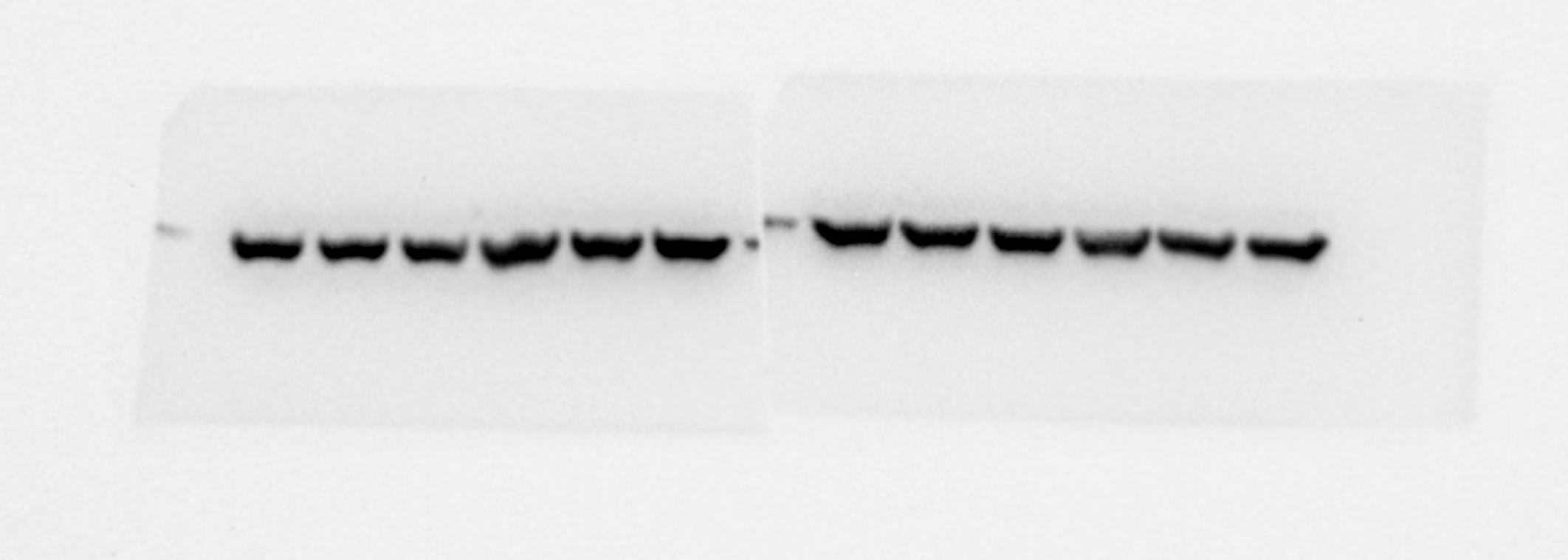

Supplement: Figure 4—figure supplement 1—source data 2. [file elife-69199-fig4-figsupp1-data2.zip › Figure 4-supplement 1B_Source Data/Figure 4-supplement 1B-Source_data_1_raw_WB_anti_Actin.tif]

Figure S7B-Source data

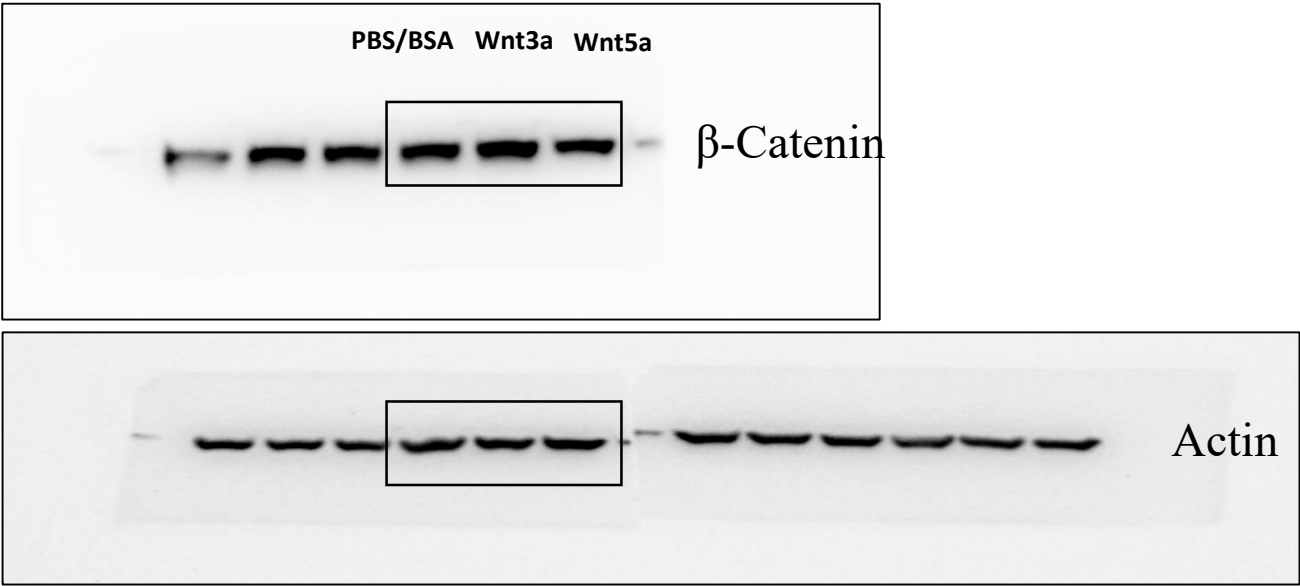

Supplement: Figure 4—figure supplement 1—source data 2. [file elife-69199-fig4-figsupp1-data2.zip › Figure 4-supplement 1B_Source Data/Figure 4-supplement 1B-Source data 1 labeled bands.pdf]

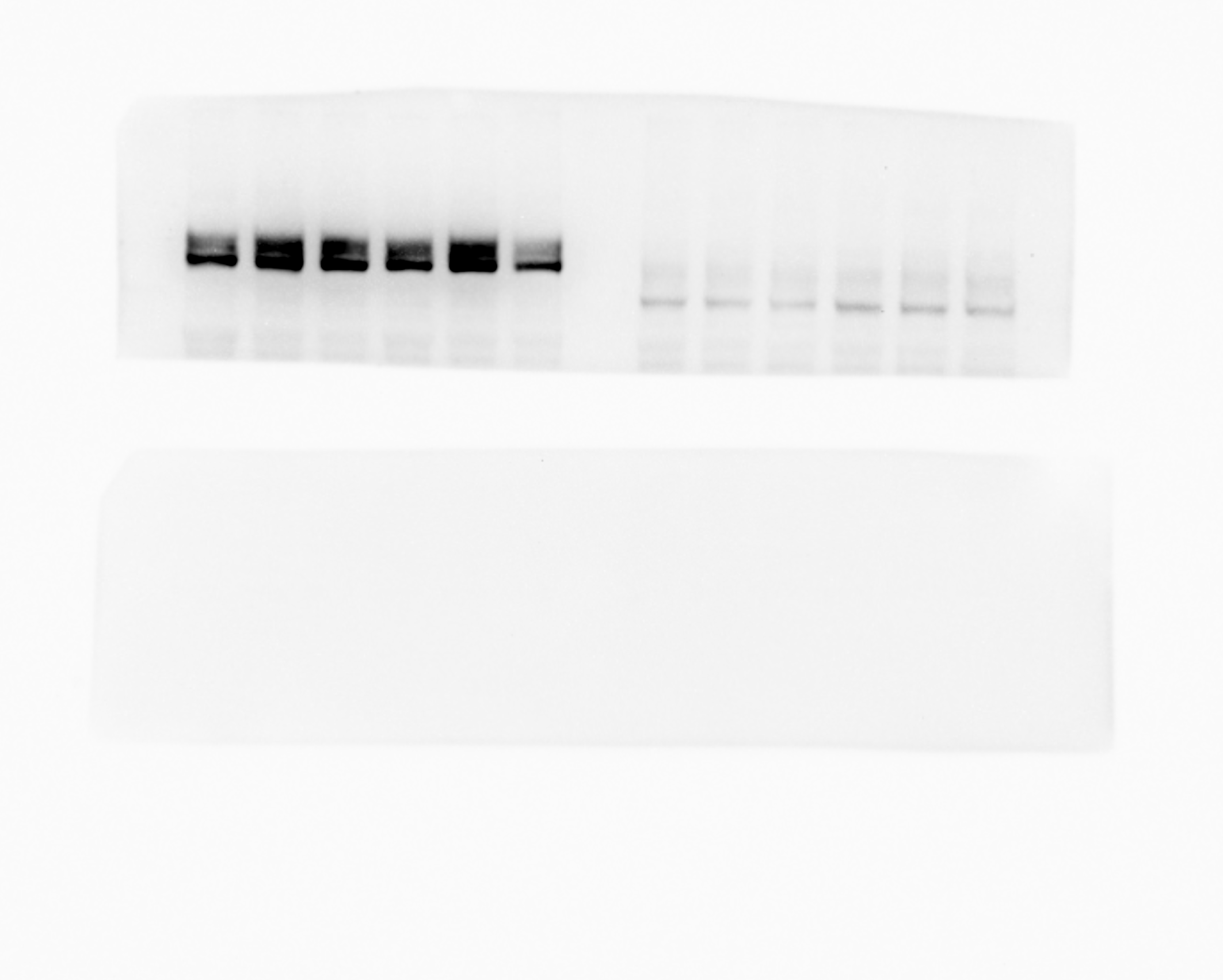

Supplement: Figure 5—source data 1. [file elife-69199-fig5-data1.zip › Figure 5A_Source Data/Figure_5A-Source_data_1_raw_WB_anti_APP_immunoblot.tif]

Figure 5A-Source data

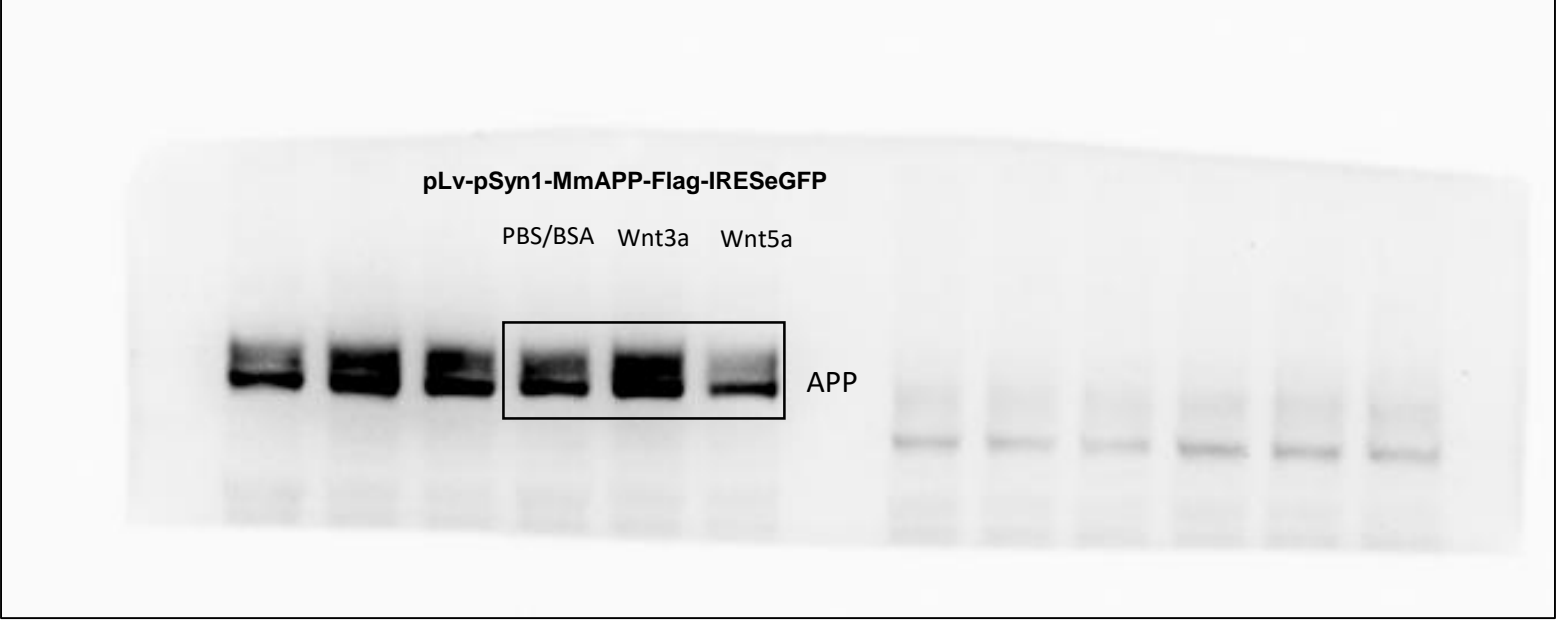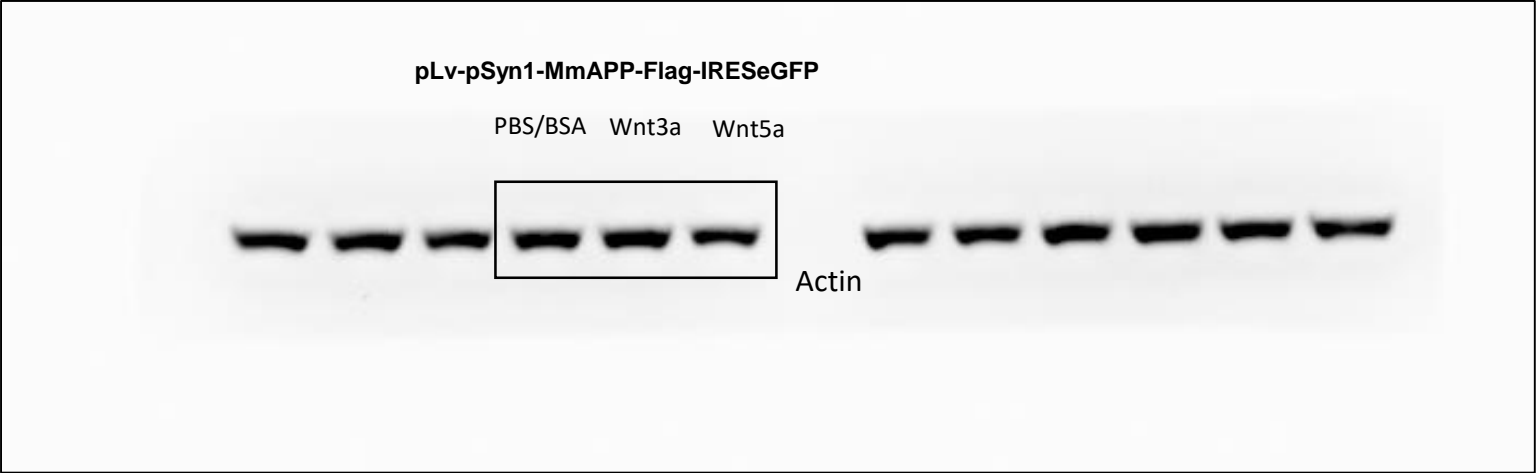

Supplement: Figure 5—source data 1. [file elife-69199-fig5-data1.zip › Figure 5A_Source Data/Figure 5A-Source data 1 labeled bands.pdf]

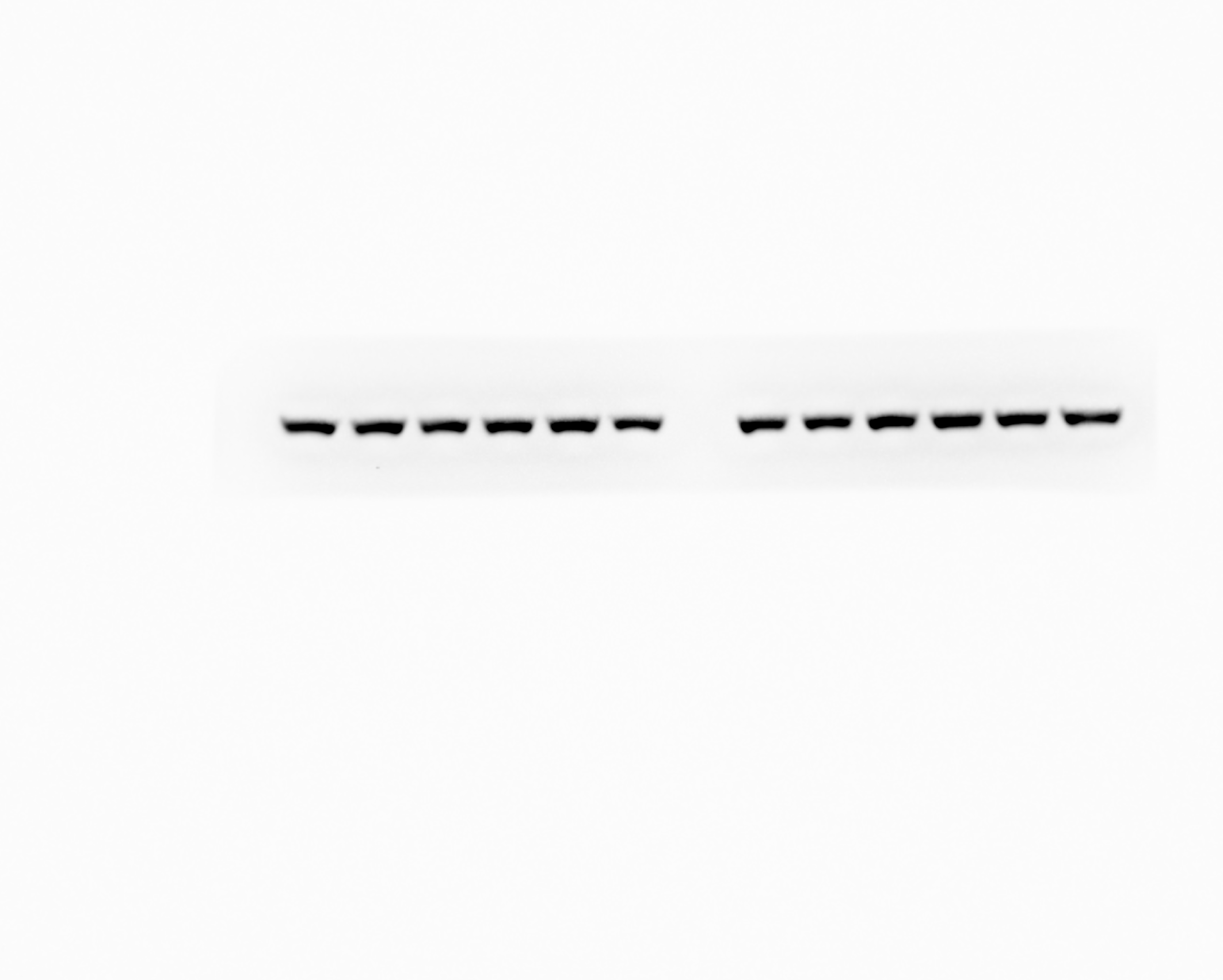

Supplement: Figure 5—source data 1. [file elife-69199-fig5-data1.zip › Figure 5A_Source Data/Figure_5A-Source_data_1_raw_WB_anti_Actin_immunoblot.tif]

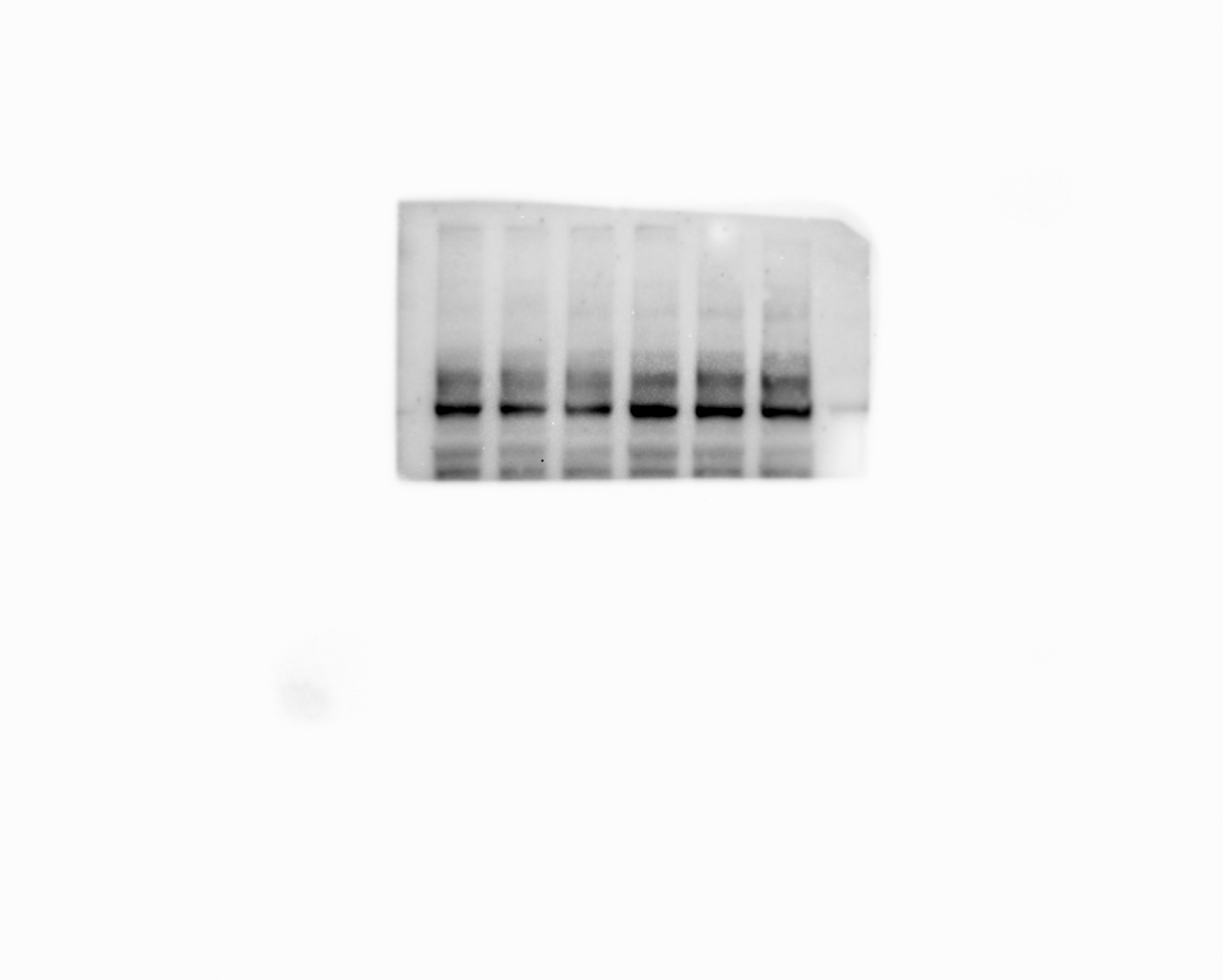

Supplement: Figure 5—source data 2. [file elife-69199-fig5-data2.zip › Figure 5B_Source Data/Figure_5B-Source_data_1_raw_WB_anti_APP_immunoblot.tif]

Figure 5B-Source data

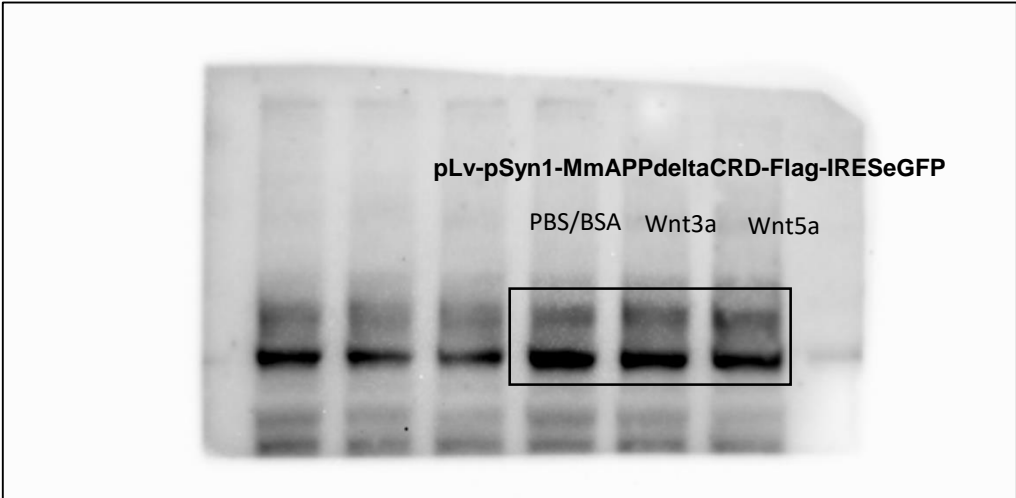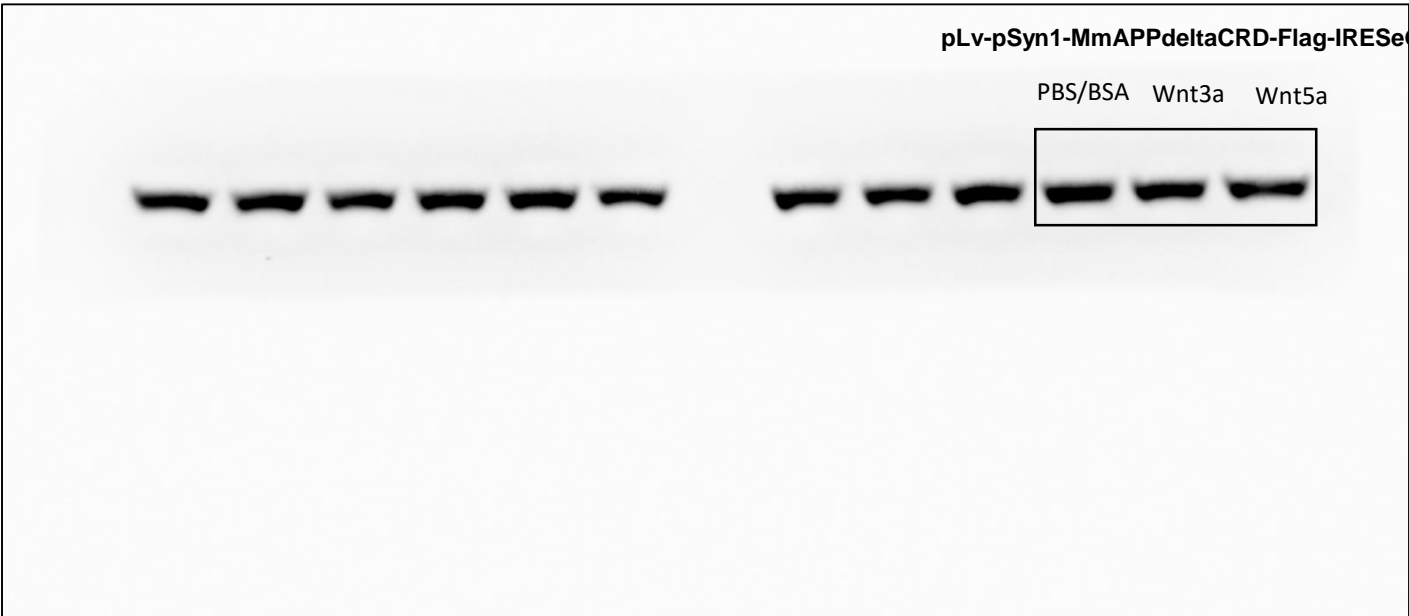

Supplement: Figure 5—source data 2. [file elife-69199-fig5-data2.zip › Figure 5B_Source Data/Figure 5B-Source data 1 labeled bands.pdf]

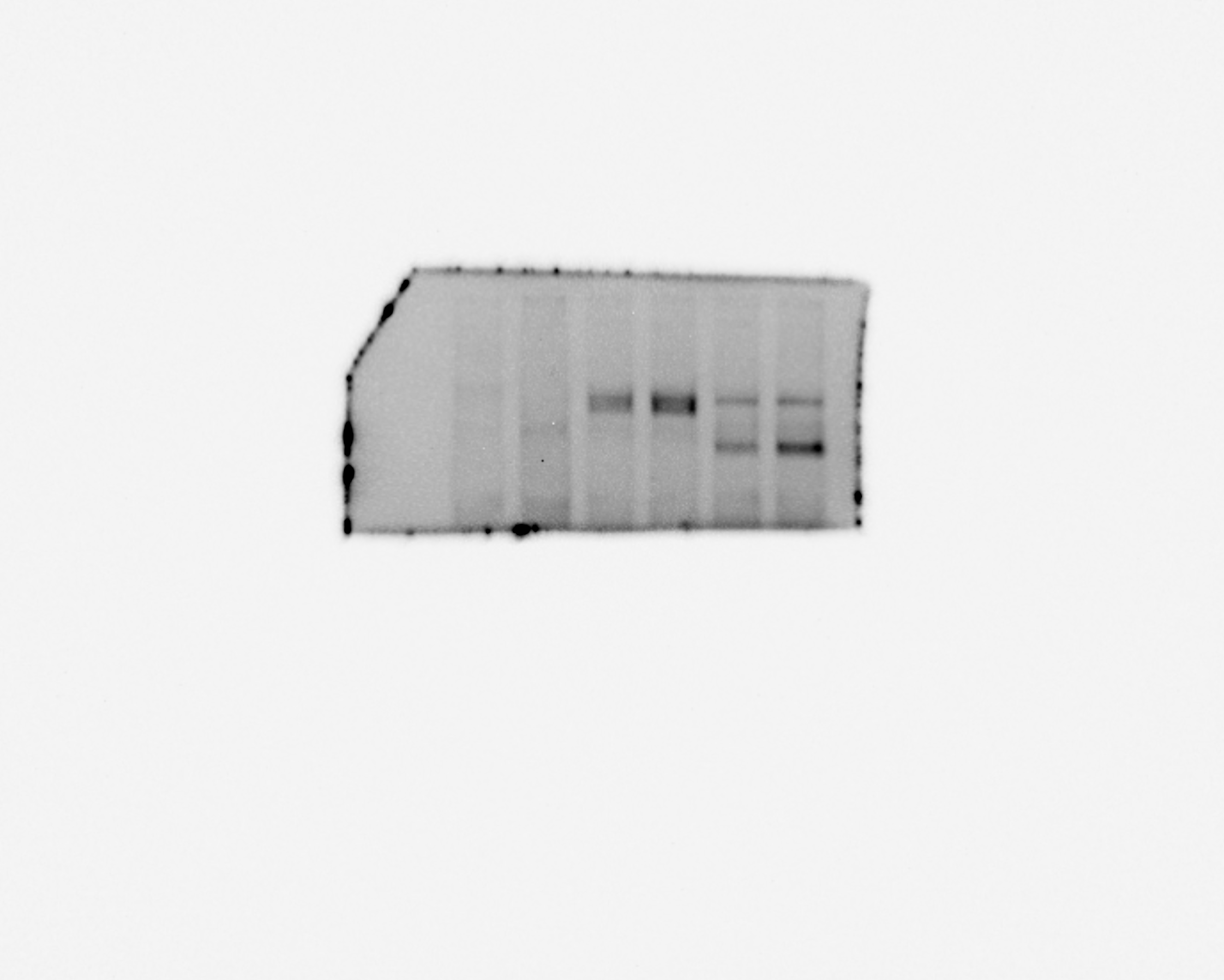

Supplement: Figure 5—figure supplement 1—source data 1. [file elife-69199-fig5-figsupp1-data1.zip › Figure 5 supplement 1B_Source Data/Fig 5 supplement 1B-Source_data_1_raw_WB_anti_Flag.tif]

Figure S9B-Source data

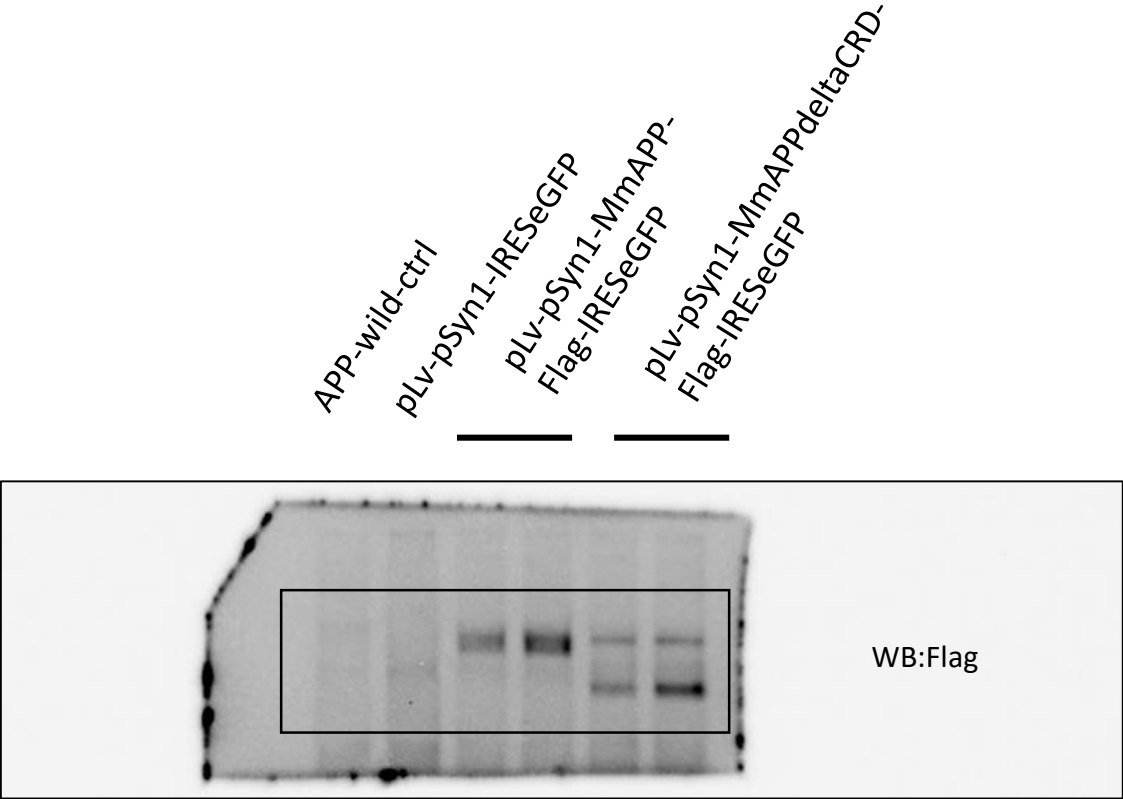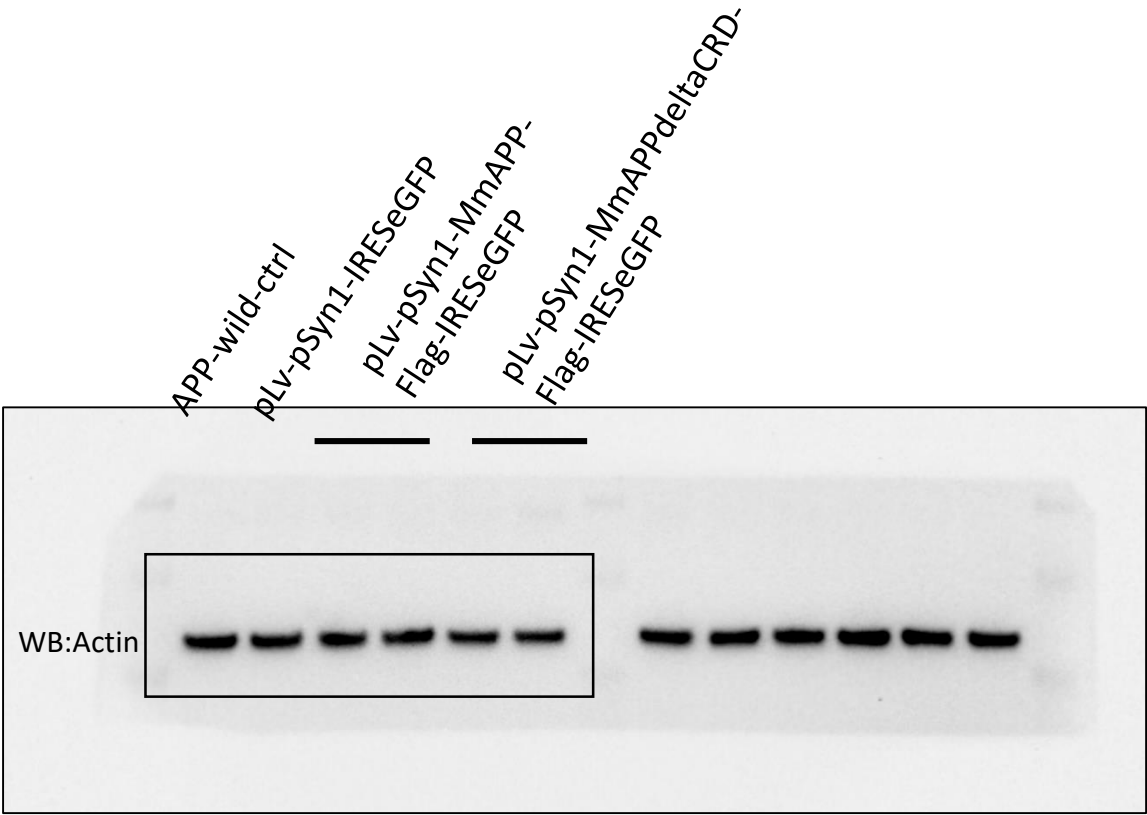

Supplement: Figure 5—figure supplement 1—source data 1. [file elife-69199-fig5-figsupp1-data1.zip › Figure 5 supplement 1B_Source Data/Fig 5 supplement 1B-Source data 1 labeled bands.pdf]

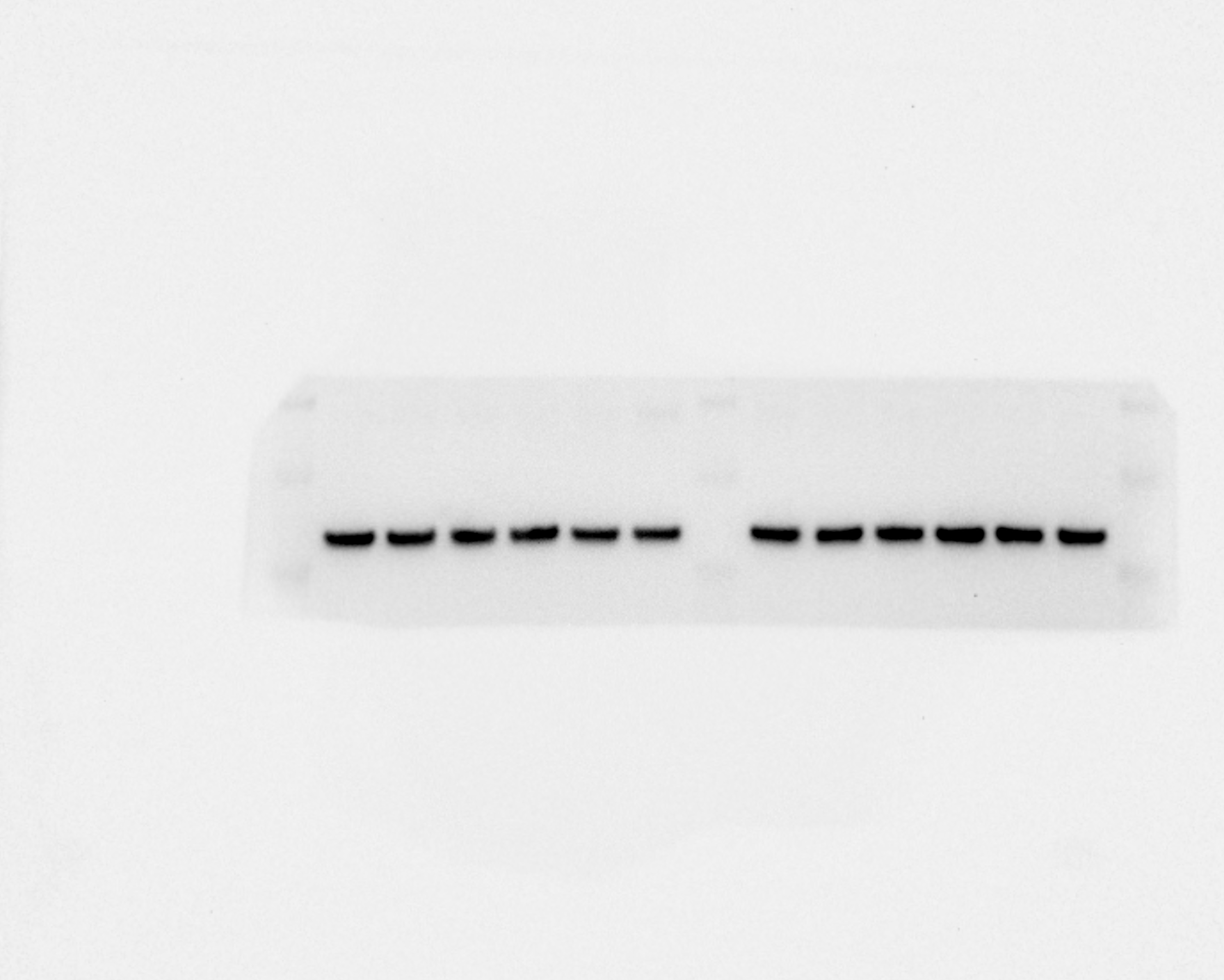

Supplement: Figure 5—figure supplement 1—source data 1. [file elife-69199-fig5-figsupp1-data1.zip › Figure 5 supplement 1B_Source Data/Fig 5 supplement 1B-Source_data_1_raw_WB_anti_Actin.tif]

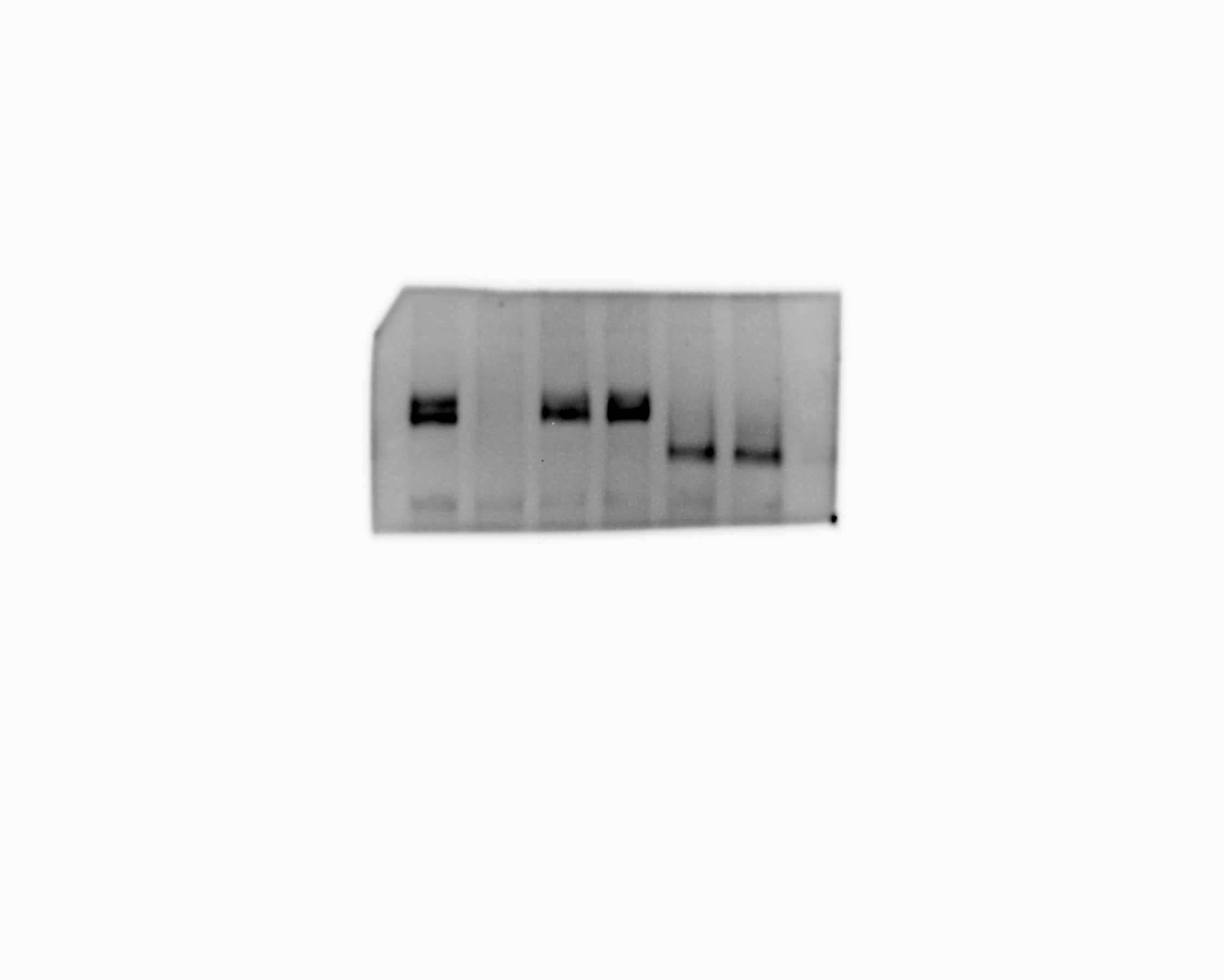

Supplement: Figure 5—figure supplement 1—source data 2. [file elife-69199-fig5-figsupp1-data2.zip › Figure 5 supplement 1C_Source Data/Fig 5 supplement 1C-Source_data_1_raw_WB_anti_APP.tif]

Figure S9C-Source data

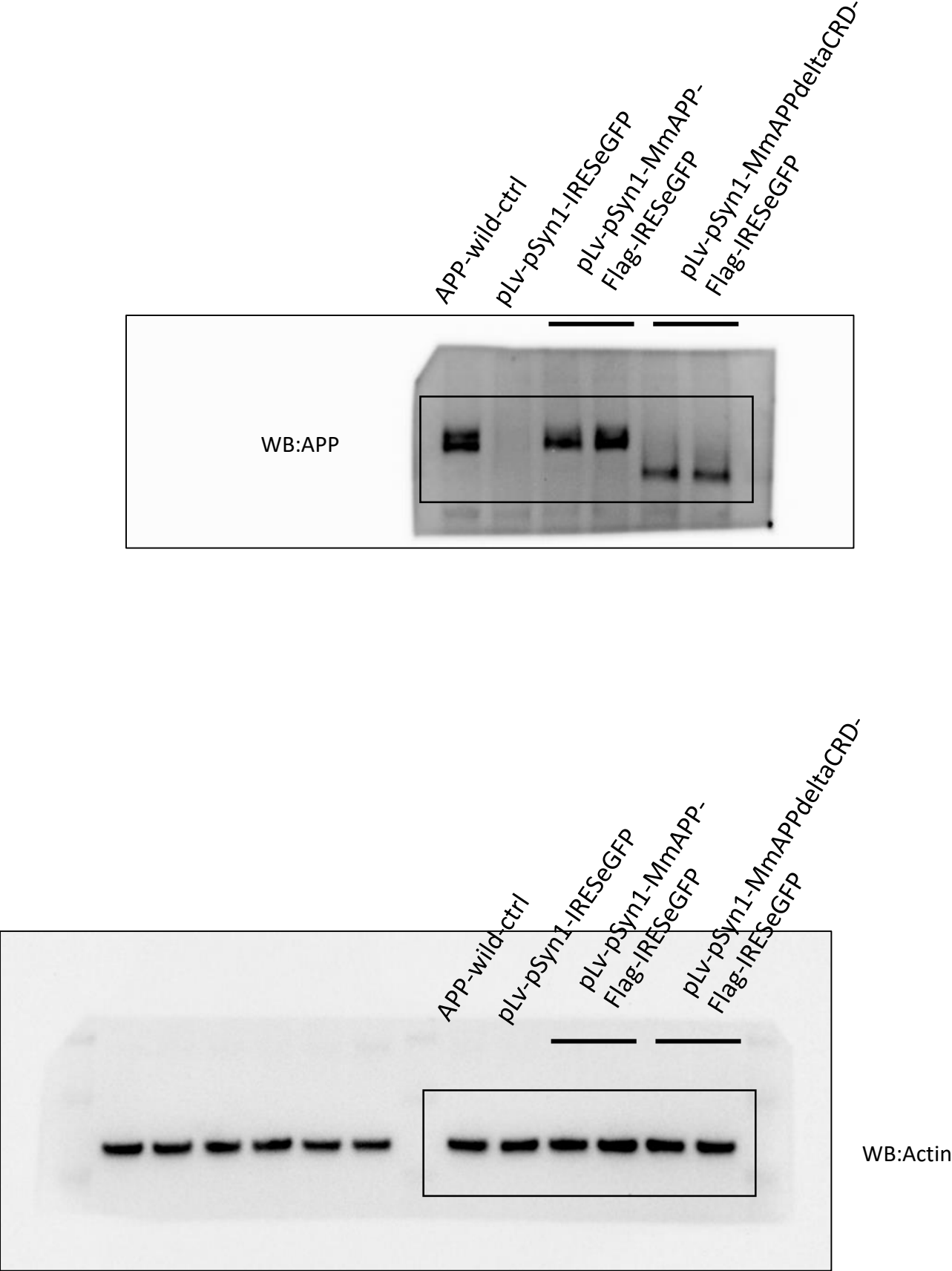

Supplement: Figure 5—figure supplement 1—source data 2. [file elife-69199-fig5-figsupp1-data2.zip › Figure 5 supplement 1C_Source Data/Fig 5 supplement 1C-Source data 1 labeled bands.pdf]
