# Supplementary material for: The amyloid precursor protein is a conserved Wnt receptor
Source: eLife. 2021 Sep 9;10:e69199. doi: 10.7554/eLife.69199 (PMC8437438; doi:10.7554/eLife.69199)

Figure S6A-Source data

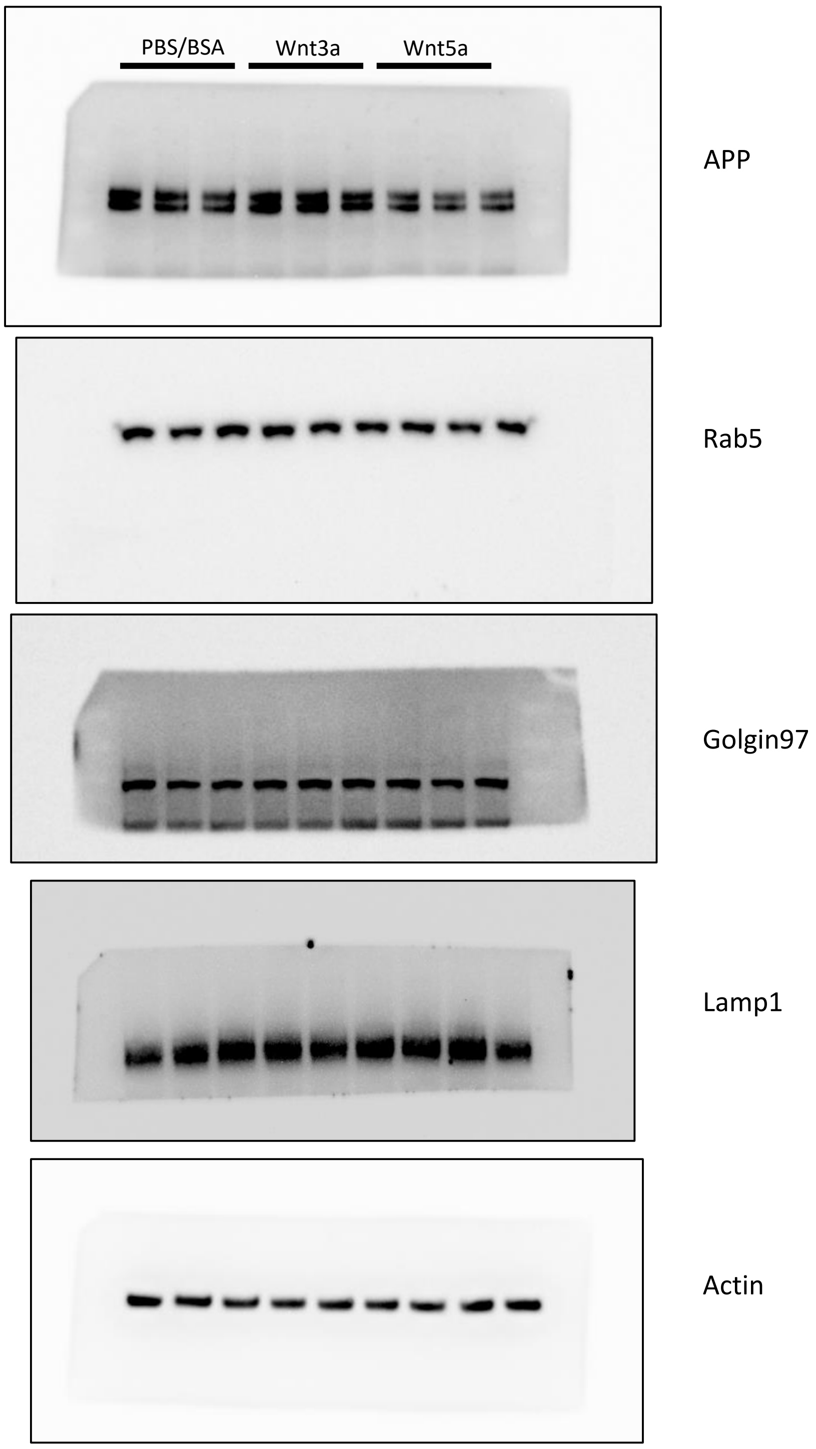

Figure S7A-Source data

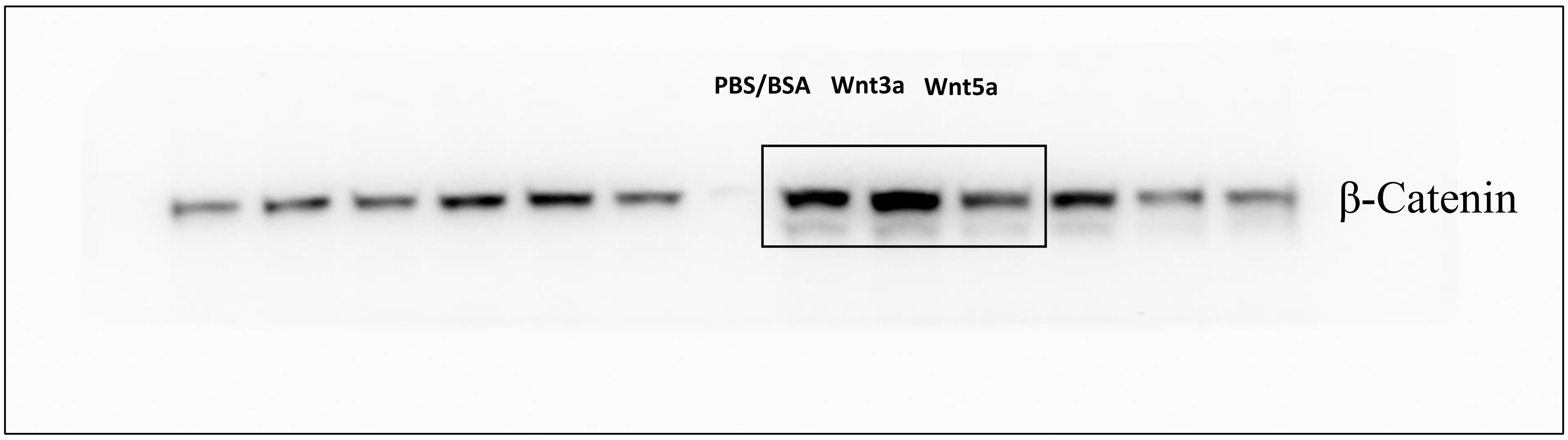

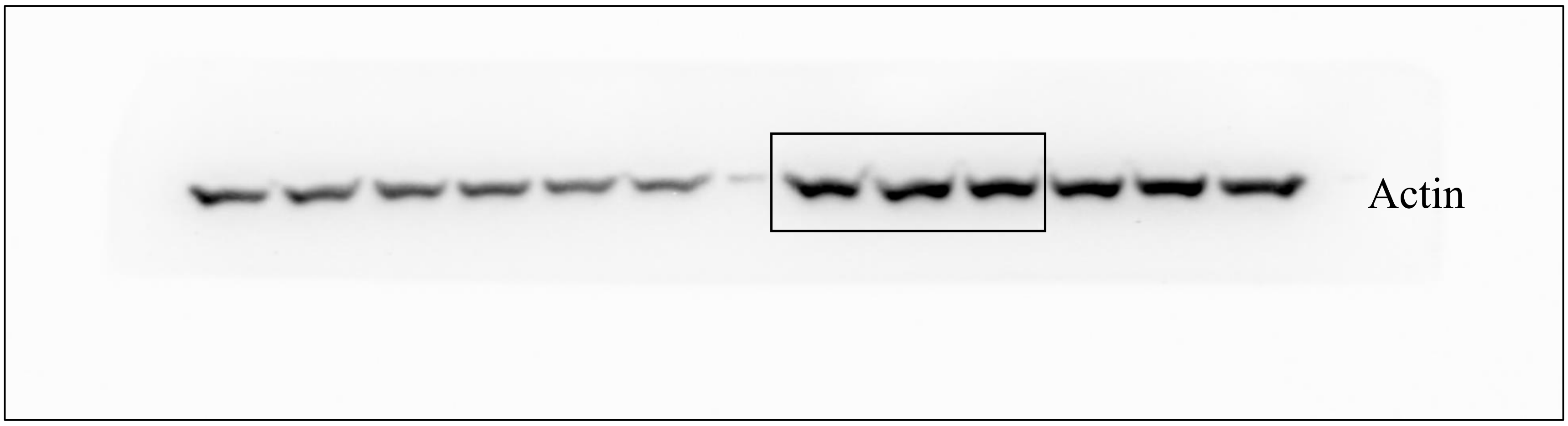

Figure S7B-Source data

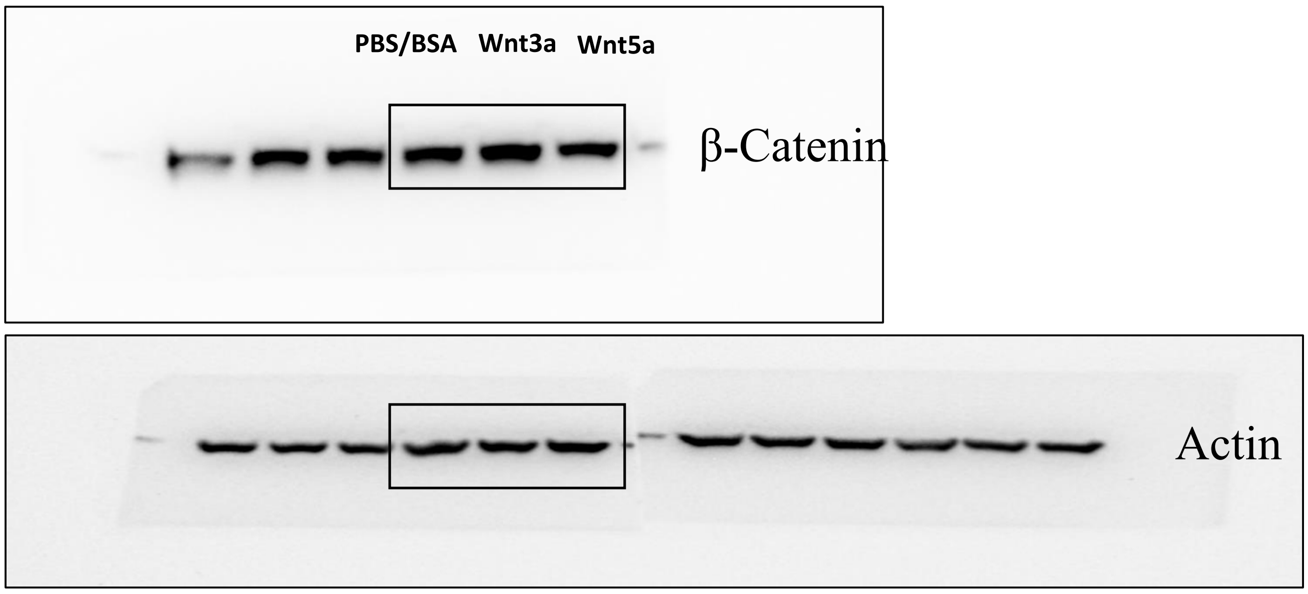

Figure S9B-Source data

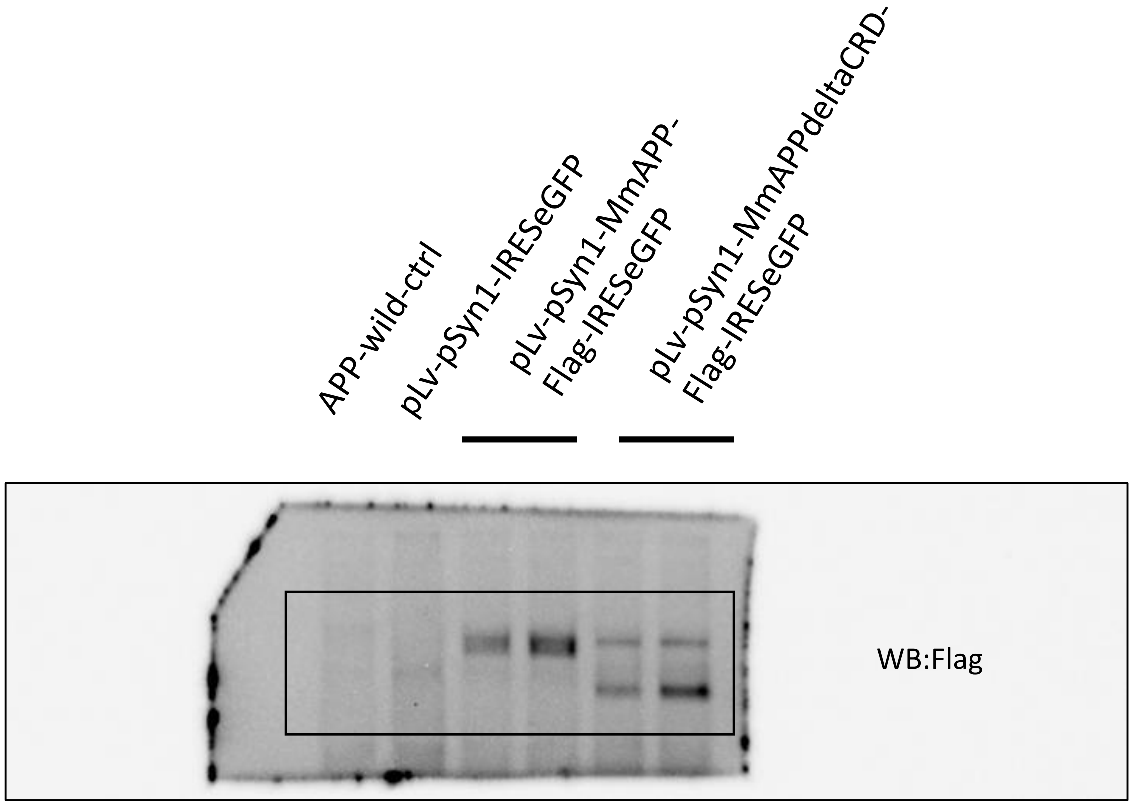

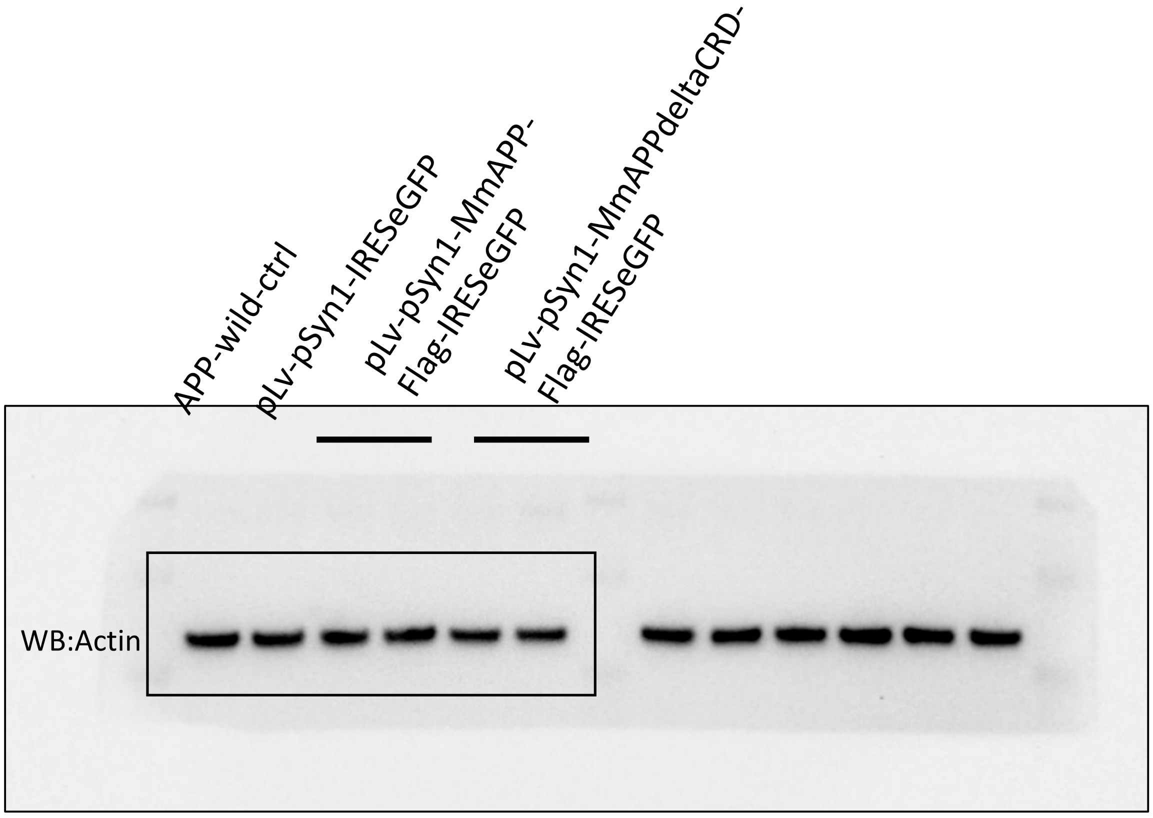

Figure S9C-Source data

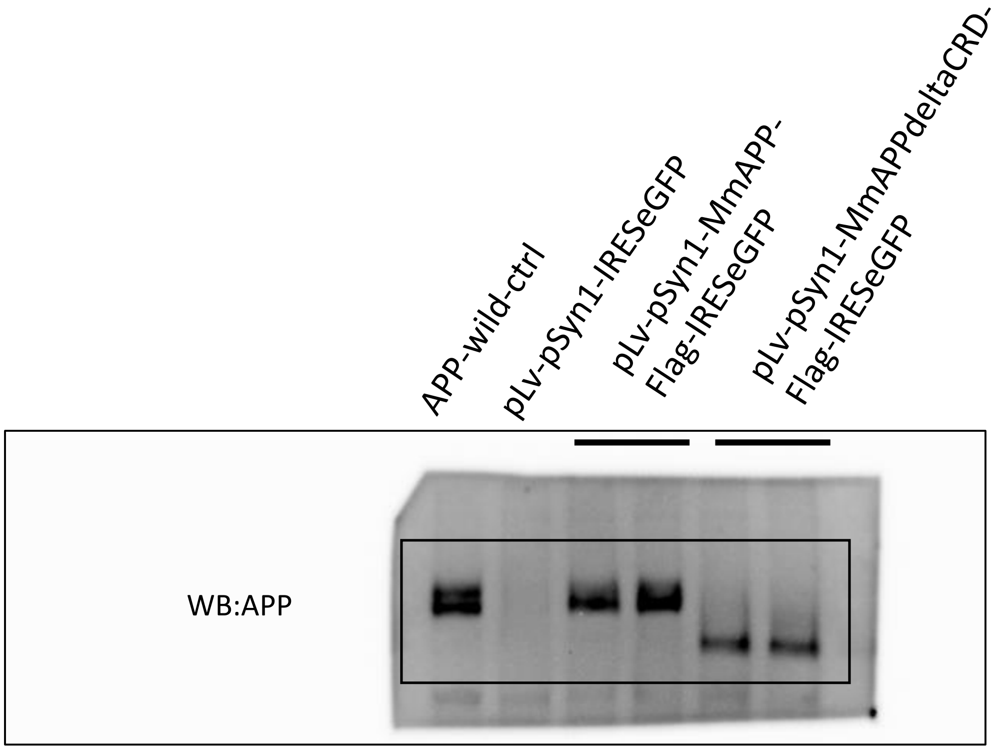

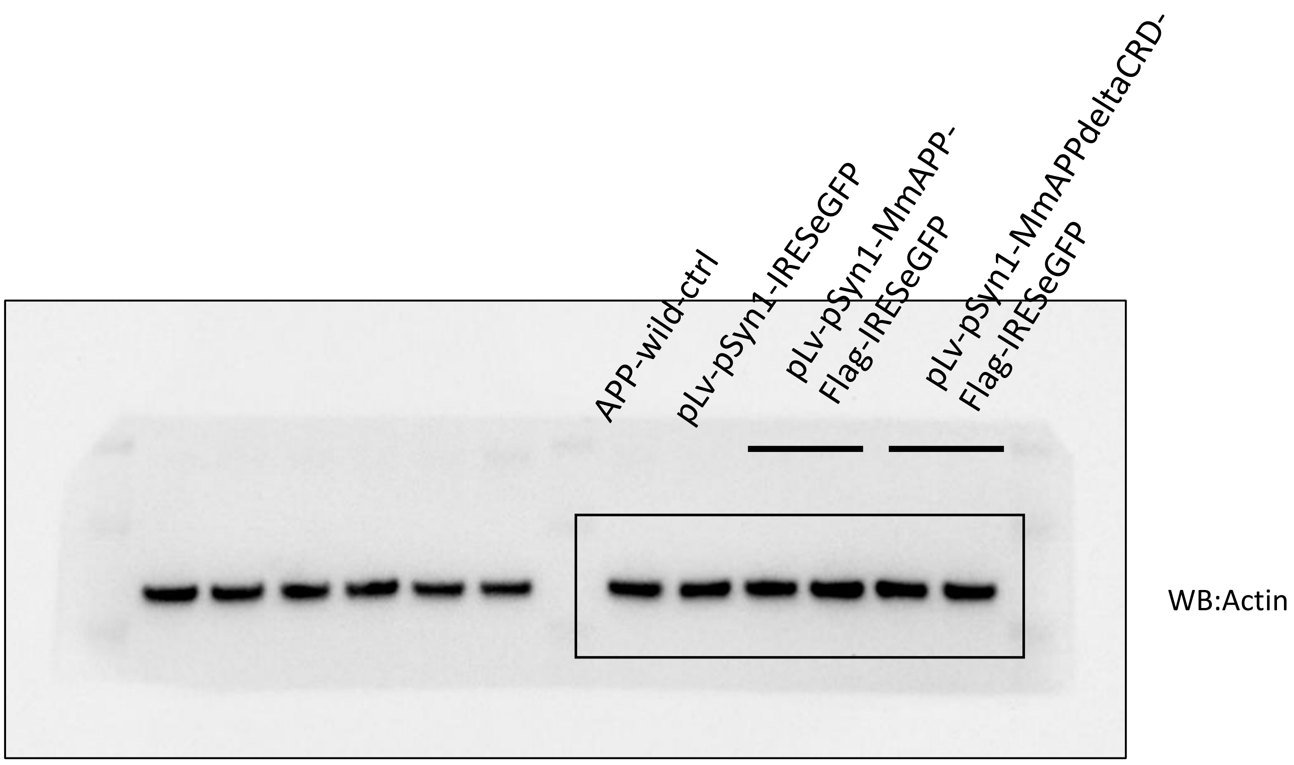

Figure S14-Source data

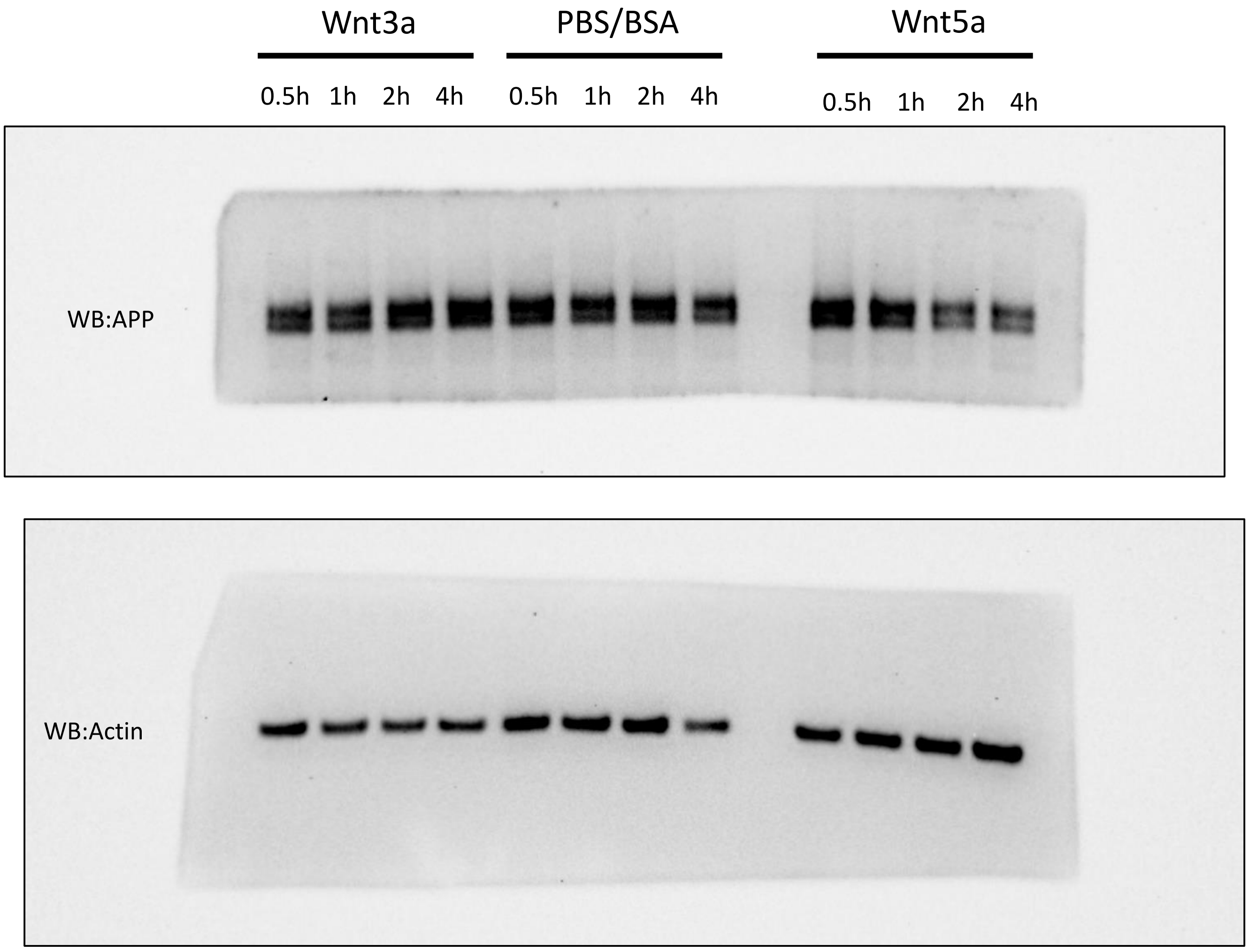

Supplement: Figure 3—figure supplement 4—source data 1. [file elife-69199-fig3-figsupp4-data1.zip › Figure 3-figure supplement 4/Figure 3-figure supplement 4-Source data 1 labeled bands.pdf]
