# Supplementary material for: The amyloid precursor protein is a conserved Wnt receptor
Source: eLife. 2021 Sep 9;10:e69199. doi: 10.7554/eLife.69199 (PMC8437438; doi:10.7554/eLife.69199)

Figure 3A-Source data

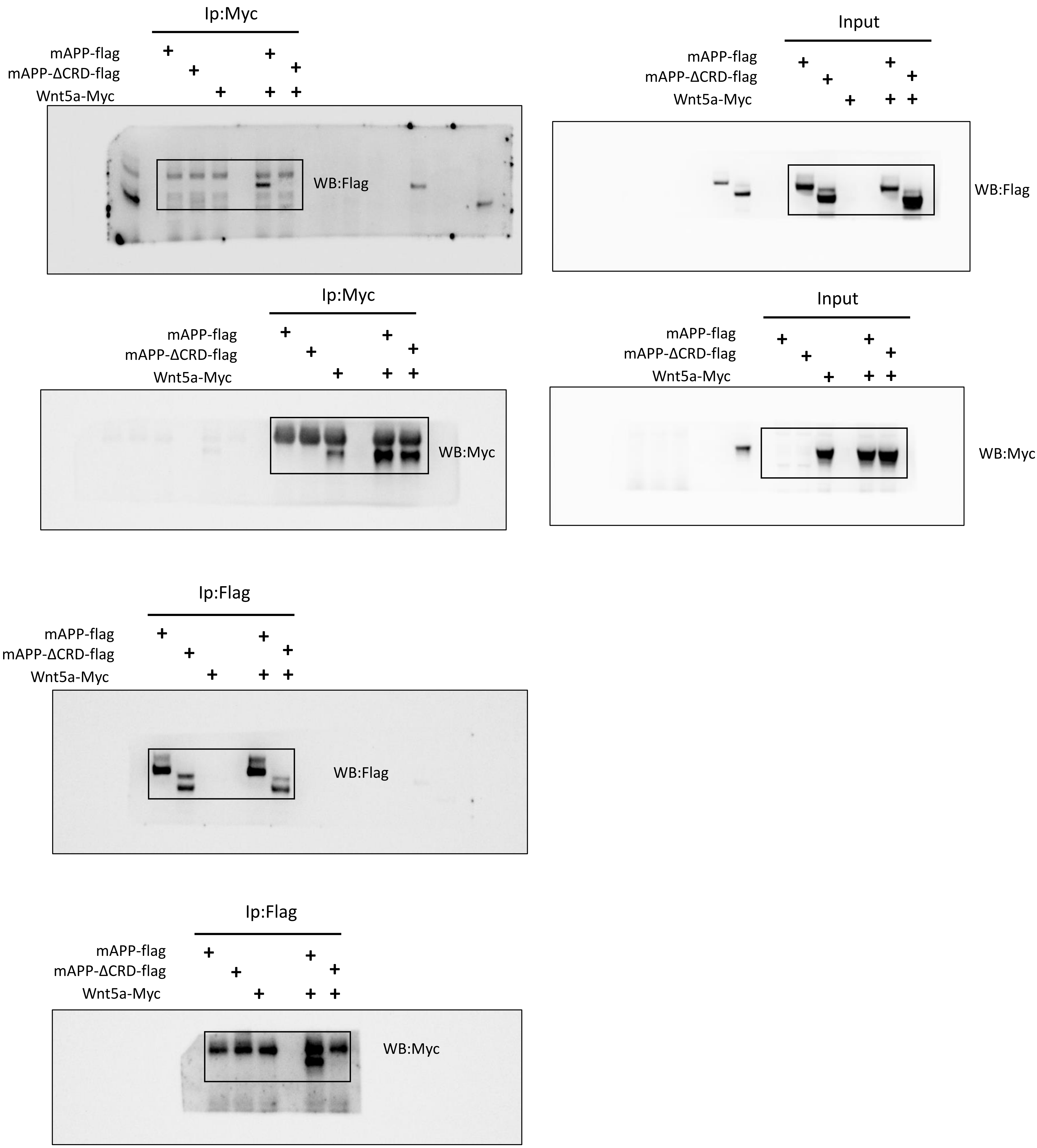

Supplement: Figure 3—source data 1. [file Figure 3A-Source Data 1 labeled bands.pdf › Figure 3A_Source Data/Figure 3A-Source Data 1 labeled bands.pdf]
